# Supplementary material for: The stigmatization of mental illness by mental health professionals: Scoping review and bibliometric analysis
Source: PLoS One. 2023 Jan 20;18(1):e0280739. doi: 10.1371/journal.pone.0280739 (PMC9858369; doi:10.1371/journal.pone.0280739)
Supplement: S1 Appendix — (DOCX) [file pone.0280739.s001.docx]

**Table Notes**

- Due to size constraints, the table was divided into multiple documents (12 documents in total). Within the table, each study appears in alphabetical order with respect to author names. Each table document is grouped in accordance with contiguous letters (e.g., A-B) that correspond to the alphabetisation within the table documents.
- The term psychiatrist refers to psychiatric registrars or full psychiatrists.
- In the analytical approaches column, a dash specifies when analytical approaches were not utilised.
- Specific mental disorders were reported in line with the terms used in the studies. If several different terms were employed for the same mental disorder, just one of these terms was used within the table.
- In the disorders column, the way that mental disorders were presented to participants was included in parentheses. Mental disorders were either presented with a ‘label’ (e.g., borderline personality disorder) or by portraying the features of mental illness in some way (e.g., with a vignette). In the table, the latter was denoted by either the term “description” or “presentation”.
- Where necessary, the variables and measures column included factors, items, and predictor variable levels for clarity. The factors, items, and levels appear under the variables and measures in the form of hierarchical indentations. Factors and items are included only once throughout the table for identical scales.
- In English, some items had grammatical errors likely due to some articles being translated from another language to English. The errors in these items were corrected in the table for intelligibility.

**Abbreviations**

**Mental Disorders**

- ADHD = Attention deficit hyperactivity disorder
- BPD = Borderline personality disorder
- GAD = Generalized anxiety disorder
- MDD = Major depressive disorder
- MUS = Medically unexplained symptoms
- OCD = Obsessive compulsive disorder
- PTSD = Posttraumatic stress disorder
- SUD = Substance use disorder

**Scales**

- ADSHQ = Attitudes towards Deliberate Self-Harm Questionnaire
- AMI = Attitude towards Mental Illness
- AMIQ = Attitudes of Mental Illness Questionnaire
- APDQ = Attitude to Personality Disorder Questionnaire
- ASMI = Attitudes to Severe Mental Illness
- AQ = Attribution Questionnaire
- ASQ = The Attitudes and Skills Questionnaire
- ATAMHS = Attitudes Toward Acute Mental Health Scale
- BMI = Beliefs Toward Mental Illness
- CAMI = Community Attitude Toward Mental Illness
- CAMI-I = Italian version of the Community Attitudes towards the Mentally Ill inventory
- CAQ = Client Attitude Questionnaire
- CASA = Community Attitudes Toward Substance Abusers
- CLAS-MI = Community Living attitudes Scale-Mental Illness
- DAQ = Depression Attitude Questionnaire
- DSS = Depression Stigma Scale
- FABI = Fear and Behavioural Intentions toward the mentally ill questionnaire
- GNAT = Go/No-Go Association Task
- GSD =Greek Social Distance
- IAT = Implicit Association Test
- IDR-R = Insanity Defence Attitude-Revised
- IMI = Impact Message Inventory
- IRI = Interpersonal Reactivity Index
- MCRS = Medical Condition Regard Scale
- MISM-P = Mental Illness Microaggression Scale-Perpetrator Version
- OMI = Opinions about Mental illness
- OMS-HC = Opening Minds Scale for Health Care Providers
- QMEE = Questionnaire Measure of Emotional Empathy
- RAQ = Recovery Attitudes Questionnaire
- RIBS = Reported and Intended Behavior Scale
- SAAS = Substance Abuse Attitude Survey
- SAPDI = Staff Attitude to Personality Disorder Interview
- SAS = Stress Appraisal Scale
- SDSJ = Japanese language version of the Social Distance Scale

**Other**

- MHA = Mental Health Act
- GPs = General practitioners
- USA = United States of America
