# Supplementary material for: The stigmatization of mental illness by mental health professionals: Scoping review and bibliometric analysis
Source: PLoS One. 2023 Jan 20;18(1):e0280739. doi: 10.1371/journal.pone.0280739 (PMC9858369; doi:10.1371/journal.pone.0280739)
Supplement: S2 Appendix — (DOCX) [file pone.0280739.s002.docx]

| **Authors (year)** | **Populations**  **(countries)** | **Research methods** | **Analytical approaches** | **Disorders** | **Variables and measures** | **Findings** |
| --- | --- | --- | --- | --- | --- | --- |
| Adams et al. (2016) | Clinical psychology professionals  Counselling psychology professionals  School/educational psychology professionals  Social work professionals  Marriage and family therapists  Pastoral counsellors  Unspecified medical professionals  Management professionals  Art therapists  Other unspecified professionals |  |  |  |  | Nothing more was reported for this study as findings were not reported for mental health professionals separately. |
| Adewuya et al. (2017) | Unspecified physicians from primary care centres  Unspecified nurses from primary care centres  Midwives  Community health officers and community health extension workers  (Nigeria) | Cross-sectional survey  A vignette was used | - | Depression (description) | Causal attributions  Perceived dangerousness to other patients and staff  Social distance | Physicians were most inclined to attribute cause to psychosocial factors (e.g., lack of willpower), followed by biological factors (e.g., brain injury), followed by supernatural factors (e.g., god’s will).  More than half the physicians did not believe that the target would be a danger to other patients and staff.  For physicians, social distance increased with the level of intimacy required from the specified relationships. Less than half of the physicians displayed high overall social distance.  Other relevant findings were excluded from this table as they were not reported for physicians separately. |
| Adjorlolo (2018) | Mental health nurses  (Ghana) | Cross-sectional survey  A schizophrenia vignette was included | Correlation analysis | Mental illness in general (label) | CAMI questionnaire  Open-minded and pro-integration  Residents should accept the location of mental health facilities in their neighbourhood to serve the needs of the local community  Most persons who were once patients in a mental hospital can be trusted as babysitters  Locating mental health services in residential neighbourhoods does not endanger local residents  Mental health facilities should be kept out of residential neighbourhoods  Having mental patients living within residential neighbourhoods might be a good therapy, but the risks to the residents are too great  Local residents have good reason to resist the location of mental health services in their neighbourhood  Mental illness is an illness like any other  We need to adopt a far more tolerant attitude towards the mentally ill in our society  The mentally ill are far less of a danger than most people suppose  Fear and avoidance  Community mental health ideology (lack of intention to segregate people with mental illness)    IDA-R questionnaire  Strict liability (agreement with punishing people who commit criminal acts, regardless of their degree of mental disturbance)  Unprofessional behaviour and safety concern (criminals are acquitted dishonestly due to the insanity defence, and these criminals are a threat to the public)  Expression of sympathy (the opposite of strict liability)  Punitiveness (amount of punishment deemed appropriate for offenders)  Conviction proneness (likelihood of convicting or acquitting suspects) | Participants displayed more positive than negative attitudes on the CAMI.  CAMI total scores were significantly negatively correlated with punitiveness. CAMI total scores were not found to be significantly correlated with IDA-R total scores and conviction proneness.  Individual CAMI factors were not found to be significantly associated with every other variable. However, each factor of the IDA-R, punitiveness, and conviction proneness were found to be significantly correlated with at least one factor of the CAMI. The only exception to this was unprofessional behaviour and safety concern. This factor of the IDA-R was not found to be significantly correlated with any factors of the CAMI. |
| Aftab et al. (2020) | Professionals from the following fields  Psychiatry  Psychology  Family medicine  Neurology  Neuroscience  Geriatric medicine  Other unspecified  Unspecified nurses from psychiatry units  Social workers  Trainee psychiatrists  Trainee psychologists  Trainee family medicine practitioners  Trainee neurologists  Trainee neuroscientists  Other research trainees  Medical students  (USA) | Cross-sectional survey | - | Mental illness in general (label) | All mental disorders are diseases | The nurses and social workers agreed slightly more that all mental disorders are diseases.  Other relevant findings were excluded from this table as they were not reported for mental health professionals separately. |
| Ahmead et al. (2010) | A psychologist  Social workers  Occupational therapists  Unspecified nurses working in a psychiatric hospital  Unspecified physicians working in a psychiatric hospital  Other unspecified mental health professionals  (Palestine) | Cross-sectional survey | - | Mental illness in general (label)  Substance and alcohol abuse (label)  Schizophrenia (label) | ATAMHS 33 (only items relevant to stigmatisation were included in this table)  Safe-dangerous semantic differential  Adult-child semantic differential  Mature-immature semantic differential  Optimistic-pessimistic semantic differential  Cold hearted-caring semantic differential  Polite-rude semantic differential  Harmful-beneficial semantic differential  Clean-dirty semantic differential  Psychiatric illness deserves as much attention as physical illness  Mental illnesses are caused by genetic factors  Mental illness is the result of adverse social circumstances  Many normal people would become mentally ill if they had to live in a very stressful situation.  Alcohol abusers have no self-control  Members of society are at risk from mentally ill people  Mentally ill patients have no control over their emotions  Patients who abuse substances should not be admitted to acute wards  Patients with chronic schizophrenia are incapable of looking after themselves  Depression occurs in people with a weak personality  The cause of many psychological problems is bad nerves  Patients with mental illnesses are more likely to harm someone else than themselves  Those with a psychiatric history should never be given a job with responsibility  Violence mostly results from mental illness  Psychiatric patients are generally difficult to like  People are born vulnerable to mental illness  It is hard to help patients who are emotionally disturbed | Proportions of negative responses to the semantic differentials ranged from less than half to more than half (neutral responses were available).  Most participants agreed that psychiatric illness deserves as much attention as physical illness (neutral responses were available).  Most participants agreed that mental illness is caused by genetic factors, adverse social circumstances, and stressful situations (neutral responses were available).  Most participates responded negatively to the remaining items (neutral responses were available). However, the last 9 items listed under ATAHMHS 33 showed divided opinion (i.e., either a large proportion of neutral responses or little difference in the proportions of negative and positive responses). |
| Ahn et al. (2009) | Psychologists  Psychiatrists  Social workers  Psychology interns and clinical graduate students  Social work fellows  (USA) | Cross-sectional survey | Correlation analysis  Between-groups ANOVA | Mental retardation (label)  Schizophrenia (label)  Bipolar I disorder (label)  GAD (label)  Alcohol abuse (label)  MDD (label)  Adjustment disorder (label)  Narcissistic personality disorder (label)  Bulimia nervosa (label)  A range of unspecified mental disorders (labels)  Autistic disorder (label)  Asperger disorder (label)  Pervasive developmental disorders (label)  Dementia of the Alzheimer’s type (label)  Dementia (label)  Bipolar II disorder (label)  Delirium (label)  Schizoaffective disorder (label)  ADHD (label)  Cyclothymic disorder (label)  OCD (label)  Alcohol dependence (label)  Substance dependence (label)  Opioid dependence (label)  Cocaine dependence (label)  Cannabis dependence (label)  Conduct disorder (label)  Schizotypal personality disorder (label)  Dysthymic disorder (label)  Panic disorder with agoraphobia (label)  Schizoid personality disorder (label)  Substance abuse (label)  Opioid abuse (label)  Cannabis abuse (label)  Cocaine abuse (label)  Hallucinogen abuse (label)  Obsessive compulsive personality disorder (label)  Paranoid personality disorder (label)  Oppositional defiant disorder (label)  Antisocial personality disorder (label)  BPD (label)  Anorexia nervosa (label)  PTSD (label)  Social phobia (label)  Avoidant personality disorder (label)  Histrionic personality disorder (label)  Dependent personality disorder (label)  Adjustment disorder with mixed anxiety and depressed mood (label)  Adjustment disorder with depressed mood (label)  Bereavement (label) | Causal attributions  Importance of the different causal attributions  Profession | For the relevant participants and the first nine mental disorders in the disorders column, some of the mental disorders were attributed more to biological causes (e.g., mental retardation, schizophrenia), and some were attributed more to psychological and environmental causes (e.g., adjustment disorder, narcissistic personality disorder). Inferential statistics were not applied to differences between these mental disorders.  For the relevant participants and the first nine mental disorders in the disorders column, beliefs about the biological cause (e.g., heredity/genetics) of mental illness were significantly negatively correlated with beliefs about psychological causes (e.g., personality factors) and environmental causes (e.g., stressful life events). Beliefs about the psychological causes of mental illness were significantly positively correlated with beliefs about environmental causes. Also, beliefs about the importance of biological causes of mental illness were significantly negatively correlated with beliefs about the importance of psychological causes and environmental causes.  Beliefs about the importance of psychological causes of mental illness were significantly positively correlated with beliefs about the importance of environmental causes.  For all of the disorders, psychiatrists and psychologists rated the cause of mental illness to be significantly more biological than social workers. Statistics for multiple comparisons were not reported. No significant differences were found by profession for psychological or environmental causal attributions.  Other relevant findings were excluded from this table as they were not reported for mental health professionals separately. |
| Al-Atram (2018) | Family medicine practitioners  GPs  Unspecified medical specialists  (Saudi Arabia) | Cross-sectional survey | - | Anxiety and depression (label) | Attitudes (items were not specified) | Family medicine practitioners expressed positive attitudes.  Other relevant findings were excluded from this table as they were not reported for mental health professionals separately. |
| Album et al. (2017) | GPs  Other physicians  Hospital doctors  Administrators  Professors  Medical students |  |  |  |  | Nothing more was reported for this study as findings were not reported for GPs separately. |
| Album & Westin (2008) | GPs  Other physicians  Hospital consultants  Chiefs of service  Hospital administrators  Medical students  (Norway) | Cross-sectional survey | - | Schizophrenia (label)  Anorexia (label)  Depressive neurosis (label)  Anxiety neurosis (label) | Perceived general prestige in the medical community | For the GPs the mental disorders had little prestige.  The following mental disorders are listed from least to most prestigious for the GPs.  Anxiety neurosis  Schizophrenia  Depressive neurosis  Anorexia  None of the differences reported between mental disorders were analysed with inferential statistics.  Other relevant findings were excluded from this table as they were not reported for GPs separately. |
| Allen et al. (2019) | Family physicians  Internists |  |  |  |  | Nothing more was reported for this study as findings were not reported for family physicians separately. |
| Almeida et al. (2021) | Psychiatrists  GPs  Trainee psychiatrists  Trainee GPs |  |  |  |  | Nothing more was reported for this study as findings were not reported for mental health professionals separately. |
| Araten-Bergman & Werner (2017) | Social workers  (Israel) | Cross-sectional survey  A vignette was used | Correlation analysis  Independent samples t-test  Multiple regression analysis | Dual diagnosis of schizophrenia and intellectual disability (label) | Causal attributions  Perceived personal responsibility  Perceived dangerousness  Emotions  Anger  Fear  Pity  Behavioural intentions  Segregation  Avoidance  Coercion  Helping  Sex  Age  Years of education (both general education and tertiary education in social work)  Ethnicity  Familiarity with individuals with disabilities | Participants were most inclined to attribute cause to biology, followed by how the target was raised, followed by internal or personal factors (e.g., lack of willpower).  Participants attributed little responsibility to the target for their mental disorders.  Participants perceived little danger in the target.  Participants experienced more pity towards the target than fear or anger.  The findings suggest inconsistent patterns of behavioural intentions (e.g., participants were inclined to help, but also be coercive).  All causal attributions were significantly positively correlated with each other.  Biological attributions were a significant predictor of more pity. Biological attributions were not found to be a significant predictor of perceived personal responsibility, perceived dangerousness, behavioural intentions, or any other emotions. Internal attributions were a significant predictor of more perceived personal responsibility, and perceived dangerousness, but where not found to be a significant predictor of emotions or behavioural intentions. Attributing cause to how the target was raised was not found to be a significant predictor of perceived personal responsibility, perceived dangerousness, emotions, or behavioural intentions.  Perceived personal responsibility was not found to be a significant predictor of perceived dangerousness, any emotions, or any behavioural intentions.  Perceived dangerousness was a significant predictor of more anger, fear, segregation, avoidance, and coercion. Perceived dangerousness was not found to be a significant predictor of pity or helping.  Pity was not found to be significantly correlated with anger or fear. However, fear was significantly positively correlated with anger.  Anger was found to be a significant predictor of less helping, but was not found to be a predictor of any other behavioural intention. Fear was not found to be a significant predictor of any behavioural intentions. Pity was found to be a significant predictor of more coercion and more helping, but was not found to be a significant predictor of segregation or avoidance.    Sex was not found to have a significant impact on any of the stigmatisation constructs.  Age and years of education were not found to be significant predictors of any of the stigmatisation constructs (causal attributions were not included in the regression).  Non-Jewish participants, compared to Jewish participants, reported significantly more agreement with internal causation. Ethnicity did not have a significant impact on the other causal attributions. Being non-Jewish was a significant predictor of decreased perceived personal responsibility and increased perceived dangerousness and segregation. Being non-Jewish was not found to be a significant predictor of any emotional reactions or any of the other behavioural reactions.  Familiarity with individuals with disabilities did not have a significant impact on any of the stigmatisation constructs. |
| Arbanas et al. (2019) | Unspecified nurses from a general hospital and a psychiatric hospital  Unspecified medical doctors from a general hospital and a psychiatric hospital  Lay people |  |  |  |  | Nothing more was reported for this study as findings were not reported for mental health professionals separately. |
| Artzi-Medvedik et al. (2012) | Female psychiatric nurses  Female midwives from general medical and psychiatric centres  Female postpartum nurses from general medical and psychiatric centres  (Israel) | Cross-sectional survey  A vignette was used | - | Schizophrenia (description and a possible label) | AQ-27 (items were not specified)  Familiarity with mental illness  Perceived personal responsibility  Pity  Anger  Fear  Helping  Coercion-segregation  Attitudes towards breastfeeding among women with schizophrenia (items were not specified) | Most of the psychiatric nurses displayed an overall lack of stigmatisation towards the target on the AQ-27, and positive attitudes towards breastfeeding among women with schizophrenia.  Other relevant findings were excluded from this table as they were not reported for mental health professionals separately. |
| Arvaniti et al. (2009) | Psychiatrists  Internists  Surgeons  Doctors working in laboratories  Unspecified nurses  Other unspecified health professionals  Medical students |  |  |  |  | Nothing more was reported for this study as findings were not reported for psychiatrists separately. |
| Avery et al. (2013) | Psychiatrists  (USA) | Cross-sectional survey | Independent samples t-test  Between-groups ANOVA | Schizophrenia (label)  Polysubstance dependence (label)  Major depression (label)  Comorbid polysubstance dependence and schizophrenia (label)  Other unspecified patients (label) | MCRS  I prefer not to work with patients like this  Patients like this irritate me  I enjoy giving extra time to patients like this  Patients like this are particularly difficult for me to work with  Working with patients like this is satisfying  I feel especially compassionate toward patients like this  I wouldn’t mind getting up on call nights to care for patients like this  I can usually find something that helps patients like this feel better  There is little I can do to help patients like this  Insurance plans should cover patients like this to the same degree that they  cover patients with other conditions  Treating patients like this is a waste of medical dollars  Prognosis  Specialty  Community psychiatrists  Addiction psychiatrists | Overall, participants displayed more positive than negative attitudes towards all the mental disorders on the MCRS. The only exceptions to this were community psychiatrists responded neutrally to three items for polysubstance dependence, and displayed a more negative attitude on the item I wouldn’t mind getting up on call nights to care for patients like this for polysubstance dependence (scores for each individual item were only reported for polysubstance dependence and schizophrenia).  The following are the mental disorders listed from least positive attitudes to most positive attitudes on the MCRS for community psychiatrists.  Polysubstance dependence  Comorbid polysubstance dependence and schizophrenia  Depression  Schizophrenia  The difference between comorbid polysubstance dependence and schizophrenia, and schizophrenia was statistically significant.  The other differences were not assessed with inferential statistics.  The following are the mental disorders listed from least positive attitudes to most positive attitudes on the MCRS for addiction psychiatrists.  Schizophrenia  Polysubstance dependence and depression  Comorbid polysubstance dependence and schizophrenia  The only item on which schizophrenia and polysubstance dependence elicited the same score, was insurance plans should cover patients like this to the same degree that they cover patients with other conditions. The difference between comorbid polysubstance dependence and schizophrenia, and polysubstance dependence was statistically significant. The other differences were not assessed with inferential statistics.  Community psychiatrists had significantly more positive attitudes towards schizophrenia, and addiction psychiatrists had significantly more positive attitudes towards polysubstance dependence. However, not all individual items were statistically significant. Speciality was not found to have a significant impact on attitudes for the other mental disorders.  Prognosis was assessed with schizophrenia and polysubstance dependence only.  Participants were more optimistic about the way people with schizophrenia and people with polysubstance dependence would respond to treatment.  Community psychiatrists were less optimistic regarding how people with polysubstance dependence would respond to treatment, compared to people with schizophrenia. This was the opposite for addiction psychiatrists. These differences between mental disorders were not assessed with inferential statistics.  Addiction psychiatrists were significantly more optimistic about the way people with polysubstance dependence would respond to treatment. Speciality was not found to have an impact on prognosis for schizophrenia. |
| Back et al. (2009) | Psychiatrists  Social workers  Counsellors  Clinical psychologists  Unspecified nurses from mental health organisations  Other unspecified physicians from mental health organisations  Other unspecified clinicians from mental health organisations  (USA) | Cross-sectional survey | Between-groups ANOVA  Correlation analysis | SUD (label)  PTSD (label)  Dual diagnosis of SUD and PTSD (label) | Perceived difficulty in treating the particular diagnoses  Gratification experienced when treating the particular diagnoses | Generally, SUD and PTSD were perceived as not difficult to treat, whereas a dual diagnosis of SUD and PTSD was perceived as either not difficult to treat or a great deal difficult to treat.  PTSD was perceived as the least difficult to treat, followed by SUD, followed by a dual diagnosis of SUD and PTSD. Type of mental disorder was found to have a significant impact on perceived difficulty. However, multiple comparisons were not applied.  There was a significant negative relationship between gratification and perceived difficulty for all mental disorders. |
| Bailey (1969) | Social workers  (USA) | Longitudinal survey  An intervention was used | - | Alcoholism (label) | Causal attributions  An alcoholic is harder to relate to than an individual whose illness is not self-inflicted  It is hard to be truly accepting of alcoholics, when one considers how seriously they damage their children  Prognosis with treatment  The immediacy of an alcoholic’s demands makes it very difficult to maintain a professional relationship with him  It is difficult to be treatment-minded with alcoholics, because so much of their behaviour is narcissistic | Participants attributed alcoholism to a range of causes with varying proportions. Underlying emotional problems and lack of will power were endorsed more frequently than physiological disposition.  Before the alcoholism training program roughly half of the participants agreed that an alcoholic is harder to relate to than an individual whose illness is not self-inflicted.  Before the alcoholism training program just under half of the participants agreed that it is hard to be truly accepting of alcoholics, when one considers how seriously they damage their children.  Before alcoholism training, most participants agreed that the majority of alcoholics can recover with treatment.  Before alcoholism training about half of the participants agreed that the immediacy of an alcoholic’s demands makes it very difficult to maintain a professional relationship with him.  Before alcoholism training less than half of participants agreed that it is difficult to be treatment-minded with alcoholics, because so much of their behaviour is narcissistic.  Following alcoholism training, stigmatisation decreased for some measures and increased for others. None of the differences between before and after training were assessed with inferential statistics. |
| Baker et al. (2005) | Unspecified nurses from mental health facilities  (England) | Cross-sectional survey | - | Mental illness in general (label)  Alcohol abuser (label)  Substance abusers (label)  Chronic schizophrenia (label)  Depression (label) | ATAMHS 33  Care or control  Alcohol abusers have no self-control  Patients with chronic schizophrenia are incapable of looking after themselves  Members of society are at risk from the mentally ill  Mentally ill patients have no control over their emotions  Nurses should not talk to patients about their delusions  Deliberate self-harm more often happens when other people are around  Depression occurs in people with a weak personality  The cause of many psychological problems is bad nerves  Patients with mental illnesses are more likely to harm someone else than themselves  Acute wards are little more than prisons  Mental illness is the result of adverse social circumstances  Many normal people would become mentally ill if they had to live in a very stressful situation  Semantic differentials  Polite-rude  Harmful-beneficial  Cold-hearted-caring  Clean-dirty  Mature-immature  Optimistic-pessimistic  Safe-dangerous  Therapeutic perspective  Those with a psychiatric history should never be given a job with responsibility  Those who attempt suicide leaving them with serious liver damage should not be given treatment  Violence mostly results from mental illness  Psychiatric patients are generally difficult to like  Patients who abuse substances should not be admitted to acute wards  Psychiatric treatments cause patients to worry too much about their symptoms  Hard to help  It is difficult to negotiate care plans with patients in acute environments  It is hard to help patients who are emotionally disturbed  Psychiatric drugs are used to control disruptive behaviour  Mental illnesses are genetic in origin  Positive attitudes  Psychiatric illness deserves as much attention as physical illness  The manner in which you talk to patients affects their mental state  People are born vulnerable to mental illness  Adult–child | The only score from the ATAMHS 33 that was interpretable with the information provided was the hard to help factor score. For this factor, participants expressed roughly neutral responses. |
| Bander et al. (1987) | Psychiatrists  Surgeons  Internists  (USA) | Cross-sectional survey | - | Alcoholism (label) | A measure of attitudes towards alcoholism that uses semantic differentials  Tense-relaxed  Immoral-moral  Weak willed-strong willed  Immature-mature  Easily recognised based on appearance-not easily recognised based on appearance  Foolish-wise  Undependable-dependable  Patient does not want help-patient does wants help  Patient is not treatable-patient is treatable  Patient is not curable-patient is curable  Patient is not motivated for recovery-patients is motivated for recovery  Pessimistic about treatment-optimistic about treatment | Relevant participants stigmatised alcoholism more with some items and less with other items.  Other relevant findings were excluded from this table as they were not reported for psychiatrists separately. |
| Bayar et al. (2009) | Psychiatrists  Psychiatry residents |  |  |  |  | Nothing more was reported for this study as findings were not reported for psychiatrists separately. |
| Beaulieu et al. (2017) | Family physicians  (Canada) | Experiment  An intervention was used | Multilevel modelling  Correlation analysis  Moderation analysis | Mental illness in general (label) | OMS-HC  Negative attitudes  I am more comfortable helping a person who has a physical illness than I am helping a person who has a mental illness  Despite my professional beliefs, I have negative reactions towards people who have mental illness  There is little I can do to help people with mental illness  More than half of people with mental illness don’t try hard enough to get better  Health care providers do not need to be advocates for people with mental illness  I struggle to feel compassion for a person with a mental illness  Health professionals’ own willingness to disclose or seek help for a mental illness  Preference for greater social distance  Confidence in managing mental illness  Sex  Size of the practice participants worked in (control variable) | In general, participants expressed positive attitudes towards mental illness at pre-test.  Findings regarding health professionals’ own willingness to disclose or seek help for a mental illness were not included in this table as this factor does not reflect endorsed provider-based stigma.  Overall participants did not express a preference for greater social distance at pre-test.  While controlling for size of practice, a significant interaction effect of time (i.e., pre and post training) and condition (i.e., intervention and control) was not found for negative attitudes.  While controlling for size of practice, there was a significant interaction between time and condition for social distance. For the skill-based training group, social distance decreased from pre-test to post-test and for the control group, social distance increased. This was all that was reported.  Confidence in managing mental illness was significantly negatively correlated with stigmatisation on the OMS-HC. This was moderated by sex, such that the relationship was stronger for males compared to females. This was all that was reported. |
| Berg et al. (1978) | Social workers  Unspecified nurses from medical clinics and alcohol treatment agencies  Unspecified physicians and PhDs from medical clinics and alcohol treatment agencies  Case aides and counsellors  Aides  Health aides  (USA) | Cross-sectional survey | - | Mental illness in general (label) | OMI scale  Authoritarianism (characterised by the following)  A view of the mentally ill as a class inferior to normals requiring coercive handling  Submission to and respect for authority are important virtues  Benevolence (characterised by the following)  Kindness toward unfortunates  Mental patients are not failures but more like children who must be watched  Mental patients are the responsibility of society  Mental hospitals are like prisons  Mental hygiene ideology (characterised by the following)  Mental illness is an illness like any other  The mentally ill are capable of skilled labour  The mentally ill should not be in prison-like institutions  Social restrictiveness (characterised by the following)  Small children should not be allowed to visit people with mental illness  A woman would be foolish to marry a man who has had a severe mental illness  All patients in mental hospitals should be prevented from having children  Interpersonal aetiology (characterised by the following)  Mental illness arises from interpersonal experience  Successful people rarely become mentally ill  Profession  Agency type | Social workers and unspecified nurses from alcohol treatment agencies mostly expressed positive attitudes. However, these participants had less positive attitudes for the mental hygiene ideology factor.  Social workers and unspecified nurses from alcohol treatment agencies disagreed more with interpersonal aetiology.  Compared to social workers, nurses from alcohol treatment agencies expressed more positive attitudes for benevolence and mental hygiene ideology, and expressed more negative attitudes for authoritarianism and social restrictiveness. Also, nurses from alcohol treatment agencies agreed more with interpersonal aetiology compared to social workers. These differences were not examined with inferential statistics.  Social workers from alcoholism treatment agencies expressed more positive attitudes than social workers from medical clinics for two OMI factors. However, social workers from alcoholism treatment agencies expressed more negative attitudes than social workers from medical clinics for the other two OMI factors. These differences were not examined with inferential statistics for social workers separately.  Other relevant findings were excluded from this table as they were not reported for mental health professionals separately. |
| Beryl & Vollm (2018) | Psychologists  Psychiatrists  Social workers  Forensic psychiatric nurses  Unspecified education professionals and other unspecified allied health professionals  (England) | Cross-sectional survey | Between-groups ANOVA | Personality disorder (label) | APDQ  Enjoyment/loathing (warmth, liking and interest)  Security/vulnerability (fears, anxieties, and helplessness)  Acceptance/rejection (anger and a sense of difference)  Purpose/futility (pessimism)  Enthusiasm/exhaustion  Profession | Mental health professionals expressed more overall negative attitudes towards personality disorder.  Nurses and psychiatrists had significantly more negative overall attitudes than psychologists and social workers. Nurses and psychiatrists were not compared, and nor were psychologists and social workers.  Other relevant findings were excluded from this table as they were not reported for mental health professionals separately. |
| Bjorkman et al. (2008) | Psychiatric care nurses and assistant nurses  Somatic care nurses and assistant nurses |  |  |  |  | Nothing more was reported for this study as findings were not reported for psychiatric nurses separately. |
| Black et al. (2011) | Psychiatrists  Psychologists  Social workers  Unspecified nurses  Physician assistants  Psychiatry residents  Other unspecified clinicians  (USA) | Cross-sectional survey | - | BPD (label) | If I had a choice, I would prefer to avoid caring for a BPD patient  BPD patients intentionally manipulate others  It is easy for me to stereotype patients with BPD  I dislike BPD patients  The prognosis for BPD treatment is hopeless  Profession | Across the measures, mental health professionals mostly expressed more positive attitudes. However, psychiatrists and psychologists expressed more agreement with avoiding a BPD patient, psychologists expressed roughly neutral responses to BPD patients intentionally manipulating others, and psychiatrists expressed roughly neutral responses to stereotyping patients with BPD.  Social workers consistently expressed less stigmatisation across the measures compared to psychiatrists and psychologists. Also, psychologists expressed less stigmatisation than psychiatrists across all measures except one. In this case, psychiatrists expressed less stigmatisation than psychologists. Differences between the professions were not examined with inferential statistics for the mental health professionals separately.  Other relevant findings were excluded from this table as they were not reported for mental health professionals separately. |
| Blundell et al. (2012) | Psychiatric nurses  (England) | Semi-structured interviews  Repertory grid technique | Hierarchical cluster analysis  Principle components analysis | Depression (label)  Psychosis (label)  Personality disorder (label) | Stereotypes (e.g., good mother, dependent, unpredictable, difficult to get on with) | For most participants all of the mental disorders were construed as being closer to a pole that was characterised by negative stereotypes. The three mental disorders differed with respect to how close they were to different stereotypes. These differences were not quantified and were therefore unable to be interpreted clearly.  The only difference between the mental disorders that could be clearly examined regarded whether people with the specified mental disorders could be good mothers.  Personality disorder overlapped the most with good mothers, followed by psychosis, followed by depression. These differences were not assessed with inferential statistics. |
| Bodner et al. (2011) | Psychologists  Psychiatrists  Psychiatric nurses  (Israel) | Cross-sectional survey | MANOVA  Stepwise multiple regression analysis | BPD (label) | Empathy  I feel empathy toward  BPD patients  Taking care of BPD patients can evoke unfamiliar feelings  Patients with BPD evoke  parental emotions in me  I would like to relieve the  suffering of BPD patients  Treating a BPD patient is one of the most difficult treatments  Negative emotions  Anger  Lack of empathy  Impatience  Embarrassment  Helplessness  I do not like to treat BPD patients because they always tell me how miserable they are  When a BPD patient tries to harm himself/herself, I feel that the patient violates the therapeutic contract  Experienced treatment difficulties  It is easier for me to treat schizophrenic patients than BPD patients  Treating BPD patients can wear me out  It's difficult for me to treat BPD patients  Treatment sessions with BPD patients make me easily angry  I do not enjoy treating BPD patients because it is difficult to help them  I rarely pity BPD patients  Antagonistic judgments  A suitable setting for BPD patients is a closed psychiatric ward  The mental health services for BPD patients are sufficient enough  The treatment of BPD patients in hospital should be based on behavioural psychotherapy and  setting of limits  Psychotherapists should not be held responsible for a BPD patient dying by suicide  Psychotic manifestations among BPD patients are very common  While being in hospital, BPD patients demonstrate rapid mood changes and suicidal threats as a way to manipulate others  Treatment of BPD patients should be conducted by a psychiatrist  Treatment of BPD patients should be conducted by a social worker  BPD patients receive insufficient mental health services due to difficulties they create  Clinical depression among BPD patients may be followed by suicide  BPD should be defined as a psychiatric disorder because it is an Axis I disturbance  The mental health services that BPD patients receive are limited because of staff's lack of knowledge  Psychotic manifestations among BPD patients are in fact malingering  Profession  Seniority (levels were not specified)  Perceptions of suicidal tendencies  Wishing to improve treatment skills  Familiarity with therapies other than dialectical-behaviour therapy  Sex | In general, participants empathised with people with BPD.  Descriptive statistics for negative emotions and experienced treatment difficulties were not reported. Descriptive statistics were also not reported for the items of antagonistic judgements that were relevant to stigmatisation.  Controlling for seniority, nurses had significantly less empathy than psychiatrists and psychologists.  Whether there was a significant difference between psychiatrists and psychologists was not reported.  Controlling for seniority, no significant differences were found between professions for negative emotions and experienced treatment difficulties.  No significant differences were found between the levels of seniority for empathy or experienced treatment difficulties.  Seniority was a significant predictor of deceased negative emotions.  Perceiving people with BPD as being at risk of suicide was a significant predictor of negative emotions (controlling for wishing to improve treatment skills and seniority) and experiencing increased difficulty in treating people with BPD (controlling for familiarity with other therapies).  Wishing to improve treatment skills was a significant predictor of negative emotions (controlling for perceptions of suicidal tendencies and seniority).  Being familiar with other therapy methods was a significant predictor of experiencing increased difficulty in treating people with BPD (controlling for perceptions of suicidal tendencies).  Being a female, compared to being a male, was a significant predictor of increased empathy (controlling for antagonistic judgments).  Antagonistic judgments were a significant predictor of decreased empathy (controlling for sex). |
| Botega & Silveira (1996) | GPs  (Brazil) | Cross-sectional survey | - | Depression (label) | Causal attributions  Prognosis | Participants attributed depression to a range of causes with varying proportions. Recent misfortunes and biochemical abnormalities were the most likely causes, and poor stamina and deprivation in early life were the least likely causes (neutral responses were available).  Roughly half of the participants disagreed that depression reflects a characteristic response in patients which is not amendable to change, and depression seen in general practice improves without treatment (neutral responses were available). |
| Botega et al. (1992) | GPs  (UK) | Cross-sectional survey | Between-groups ANOVA | Depression (label) | Causal attributions  Prognosis  Amount of antidepressants prescribed  Time since receiving medical degree  Perceived frequency of depression in the clinic  Sex | Participants agreed more with biological and environmental causes of depression, and disagreed more with poor stamina being the cause of depression.  Participants agreed more with a positive prognosis.  Participants that attributed depression more to organic causes prescribed antidepressants significantly more than participants that attributed the cause of depression more to recent misfortunes.  Time since receiving medical degree and perceived frequency of depression was not found to have a significant impact on causal attributions.  Sex was not found to have a significant impact on biological causal attributions. |
| Brener et al. (2013) | Unspecified mental health professionals  (Australia) | Cross-sectional survey  IAT | Correlation analysis  Standardized multiple regression analysis | Mental illness in general (label)  Bipolar affected, schizophrenia affected, and PTSD affected (labels) | General attitudes  Implicit general attitudes/stereotypes (particular stereotypes were not specified)  Helping behaviour  Negative emotions (fear was the only example provided) | Participants had more general positive attitudes towards people with mental illness.  Participants held more implicit negative attitudes/stereotypes about the mental disorders than positive attitudes/stereotypes.  Participants were more certain than uncertain that they would help someone with a mental illness.  Participants expressed little negative emotion towards mental illness.  Implicit negative attitudes/stereotypes were a significant predictor of uncertainty in providing help to people with mental illness.  Implicit attitudes/stereotypes were not found to be a significant predictor of negative emotions.  Negative emotions were significantly positively associated with uncertainty in providing help to people with mental illness. |
| Bulbulia & Laher (2013) | Psychiatrists  (South Africa) | Semi-structured interviews | Thematic analysis | Mental illness in general (label) | Causal attributions  Islamic beliefs  General perceptions of mental illness | Participants emphasised biological factors in the aetiology of mental illness. Participants were less inclined to highlight the environmental, socio-cultural and religious aspects of mental illness.  It was suggested that Islamic beliefs influenced perceptions of mental illness. It was not clear as to how these beliefs influenced perceptions. |
| Burns et al. (2000) | GPs  Psychiatric nurses  Psychiatrists  Occupational therapists  A clinical psychologist  Social workers  (England) | Cross-sectional survey | - | Long-term psychotic disorders (label) | People with long-term psychotic disorders create a lot of work for a practice/are a major burden on a community health team  People with long-term psychotic disorders pose communication problems  People with long-term psychotic disorders are difficult to like  People with long-term psychotic disorders rarely cause difficulties for families or other carers  Prognosis whatever is done  Profession | Most participants did not stigmatise long-term psychotic disorders on most of the measures. There were only a couple of cases in which most participants stigmatised long-term psychotic disorders.  GPs were more likely to stigmatise long-term psychotic disorders than all the other professions combined. Differences between GPs and the other professions combined were not assessed with inferential statistics. The other professions were not compared with respect to stigmatisation. |
| Burroughs et al. (2006) | GPs  (England) | Semi-structured interviews | Thematic analysis | Depression (label) | Causal attributions  Prognosis  Age of the person with depression | Depression in late life was viewed by GPs as a normal response to loneliness and a reduction in function. Depression in late life was also seen as understandable and justified.  Some GPs expressed the belief that psychotherapy is unlikely to work for people with depression in late life compared to younger adults with depression. |
| Bushnell et al. (2005) | GPs  (New Zealand) | Cross-sectional survey | - | Mental illness in general (label) | I have no personal difficulties in dealing with mental health patients | Almost all participants agreed at least somewhat that they have no personal difficulties in dealing with mental health patients. |
