# Supplementary material for: The stigmatization of mental illness by mental health professionals: Scoping review and bibliometric analysis
Source: PLoS One. 2023 Jan 20;18(1):e0280739. doi: 10.1371/journal.pone.0280739 (PMC9858369; doi:10.1371/journal.pone.0280739)
Supplement: S3 Appendix — (DOCX) [file pone.0280739.s003.docx]

| **Authors (year)** | **Populations**  **(countries)** | **Research methods** | **Analytical approaches** | **Disorders** | **Variables and measures** | **Findings** |
| --- | --- | --- | --- | --- | --- | --- |
| Caldwell & Jorm (2001) | Mental health nurses  (Australia) | Experiment  Vignettes were used | Mann-Whitney *U*-test  Kruskal-Wallis test  Between-groups ANOVA | Depression (description)  Schizophrenia (description) | Prognosis  Perceived long-term positive and negative behavioural outcomes in the event that the target is getting help (some of which were stereotypes)  Whether the target is receiving professional help or not  Age  39 and younger  40-49  50 or older  Sex  Amount of contact with someone similar to the target in the vignette  Work setting  Community  Hospital  Size of community  Education environment  Hospital  University  Both hospital and university  Level of training  Undergraduate degree  Postgraduate diploma/certificate  Masters/PhD degree | Participants endorsed good and bad prognoses and long-term behavioural outcomes with varying proportions.  Generally, participants were more likely to report worse prognoses and long-term outcomes for schizophrenia compared to depression. This was not examined with inferential statistics.  Prognoses were more likely to be good if the target was receiving help. This was not assessed with inferential statistics.  Nurses that were 40 to 49 perceived slightly more positive long-term outcomes for schizophrenia compared to nurses that were 39 or younger. Nurses that were 50 or older perceived more negative long-term outcomes for schizophrenia compared to the other age groups. However, it was not clear if age had a significant impact on perceived long-term outcomes for schizophrenia, and multiple comparisons were not applied to these differences. All other age differences were not reported and not clearly assessed with inferential statistics.  Sex was not found have a significant impact on perceived long-term outcomes.  Amount of contact with a person similar to the target in the vignette, work setting, size of community, education environment, and level of training were not found to have a significant impact on perceived long-term outcomes.  All significant findings in this study were not accompanied by inferential statistics. |
| Calicchia (1981a) | Psychologists  Psychiatrists  Social workers  (USA) | Cross-sectional survey | Correlation analysis  Between-groups ANOVA | Mental illness in general (label) | Semantic differentials were used to measure attitudes  Worthy-unworthy  Safe-dangerous  Effectiveness-ineffectiveness  Understandable-mysterious  Social distance  Using the semantic differential dimensions and social distance, attitudes towards people in general were measured  Profession | Participants expressed stigmatising attitudes towards people with mental illness.  There was a significant positive correlation between attitudes towards the public and attitudes towards people with mental illness.  After controlling for attitudes towards people in general, each profession expressed significantly more negative attitudes than the other professions for at least one measure of stigmatisation. No profession consistently expressed significantly more negative attitudes than the other professions across the measures of stigmatisation and some differences were not found to be statistically significant. Statistics were not reported in this study for multiple comparisons. |
| Calicchia (1981b) | Psychologists  Psychiatrists  Social workers  Unspecified mental health students  Teachers  Lawyers  Engineers  (USA) | Cross-sectional survey | - | Mental illness in general (label) | Semantic differential dimensions (only the following examples were given for each dimension)  Evaluative (e.g., good-bad, worthy-unworthy)  Activity (e.g., safe-dangerous, predictable-unpredictable)  Potency (e.g., effective-ineffective, competent-incompetent)  Comprehension (e.g., understandable-incomprehensible, logical-mysterious)  Social distance | Generally, the mental health professionals expressed stigmatising attitudes towards mental illness.  Other relevant findings were excluded from this table as they were not reported for mental health professionals separately. |
| Cape et al. (2008) | GPs  (England) | Cross-sectional survey and unstructured interviews  Videos of mock consultations with patients with mental health problems were used | Correlation analysis | Depression (label) | DAQ  Treatment attitude (causal attributions including poor stamina/biochemical abnormality and antidepressants over psychotherapy)  Professional unease (discomfort and dissatisfaction working with depression)  Inevitable course of depression/pessimism about depression (depression is caused by deprivation in early life, is unchangeable, and is a normal part of getting old)  Identification of depression (depression is distinguishable from regular unhappiness)  Complexity of psychosocial explanations of mental health problems  Mention a greater number of hypothesised elements  for the patient’s difficulties  Link these elements to  describe how their interaction resulted in problems for the patient  Give alternative possible  explanations | Descriptive statistics were not reported for the DAQ.  There was a significant positive correlation between complexity of psychosocial explanations and positive attitudes toward depression. |
| Caplan et al. (2016) | Primary care physicians  A psychologist  A school psychologist  A medical psychologist  Social workers  Psychiatrists  An occupational therapist  A neuropsychologist and epidemiologist  Auxiliary nurses  Unspecified nurses  Directors of nursing, a subdirector, hospital administrators, a hospital director, and a regional health administrator  A paediatrician  Unspecified physicians  An unspecified physician and epidemiologist  An orthopaedic surgeon  A gastroenterologist  Medical students  (Dominican Republic) | Focus groups | Thematic analysis | Mental illness in general (label) | Perceived physical appearance  Perceived hygiene  Perceive strange expression  Perceived aggression | One primary care physician believed that people with mental illness have a poor physical appearance and lack good personal hygiene. Another primary care physician believed that people with mental illness have a strange expression in their eyes and seem aggressive.  Other relevant findings were excluded from this table as they were not reported for mental health professionals separately. |
| Caplan et al. (2018) | Primary care physicians  A psychologist  A school psychologist  A medical psychologist  Social workers  Psychiatrists  An occupational therapist  A neuropsychologist and epidemiologist  Auxiliary nurses  Unspecified nurses  Directors of nursing, a subdirector, hospital administrators, a hospital director, and a regional health administrator  A paediatrician  Unspecified physicians  An unspecified physician and epidemiologist  An orthopaedic surgeon  A gastroenterologist  Medical students  (Dominican Republic) | Focus groups | Thematic analysis | Mental illness in general (label) | Fear  Perceived aggression  Causal attributions | One psychologist stated that they experience fear in the company of people with mental illness due to their perception of people with mental illness being aggressive.  A social worker attributed mental illness to a lack of faith in god.  Other relevant findings were excluded from this table as they were not reported for mental health professionals separately. |
| Carroll (1993) | Counsellors  Social workers  Psychiatric nurses  General nurses  Prison nurse officers  (Scotland) | Cross-sectional survey | - | Mental illness in general (label)  Drug abusers (label)  Other unspecified patients (label) | A measure of attitudes towards drug abusers  Drug use can be treated successfully  A drug-using person who has had several relapses cannot be treated successfully  Street pushers are the initial source of drugs for young people  Angry confrontation is necessary in the treatment of drug users  Drug users are usually unconventional in dress and appearance  Any person who is receiving treatment in a residential setting should be discharged if discovered using illicit drugs  Drug users can be rehabilitated  Only disturbed people experiment with drugs  Drug users should be sterilised  Once someone is using drugs, there is very little that can be done  Drug use leads to mental illness  Most drug users have above average intelligence  Treatment of drug users should be through the prison system only  For most purposes the drug user can best be treated by a social worker  Parents should react with anger on discovering that their sons or daughters are using drugs  People who are ‘high’ on drugs should not be allowed into a drug treatment agency  Drug misuse is no different from any other physical illness  People who use drugs are sexually promiscuous  It is normal for a teenager to experiment with illicit drugs  Drug users should only be cared for in specialized units  People who use drugs are irresponsible  Most female drug users prostitute to support their habit  Drugs corrupt the young  Pregnant drug users should have an abortion  All drug users are criminals who prey on society  Drug users will 'grow out of it'  HIV testing should be compulsory for all drug users  Health and social services staff should be able to refuse to work with drug users  All drug users are a threat to society as potential AIDS carriers  Drug users are not as deserving of care as other patients  Profession | For the relevant items, most counsellors and social workers did not express stigmatisation (I don’t know was an available option).  Most counsellors and social workers disagreed that drug use leads to mental illness, and agreed that drug misuse is no different from any other physical illness (I don’t know was an available option).  Social workers expressed more overall negative attitudes than psychiatric nurses, followed by addiction counsellors. However, the impact of profession on attitudes was not examined with inferential statistics for the mental health professionals separately.  Other relevant findings were excluded from this table as they were not reported for mental health professionals separately. |
| Carr-Walker et al. (2004) | Psychiatric nurses  Prison officers  (England) | Cross-sectional survey and semi-structured interviews | - | Personality disorder (label) | APDQ  Enjoyment/loathing  Security/vulnerability  Acceptance/rejection  Purpose/futility  Enthusiasm/exhaustion  SAPDI | For the APDQ, psychiatric nurses expressed less positive attitudes on the enjoyment factor and more positive attitudes on the other factors.  As part of the SAPDI, psychiatric nurses expressed that personality disorder is either caused by nurture or a mixture of nature and nurture/were unsure. As part of the SAPDI, only a small proportion of the psychiatric nurses expressed that people with personality disorder have a warped and different perspective on the world that reflects incompetence. All other factors in the SAPDI were either unable to be interpreted with the information provided or were irrelevant to the stigmatisation of personality disorder.  Other relevant findings were excluded from this table as they were not reported for psychiatric nurses separately. |
| Ceuterick et al. (2020) | GPs  (Belgium) | Experiment  Videotaped interviews between a GP and a patient with major depression were used | MANOVA | Major depression (presentation) | Therapy adherence  The patient tells you  about all medications  and treatments he or she is using?  The patient tells you if he/she is not following the treatment plan?  The patient follows the treatment plan you recommend  The patient is likely to follow-up the referral advice  The patient will take the prescribed medicines in a correct manner  The patient is unlikely to follow up for clinic visits  The patient will stop his/he  medication as soon as he/  she feels better  Therapy optimism  It is unlikely that the  patient will improve  The patient will not understand GP’s recommendations  Optimistic about patient  chances of complete healing  The patient poses no difficult management problem  Client trustworthiness  The patient makes  unreasonable demands  The patient is exaggerating his pain  The patient does manipulate the office visit for secondary gain  Migration background  Native  Foreign decent  Asylum seeker | GPs expressed more positivity for therapy adherence and more overall positivity for therapy optimism. However, overall GPs responded neutrally towards a native target, and slightly more negatively towards foreign decent and asylum seeker targets for client trustworthiness.  Migration background was not found to have a significant impact on therapy adherence. For therapy optimism, GPs were significantly more positive overall towards the native and foreign decent targets compared to the asylum seeker target, and no significant difference was found between the two former targets. For client trustworthiness, GPs were significantly more positive overall towards the native target compared to the foreign decent and asylum seeker targets, and no significant difference was found between the two latter targets. |
| Chambers et al. (2010) | Mental health nurses  General nurses and other unspecified non-mental health nurses |  |  |  |  | Nothing more was reported for this study as findings were not reported for mental health nurses separately. |
| Chang et al. (2013) | Psychiatric nurses  Medical and surgical nurses, and other unspecified nurses  (Taiwan) | Cross-sectional survey | - | SUDs (label) | A measure of attitudes towards people with SUDs  Treatment necessity (perceptions regarding necessary treatments provided for clients with SUDs)  Treatment optimism (positive perceptions regarding treatment outcomes)  Acceptance (acceptance of SUDs within a continuum of normal human behaviour)  Stereotypes (the only examples provided were clients with SUDs are from low socio-economic groups, are unemployed, exhibit poor impulse control, and have emotional difficulties)  Moralism (moralistic perspectives regarding SUDs) | Psychiatric nurses expressed more overall positive attitudes.  Other relevant findings were excluded from this table as they were not reported for psychiatric nurses separately. |
| Charles & Bentley (2018) | Social work professionals  Marriage and family therapists  Counsellors  Psychology professionals  Unspecified nursing professionals that work in either mental health or inpatient facilities  Unspecified medical professionals that work in either mental health or inpatient facilities  Human services professionals  Support staff  Paraprofessionals  Other unspecified mental health and health professionals |  |  |  |  | Nothing more was reported for this study as findings were not reported for mental health professionals separately. |
| Chekuri et al. (2018) | Psychologists  Psychiatrists  Mental health nurses  Primary care physicians  Primary care nurses |  |  |  |  | Nothing more was reported for this study as findings were not reported for mental health professionals separately. |
| Chen et al. (2013) | Psychiatrists  (China) | Cross-sectional survey | - | Mental illness in general (label) | People with mental illness are treatable  People with mental illness should receive social security and free medical care  People with mental illness are more violent than the general population  All people with mental illness should receive violence risk assessments  It is difficult to manage forensic psychiatric patients  Separate legislation should be created regarding the disposal of forensic psychiatric patients  Forensic psychiatric patients can receive treatment in the community  A specific controlled community network needs to be established for the rehabilitation of forensic psychiatric patients  Offenders found not guilty by reason of insanity should be sent home under their guardians’ surveillance  Offenders found not guilty by reason of insanity should be treated in the custody of a forensic psychiatric hospital  Offenders found not guilty by reason of insanity should forcibly send them to a general psychiatric hospital  Offenders found not guilty by reason of insanity should be allowed to choose whether or not they received treatment in a hospital or as an outpatient  Offenders found not guilty by reason of insanity should be treated in prison | For some measures most of the participants expressed stigmatisation, whereas for other measures most participants expressed a lack of stigmatisation. |
| Chew-Graham et al. (2002) | GPs  (England) | Semi-structured interviews | Thematic analysis | Depression (label) | Causal attributions  Perceived reason for seeking treatment  Perceived burden caused by people with depression  Perceived difficulty  Frustration  People with depression are draining and tiresome  People with depression are unattractive and boring  Working with depression is a positive experience  Prognosis  Depression is time consuming | Participants reported that depression is caused by life stressors.  Participants suggested that some people with depression are seeking assistance not just for the treatment of their illness but also for an underlying reason, such as in order to avoid having to work.  Participants reported that people with depression are a psychological and professional burden.  One participant stated that you have to be robust enough to manage people with depression and if you are not, they can be very difficult.  Participants found people with depression frustrating and draining.  One participant stated that people with depression are unattractive, difficult, boring and tiresome.  Some participants found working with depression to be a positive experience and could see the potential for improvement.  However, these participants also reported being frustrated by some people with depression that resist treatment and continue to be depressed.  Some participants viewed depression as intractable and time consuming. |
| Chiba et al. (2016) | Social workers  Clinical psychologists  Psychiatrists  Occupational therapists  Unspecified nurses that work in psychiatric hospitals and community service agencies  Assistant nurses  Pharmacists  (Japan) | Cross-sectional survey | The analysis was not clear | Mental illness in general (label)  Schizophrenia (label) | Japanese version of the RAQ  Recovery is possible and needs faith  Recovery is difficult and differs among people  Positive attitudes scale  Ability and recovery of people with mental illness  Attitudes toward living alongside those people  Supportive helping behaviours  Social distance towards schizophrenia was measured with the SDSJ  Profession | Social workers, clinical psychologists, psychiatrists, and occupational therapists agreed more that people with mental illness can recover.  Scores on the positive attitudes scale and SDSJ were not reported.  Profession was not found to have a significant impact on overall RAQ scores. Inferential statistics were not reported for this.  Other relevant findings were excluded from this table as they were not reported for mental health professionals separately. |
| Chien et al. (2014) | Psychiatrists  Psychiatric nurses  Occupational therapists  Social workers  Psychologists  (China) | Cross-sectional survey | It was not clear which statistical analyses were used | Mental illness in general (label) | People with mental illness are unpredictable  People with mental illness are weird  People with mental illness are emotional  People with mental illness are bedraggled  People with mental illness are dangerous  People with mental illness are self-controlled  People with mental illness are stubborn  People with mental illness are healthy  People with mental illness are reasonable  People with mental illness are unreliable  People with mental illness are stupid  People with mental illness should be required to admit to a psychiatric hospital or unit  People with mental illness should have their license revoked  People with mental illness should have an abortion in the case of pregnancy  People with mental illness should have the right to vote  Profession  Years of experience as a mental health professional  Occupational characteristics (it was not clear what this meant) | For many measures, most participants expressed stigmatisation. For the other measures, either roughly half or most of the participants expressed a lack of stigmatisation.  Profession, years of experiences as a mental health professional, and occupational characteristics were not found to have a significant impact on overall stigmatisation. |
| Chikaodiri (2009) | Social workers  Unspecified medical doctors  Unspecified nurses  Pharmacists  Administrators  Laboratory scientists  Physiotherapists  Medical records officers  Hospital support staff  (Nigeria) | Cross-sectional survey | - | Mental illness in general (label) | Would not wish to have their place of work next door to the psychiatric wards  It is reasonable for the hospital staff to resist the location of psychiatric wards within the hospital | Most social workers did not express stigmatisation.  Other relevant findings were excluded from this table as they were not reported for social workers separately. |
| Cleary et al. (2002) | Psychiatrists  Psychologists  Social workers  Occupational therapists  Unspecified nurses from mental health and community health facilities  Other unspecified health professionals |  |  |  |  | Nothing more was reported for this study as findings were not reported for mental health professionals separately. |
| Clemente et al. (2017) | Psychiatrists  (Brazil) | Semi-structured interviews | Contextual semantic interpretation | Mental illness in general (label)  Bipolar disorder (label)  SUDs (label)  Schizophrenia (label) | Perceived insight  Perceived weakness  Perceived difficulty  Perceived unpredictability  Perceived uncontrollability  Perceived dangerousness to the self and others  Prognosis  Perceived aggression  People with mental illness are individuals who should be able to make their own decisions | Participants described people with bipolar disorder as lacking insight, weak, problematic, unpredictable, uncontrollable, and potentially dangerous to themselves and especially to their family and their own possessions.  Participants perceived people with bipolar disorder as unlikely to recover. Compared to schizophrenia however, the participants believed that people with bipolar disorder can have a good life.  Participants discussed aggressive behaviour when talking about mental disorders in general, and some comorbid conditions, such  as SUDs.  Participants also humanised people with mental illness, viewing them as individuals that should be able to make their own decisions regarding treatment. |
| Cohen & Struening (1962) | Psychiatrists  Social workers  Psychologists  Unspecified nurses that work in neuropsychiatric hospitals  Other unspecified physicians that work in neuropsychiatric hospitals  Psychiatry residents or trainees  Social work trainees  Psychology trainees  Nursing trainees  Dentists  Chaplains  Aides  Special services  Physiatrists  Kitchen workers  Clerks |  |  |  |  | Nothing more was reported for this study as findings were not reported for mental health professionals separately. |
| Cohen & Struening (1963) | Psychiatrists  Psychologists  Social workers  Unspecified nurses that work in mental hospitals  Other unspecified physicians that work in mental hospitals  Dentists  Dieticians  Lab technicians  Physiatrists  Clerks  Special services personnel  (USA) | Cross-sectional survey | Between-groups ANOVA  Cluster analysis | Mental illness in general (label) | OMI scale  Authoritarianism  Benevolence  Mental hygiene ideology  Social restrictiveness  Interpersonal aetiology  Profession | Overall, psychiatrists, psychologists, social workers, and nurses expressed more positive attitudes towards mental illness. However, for benevolence, psychologists either expressed roughly neutral responses, or more negative responses.  For interpersonal aetiology, psychiatrists, psychologists and social workers expressed more agreement. Nurses on the other hand expressed roughly neutral responses.  Profession was found to have a significant impact on all OMI scale factors. Although multiple comparisons were not applied to profession differences, clusters of professions were identified with respect to differences on the OMI scale.  Nurses formed a cluster with physicians and dentists, and dieticians, lab technicians, physiatrists, clerks, and special services personnel. This cluster was characterised by low authoritarianism but fairly neutral scores on benevolence, mental hygiene ideology, social restrictiveness, and interpersonal aetiology.  Psychologists and social workers formed a cluster. This cluster was characterised by low authoritarianism, low social restrictiveness, high mental hygiene ideology, high interpersonal aetiology, and neutral scores on benevolence.  Psychiatrists did not clearly fit into a particular cluster. They were characterised by low authoritarianism, low social restrictiveness, high benevolence, high mental hygiene ideology, and high interpersonal aetiology.  Other relevant findings were excluded from this table as they were not reported for other physicians separately. |
| Cohen & Struening (1964) | Psychiatrists  Social workers  Psychologists  Unspecified nurses that work in mental hospitals  Other unspecified physicians that work in mental hospitals and dentists  Nursing assistants  Physiatrists  Special services personnel |  |  |  |  | Nothing more was reported for this study as findings were not reported for mental health professionals separately. |
| Colombo et al. (2003) | Psychiatrists  Psychiatric nurses  Social workers  (England) | Structured interviews with open-ended questions  A vignette was used | - | Mental illness in general (label)  Schizophrenia (description) | Mental illness is a myth  Causal attributions  Prognosis generally and if changes are made at a societal level  Rights of the target (including blame)  Rights of society (including coercion)  Duties of society  Profession  Target attributes  Severity of learning problems  Levels of ego strength | None of the participants stated that mental illness is a myth (disagreement was not an available option).  Participants attributed schizophrenia to a range of causes with varying proportions. Social (e.g., marginal status) and biological causes were the most common explanations, and family-based causes were among the least common explanations.  Over half of the participants expressed a good prognosis for schizophrenia generally and just under half expressed a good prognosis for schizophrenia if changes are made at a societal level. Further, only a small proportion of participants expressed that therapy may be long term for the person in the vignette (disagreement was not an available option).  Half of the participants expressed that the person in the vignette had the right to the sick role, should be given sympathy, and should not be blamed. Most of the participants expressed that the person in the vignette had the right to privacy, personal freedom, and the same civil rights as anyone else. However, only a small proportion of participants expressed that the person in the vignette had the right to receive help and support as a victim of a stressful society (disagreement was not an available option).  Only a small proportion of participants expressed that society had the right to restrain/sanction those who break social rules, such as the person in the vignette. However, most participants expressed that society had the right to restrain those who are at risk of harming themselves or others, such as the person in the vignette. In comparison, less than half of the participants expressed that society had limited rights over the person in the vignette and should be proactive in preventing stress for this person (disagreement was not an available option).  Just over half of the participants expressed that society had the duty to empathise with and provide proper medical facilities for the care of the person in the vignette. Also, under half of the participants expressed that society had a duty to build therapeutic partnerships with, listen to and respect the views of people like the person in the vignette (disagreement was not an available option).  A small proportion of participants expressed that the prognosis of the mental disorder present in the vignette depends on severity of learning problems and levels of ego strength (disagreement was not an available option).  Some professions were more likely to attribute schizophrenia to particular causes. Psychiatrists were the most likely to attribute cause to biological and cognitive-behavioural factors (e.g., poor coping skills), and the they were the only participants to attribute cause to family interactions. Social workers were the most likely to attribute cause to early trauma, whereas none of the psychiatrists attributed cause to this factor. Nurses were the most likely to attribute cause to social factors.  No profession was consistently more or less likely to express a particular attitude across the stigmatisation measures.  Nurses were more likely than psychiatrists to express that prognosis for the mental disorder present in the vignette partly depended on the severity of learning problems. Further, social workers were the only participants to express that the prognosis of the mental disorder present in the vignette depends on levels of ego strength.  Differences between professions were not assessed with inferential statistics. |
| Corrigan et al. (2014) | Psychologists  Unspecified nurses from mental health and primary care clinics  Unspecified physicians from primary care clinics and other unspecified physicians from mental health clinics |  |  |  |  | Nothing more was reported for this study as findings were not reported for mental health professionals separately. |
| Cremonini et al. (2018) | Unspecified nurses from psychiatric care facilities  Social workers  Healthcare assistants  Educators  (Italy) | Cross-sectional survey | Between-groups ANOVA | Mental illness in general (label) | The authoritarianism, benevolence and social restrictiveness factors from the CAMI-I  Profession | Mental health professionals expressed more positive attitudes towards mental illness.  Profession was found to have a significant impact on attitudes. Social workers expressed more positive attitudes than nurses. However, these differences were not examined with multiple comparisons.  Other relevant findings were excluded from this table as they were not reported for mental health professionals separately. |
| Crowe & Averett (2015) | Psychologists  Social workers  Counsellors  (USA) | Cross-sectional survey with open-ended questions | Thematic analysis | Mental illness in general (label) | General attitudes  Empathy  Tolerance  Perceived strengths and abilities of people with mental illness  Perceived competence  Avoidance  Prognosis  Compassion  Frustration  People with mental illness can be insulting  Education in mental health  Professional experience  Religion  Media  Family  Personal familiarity with mental illness | Some participants suggested that their education in mental health made them more empathic and tolerant of people with mental illness, and more able to see the strengths and abilities of people with mental illness. However, some participants did not believe their education in mental health had any impact on their attitudes towards people with mental illness.  Some participants suggested that professional experience with people with mental illness made them more negative towards people with mental illness. This was exemplified by viewing people with mental illness as incapable and wanting to avoid people with mental illness. However, one participant believed that people with mental illness can function if they receive help and treatment. Some participants suggested that professional experience with people with mental illness has made them more able to see the strengths and abilities of people with mental illness. Also, some participants suggested that professional experience with people with mental illness had made them more compassionate, empathic, and tolerant of people with mental illness. However, these participants also believed that people with mental illness can be frustrating and insulting.  Participants also suggested that some other variables have an impact on their attitudes towards mental illness, but did not specify in what direction. These variables were religion, the media, family, and personal familiarity with mental illness. |
| Currin et al. (2009) | Primary care physicians  (England) | Cross-sectional survey  Vignettes were used | Paired samples t-test  Correlation analysis | Anorexia nervosa (label and description)  Bulimia nervosa (label and description) | A measure of attitudes towards eating disorders  This condition is psychological rather than medical  Patients with this condition are largely responsible for their own condition  Patients can do a lot to control these symptoms  This illness is likely to be chronic  This illness is a severe and enduring mental illness  Treatment is highly effective for patients with these symptoms  Symptoms of this illness are fairly common and will resolve over time, without specific treatment  This condition causes difficulties for a patient’s family and friends  This illness has major consequences on a patient’s quality of life  Compared to other patients I see in my practice, I generally enjoy working with these patients  Diagnosis  Knowledge of eating disorders | Participants agreed more with a psychological rather than medical aetiology of both mental disorders, and disagreed more that the targets are largely responsible for their condition.  Participants agreed more that the targets could do a lot to control their symptoms. However, participants also agreed more that both mental disorders are enduring and likely to be chronic. Whether participants agreed or disagreed more with the other prognosis items was dependent on the eating disorder (e.g., participants agreed more that treatment is highly effective for bulimia nervosa, but disagreed more for anorexia nervosa).  Participants agreed more that both mental disorders cause difficulties for their family and friends.  An overview of the other items in the measure of attitudes towards eating disorders was not reported in this table as those items are not relevant to stigmatisation.  In comparison to anorexia nervosa, participants agreed more with a psychological aetiology for bulimia nervosa, and disagreed less that the target with bulimia nervosa was responsible for their condition. For every other relevant item, anorexia nervosa was stigmatised more than bulimia nervosa. Compared to bulimia nervosa, participants agreed significantly more that anorexia nervosa is likely to be chronic, and cause difficulties for family and friends. This was the only difference between the eating disorders that was examined with inferential statistics. However, participants were asked to make a diagnosis for the vignettes (this was all that was stated), and it was found that the diagnoses were not found to be significantly related to overall attitude scores.  Knowledge was not found to be significantly correlated with attitudes. |
| Dabby et al. (2015) | Psychiatrists  Psychiatry residents  (Canada) | Cross-sectional survey  IAT  Short descriptions were used | - | Mental illness in general (label)  Schizophrenia (description)  Hallucination, delusion, psychosis, paranoia (labels) | Social distance towards schizophrenia  OMS-HC (factors were not specified)  Implicit attitudes towards schizophrenia (e.g., delusion)  Positive (i.e., joy, love, peace, wonderful, pleasure, glorious, laughter, happy)  Negative (i.e., agony, terrible, horrible, nasty, evil, awful, failure, hurt) | Psychiatrists displayed little evidence of stigmatisation on all measures.  Other relevant findings were excluded from this table as they were not reported for psychiatrists separately. |
| Daibes et al. (2017) | Unspecified nurses from addiction rehabilitation facilities  (Jordan) | Semi-structured interviews | Thematic analysis | Drug and alcohol addiction (labels)  Other unspecified conditions (label) | Perceived and endorsed causal attributions/blame  Addicts and alcoholics are very bad people  Addicts and alcoholics are monsters  Addicts and alcoholics are barbarians  Addicts and alcoholics are criminals  Addicts and alcoholics are careless  Addicts and alcoholics are irresponsible  Addicts and alcoholics are undisciplined  Addicts and alcoholics are out of control  Addicts and alcoholics are sexually deviant  Addicts and alcoholics are liars  Addicts and alcoholics are pretenders  Addicts and alcoholics are cheaters  Fear  Lack of trust  Neglectful of the subjective experience of patients  Avoidance  Sympathy  Prognosis  Intention to provide the same level of care as other conditions  Substance addiction is shameful and wrong  Comfort coming into contact with patients with substance addiction  Curiosity for how the patient became addicted  Socio-economic status of the patient  Sex of the patient  Age of the patient  Whether the patient has been abandoned by their family  Mass media  Socialisation  Type of drug being used by patients | Participants described addicts and alcoholics as very bad people, monsters, barbarians, criminals, careless, irresponsible, undisciplined, out of control, sexually deviant, to blame for their condition, liars, pretenders, and cheaters.  One participant stated they were fearful of patients with addiction because they are sexually deviant.  It was suggested that due to the lack of trust participants had in patients with substance addiction, participants would neglect the subjective experience of patients with addiction and focus more on objective means to verify complaints.  Participants would avoid patients with addiction. Participants stated that the only reason they would talk to patients with addiction was out of curiosity for how the patient became addicted. It was suggested that participants would avoid patients because they are seen as criminals and out of control.  Participants were more inclined to stigmatise patients with addiction if they came from a high socio-economic background. Participants were more likely to blame patients with addiction if they came from high socio-economic backgrounds, and sympathise with patients that came from low-socio-economic backgrounds.  Participants believed that people with substance addiction are unlikely to recover, and one participant stated for that reason they should not receive the same level of care as other conditions.  Participants stated that substance addiction is shameful and wrong.  Compared to males, participants blamed females more for their substance addiction and attributed it more to sexual deviation. This was also perceived in other nurses.  Participants were less stigmatising of younger patients.  It was suggested that stigmatisation was exacerbated if a patient was abandoned by their family.  Participants reported that mass media and socialisation contribute to their stigmatisation of substance addiction.  It was suggested that participants were uncomfortable coming into contact with patients that had a substance addiction because the participants were worried they would become addicted.  Participants stigmatised patients that were addicted to prescribed drugs less than patients that were addicted to alcohol and illegal drugs. |
| Dale & Middleton (1990) | GPs  (England) | Cross-sectional survey  Vignettes were used | Exploratory factor analysis  Correlation analysis | Multiple unspecified mental disorders (descriptions)  Physical illness in general (descriptions) | Enthusiasm towards the target in the vignette  Sympathy towards the target in the vignette  Irritation towards the target in the vignette  Anxiety towards the target in the vignette  How appropriate a consultation would be with the target in the vignette  How urgent a consultation would be with the target in the vignette  Professional experience with mental illness  Age  Whether participants worked in a non-training practice or not  Number of postgraduate qualifications  Sex | The amount of stigmatisation present in this study was not reported.  For mental illness, enthusiasm, sympathy, lack of irritation, and deeming a consultation appropriate for the person in the vignette formed a factor. There was a significant positive correlation between this factor and more professional experience with mental illness.  For both mental and physical illness, enthusiasm, sympathy, and lack of irritation formed a factor. This factor was significantly positively associated with more professional experience with mental illness, older age, and working in a non-training practice. For both mental and physical illness, irritation and deeming it appropriate to have a consultation with the person in the vignette formed another factor. However, this factor was significantly positively associated with more professional experience with mental illness, older age, and fewer postgraduate qualifications.  For both mental and physical illness, anxiety towards the target in the vignette and deeming it urgent to have a consultation with the person in the vignette formed a factor. This factor was significantly positively associated with being female. |
| Dalky et al. (2020) | Unspecified physicians from primary healthcare centres  (Jordan) | Cross-sectional survey | Correlation analysis | Mental illness in general (label) | RIBS  Are you currently living with, or have you ever lived with, someone with a mental health problem?  Are you currently working with, or have you ever worked with, someone with a mental health problem?  Do you currently have, or have you ever had, a neighbour with a mental health problem?  Do you currently have, or have you ever had, a close friend with a mental health problem?  In the future, I would be willing to live with someone with a mental health problem  In the future, I would be willing to work with someone with a mental health problem  In the future, I would be willing to live nearby to someone with a mental health problem  In the future, I would be willing to continue a relationship with a friend who developed a mental health problem  Age | Participants expressed more negativity on the RIBS.  Age was not found to be significantly correlated with scores on the RIBS. |
| Day et al. (2018) | Mental health nurses  (Australia) | Longitudinal survey and semi-structured interviews | Thematic analysis  Correlation analysis  Independent samples t-test  Mann-Whitney *U*-test | Personality disorder (label)  BPD (label) | Prognosis  Perceived difficulty  General dislike  Perceived manipulativeness, attention seeking, and superficiality  Frustration  Empathy  APDQ (only items relevant to stigmatisation were included in this table)  Protective  Fondness  Interested  Unable to gain control  Frightened  Helpless  Intolerant  Angry  Frustrated  Drained  ADSHQ  Confidence in assessment and referral  Ability to deal effectively with clients (subjective)  Empathic approach  Ability to cope with legal and hospital regulations that guide practice  ASQ (only items relevant to stigmatisation were included in this table)  Willingness in working with BPD  Optimism in working with BPD  Age  Years of professional experience  Cohort  Year 2000  Year 2015  Level of training  Undergraduate  Postgraduate  Exposure to BPD specific training | Participants from both cohorts expressed the view that BPD is chronic and people with BPD are difficult. However, participants from the 2000 cohort also expressed general dislike towards BPD, and the view that people with BPD are manipulative, attention seeking and superficial. Participants from the 2015 cohort expressed the view that people with BPD are frustrating, but also expressed empathy towards people with BPD.  Participants expressed that they feel protective and fond of personality disorder between occasionally and very often. Participants also expressed that they feel the last six APDQ factors between occasionally and very often. An overview of the interested and unable to gain control factors of the APDQ was not included in this table as those factors are not relevant to stigmatisation.  Participants expressed more willingness and optimism in working with BPD.  The subjective ability of participants to deal with self-harming clients was significantly positively correlated with more positive overall attitudes on the APDQ. Whether any of the other ADSHQ factors were found to be significantly correlated with APDQ scores was not reported.  Age and years of professional experience were not found to be significantly correlated with overall APDQ scores.  Compared to participants from the 2000 cohort, participants from the 2015 cohort were significantly more protective, fond and tolerant of personality disorder. Cohort was not found to have a significant impact on any of the other relevant APDQ items.  Level of training was not found to have a significant impact on overall APDQ scores.  Exposure to BPD specific training was not found to have a significant impact on overall APDQ scores. This was only assessed for the 2015 cohort. |
| Deans & Meocevic (2014) | Psychiatric nurses  (Australia) | Cross-sectional survey | - | BPD (label) | A measure of attitudes towards people with BPD (only items that are relevant to stigmatisation were included in this table)  People with BPD are manipulative  People with BPD emotionally blackmail people they work with  People with BPD are nuisances  People with BPD are time wasters  People with BPD make me angry  People with BPD are charming | Half or most participants expressed stigmatisation for some items, and less than half expressed stigmatisation for other items (a neutral option was available). |
| Deans & Soar (2005) | Unspecified nurses from a psychiatric facility  A social worker  A psychiatrist  A psychologist  GPs (only discussed by the other professionals)  (Australia) | Unstructured interviews | Interpretative phenomenological analysis | Mental illness and a coexisting SUD (label)  Schizophrenia (label)  Other unspecified clients (label) | Frustration  Prognosis  Ease of treatment (this was not too clear)  Perceived empathy experienced by GPs  Judgmental attitudes in general  Sympathy  Causal attributions  Perceived difficulty  People with comorbid mental illness and SUD are not motivated in their treatment  Powerlessness and helplessness  Perceived risk of violence and contracting hepatitis when visiting people with comorbid mental illness and SUD at home | Participants expressed frustration towards comorbid mental illness and SUD. It was suggested that this was due to the chronic nature of this condition.  Participants stated that it was easier to treat a person with schizophrenia compared to comorbid mental illness and SUD. This was again attributed to the chronic nature of comorbid mental illness and SUD.  Younger compared to older GPs were perceived as more empathic of comorbid mental illness and SUD. Participants also suggested that GPs from small/one-doctor towns tend to express judgemental attitudes towards people with comorbid mental illness and SUD.  Some participants expressed sympathy towards comorbid mental illness and SUD.  One participant suggested that comorbid mental illness and SUD occurs when a person with mental illness uses substances to cope.  Comorbid mental illness and SUD was perceived as the most challenging and difficult of all clients. Another participant also perceived comorbid mental illness and SUD to be difficult.  Participants felt powerless and helpless because they believed that people with comorbid mental illness and SUD are not motivated in their treatment. One participant also felt powerless due to the chronic nature of comorbid mental illness and SUD.  Participants perceived a risk of violence when visiting a person with comorbid mental illness and SUD. One participant also perceived a risk of contracting hepatitis when visiting a person with comorbid mental illness and SUD. |
| Deehan et al. (1997) | GPs  (England) | Cross-sectional survey | t-test for two dependent proportions | Drug misusers (label)  Alcohol misusers (label)  Other unspecified patients (label) | Perceived difficulty | Most participants perceived both disorders to take up more time than other patients and to present major management problems (uncertain was an available option).  Drug misusers were significantly more likely to be perceived as taking up more time than other patients and presenting major management problems. |
| Delaruelle et al. (2021) | GPs  Candidate GPs |  |  |  |  | Nothing more was reported for this study as findings were not reported for GPs separately. |
| Dell et al. (2021) | Social workers  Psychologists  Counsellors  Unspecific nurses from community mental health centres  Other unspecified service providers from community mental health centres |  |  |  |  | Nothing more was reported for this study as findings were not reported for mental health professionals separately. |
| Del Olmo-Romero et al. (2019) | Psychiatrists  Psychologists  Unspecified nurses from mental health institutions  Social workers  Occupational therapists  Rehabilitation technicians  Social educators  Other unspecified mental health professionals  Assistant nurses  Administrative and general service staff  Other unspecified non-clinical professionals  (Spain, Italy, Portugal) | Cross-sectional survey  Vignettes were used | Between-groups ANOVA  Multiple regression analysis | Mental illness in general (label)  Schizophrenia (description and a possible label) | AQ-27 (items were not specified)  Perceived personal responsibility  Pity  Anger  Perceived dangerousness  Fear  Helping  Coercion  Segregation  Avoidance  CAMI questionnaire  Authoritarianism  One of the main causes of mental illness is a lack of self-discipline and will power  The best way to handle the mentally ill is to keep them behind locked doors  There is something about the mentally ill that makes it easy to tell them from normal people  As soon as a person shows signs of mental disturbance, he should be hospitalized  Mental patients need the same kind of control and discipline as a young child  Mental illness is an illness like any other  The mentally ill should not be treated as outcasts of society  Less emphasis should be placed on protecting the public from the mentally ill  Mental hospitals are an outdated means of treating the mentally ill  Virtually anyone can become mentally ill  Benevolence  The mentally ill have for too long been the subject of ridicule  More tax money should be spent on the care and treatment of the mentally ill  We need to adopt a far more tolerant attitude toward the mentally ill in our society  Our mental hospitals seem more like prisons than like places where the mentally ill can be cared for  We have a responsibility to provide the best possible care for the mentally ill  The mentally ill don't deserve our sympathy  The mentally ill are a burden on society  Increased spending on mental health services is a waste of tax dollars  There are sufficient existing services for the mentally ill  It is best to avoid anyone who has mental problems  Social restrictiveness  The mentally ill should not be given any responsibility  The mentally ill should be isolated from the rest of the community  A woman would be foolish to marry a man who has suffered from mental illness, even though he seems fully recovered  I would not want to live next door to someone who has been mentally ill  Anyone with a history of mental problems should be excluded from taking public office  The mentally ill should not be denied their individual rights  Mental patients should be encouraged to assume the responsibilities of normal life  No one has the right to exclude the mentally ill from their neighbourhood  The mentally ill are far less of a danger than most people suppose  Most women who were once patients in a mental hospital can be trusted as babysitters  Community mental hygiene ideology  Profession | Across the stigmatisation factors, psychiatrists, psychologists, nurses, and other unspecified mental health professionals mostly expressed positive attitudes. However, these groups also expressed more negative attitudes for coercion and avoidance. The only exception to this was, psychologists responded roughly neutrally to the avoidance factor.  Profession was found to have a significant impact on all of the stigmatisation factors. Across the different factors, there were no consistent differences between psychiatrists, psychologists, nurses, and other unspecified mental health professionals. All of these groups stigmatised more than the others at least once and stigmatised less than the others at least once. Differences between these groups were not examined with multiple comparisons.  Other relevant findings were excluded from this table as they were not reported for mental health professionals separately. |
| Des Courtis et al. (2008) | Psychiatrists  Psychologists  Social workers  Unspecified nurses from mental health facilities and a general hospital  Vocational workers  Physiotherapists  Other unspecified health professionals |  |  |  |  | Nothing more was reported for this study as findings were not reported for mental health professionals separately. |
| Deska et al. (2020) | Faculty in clinical and counselling PhD programs actively seeing clients or supervising clinical cases  Graduate student clinicians  Undergraduate students |  |  |  |  | Nothing more was reported for this study as findings were not reported for faculty members separately. |
| Dowrick et al. (2000) | GPs  (England) | Cross-sectional survey | Correlation analysis | Depression (label) | DAQ (only factors relevant to stigmatisation were included in this table)  Treatment attitude  Inevitable course of depression/pessimism about depression  Diagnostic competence  Identification (ability to detect depression)  Accuracy (ability to make assessments that are congruent with patient symptom levels)  Bias (tendency to either make diagnoses or avoid making diagnoses)  Prescription of antidepressant medication | Participants agreed slightly less with treatment attitudes, suggesting slightly more disagreement with attributing depression to poor stamina/a biochemical abnormality. Participants also disagreed more with inevitable course of depression/pessimism about depression.  There was a significant negative correlation between treatment attitude and diagnostic accuracy. A significant correlation was not found between treatment attitude and the other two diagnostic competences. However, inevitable course of depression/pessimism about depression was significantly negatively correlated with diagnostic accuracy and identification, but was not found to be significantly correlated with bias.  Treatment attitude was significantly positively associated with prescribing SSRIs, but was not found to be significantly correlated with prescribing tricyclic antidepressants. Inevitable course of depression/pessimism about depression was not found to be significantly correlated with the prescription of antidepressants. |
| Drake et al. (2018) | SUD treatment providers and trainees from the following fields  Social work  Counselling  Psychology  Nursing  Sociology  Other unspecified fields |  |  |  |  | Nothing more was reported for this study as findings were not reported for mental health professionals separately. |
