# Supplementary material for: The stigmatization of mental illness by mental health professionals: Scoping review and bibliometric analysis
Source: PLoS One. 2023 Jan 20;18(1):e0280739. doi: 10.1371/journal.pone.0280739 (PMC9858369; doi:10.1371/journal.pone.0280739)
Supplement: S4 Appendix — (DOCX) [file pone.0280739.s004.docx]

| **Authors (year)** | **Populations**  **(countries)** | **Research methods** | **Analytical approaches** | **Disorders** | **Variables and measures** | **Findings** |
| --- | --- | --- | --- | --- | --- | --- |
| Eack & Newhill (2008) | Social workers  (USA) | Cross-sectional survey | Correlation analysis | Mental illness in general (label) | A measure of attitudes towards mental illness  Mental illness is not a preferred population to treat  Not optimistic about treatment  People with mental illness fail to follow through on treatment  Mental illness is intellectually challenging to understand  Clinicians prefer to avoid contact with mental illness  Special countertransference problems (this was not clear)  People with mental illness are difficult to relate to  There are no satisfying rewards working with mental illness  Clinicians do not receive adequate training  Burnout is common when working with mental illness  Frustrations with the following  Medication noncompliance  Legal leverage to force treatment  Bizarre behaviour  Manipulative behaviour  A person not accepting their illness  Difficulty obtaining resources  Managed care regulations  Uncooperative caregivers  Lack of client improvement  Threatening/violent client behaviour  Difficulty with colleagues  Waiting lists for services | The level of stigmatisation present in this study was not reported.  The view that mental illness is not a preferred population to treat was significantly positively correlated with special countertransference problems and the preceding items for the measure of attitudes towards mental illness. This item was not found to be significantly correlated with mental illness is intellectually challenging to understand. Not being optimistic about treatment was significantly positively correlated with believing people with mental illness fail to follow through on treatment and the preceding items for the measure of attitudes towards mental illness. Believing people with mental illness fail to follow through on treatment was significantly positively correlated with mental illness is intellectually challenging to understand and the preceding items for the measure of attitudes towards mental illness.  The view that mental illness is not a preferred population to treat was significantly positively correlated with the first six frustrations. This item was also significantly positively correlated with frustration at a lack of client improvement. This item was not found to be significantly correlated with any of the other frustrations. Not being optimistic about treatment was significantly positively correlated with frustrations at legal leverage to force treatment, bizarre behaviour, a person not accepting their illness, lack of client improvement, and threatening/violent client behaviour. This item was not found to be significantly correlated with any of the other frustrations. Believing that people with mental illness fail to follow through on treatment was significantly positively correlated with most of the frustrations. However, this item was not found to be significantly correlated with frustration towards managed care regulations and threatening/violent client behaviour. |
| Ebrahimi et al. (2017) | Psychiatric nurses  Unspecified non-psychiatric nurses  (Iran) | Cross-sectional survey | The analysis was unclear | Mental illness in general (label) | CAMI questionnaire  Authoritarianism  Benevolence  Social restrictiveness  Community mental health ideology  Willingness to continue working in a psychiatric ward  Amount of professional experience in a psychiatry ward | Psychiatric nurses expressed more positive attitudes for authoritarianism and social restrictiveness, but more negative attitudes for benevolence and community mental health ideology.  Willingness to continue working in a psychiatric ward and amount of professional experience in a psychiatry ward were not found to have a significant impact on stigmatisation. Inferential statistics for this were not reported.  Other relevant findings were excluded from this table as they were not reported for psychiatric nurses separately. |
| Ebrahimi et al. (2012) | Unspecified nurses from psychiatric wards  (Iran) | Cross-sectional survey | Correlation analysis  Mann-Whitney *U*-test  Kruskal-Wallis test | Mental illness in general (label) | A measure of stigmatisation towards mental illness (items were not specified)  Cognitive component  Emotional component  Behavioural component  Age  Sex  Marital status  Married  Single  Working shift schedule  Morning fixed  Nights fixed  Changing  Employment type  Plan  Contract  Formal  Working in the ward according to personal interest  Satisfaction with working in the ward  Level of training  University of graduation  Governmental  Azad  Interest in continuation of work in the ward  Personal familiarity and lived experience with mental illness | Most participants expressed a medium level of stigmatisation for the cognitive component, and a low level of stigmatisation for the emotional component. Roughly half of the participants expressed a high level of stigmatisation for the behavioural component, and just under half expressed a medium level of stigmatisation.  Age was not found to be significantly correlated with stigmatisation.  Sex, marital status, working shift schedule, employment type, and working in the ward according to personal interest were not found to have a significant impact on stigmatisation.  Satisfaction with working in the ward was not found to be significantly correlated with stigmatisation.  Participants with a bachelor’s or master’s degree stereotyped mental illness significantly less than participants with a diploma. No other significant differences were reported with respect to level of training, and no other comparisons were made.  The university participants graduated from and interest in continuation of work in the ward had a significant impact on emotions. However, no other details were reported.  Participants with personal familiarity and lived experience with mental illness expressed significantly less negative emotions and discrimination. No other significant differences were reported with respect to personal familiarity and lived experience with mental illness. |
| Economou et al. (2020) | Psychologists  Social workers  Psychiatrists  Psychiatric nurses  (Greece) | Cross-sectional survey | Independent samples t-test  Correlation analysis  Between-groups ANOVA  Multiple regression analysis | Mental illness in general (label) | ASMI scale  If a person has experienced severe mental illness, he/she will suffer from it for the rest of his/her life  People with severe mental illness have to take medication for as long as they live  People with severe mental illness can recover nowadays  People with severe mental illness are failures  No matter how hard they try, people with severe mental illness will never be like other people  Severe mental illness makes the person who suffer from it look ill from a distance  Once ill, people with severe mental illness stop being like other people  It is easy for other people to recognize that someone has severe mental illness  People with severe mental illness cannot acquire new skills  People with severe mental illness are dangerous  Severe mental illness is responsible for all the misfortunes of a person  All psychiatric medication cause addiction  A person with severe mental illness is able to work  A person with severe mental illness can receive training for an occupation  People with severe mental illness do not differ from other people  People with severe mental illness can cope with life difficulties  Taking psychiatric medication does not render a person with severe mental illness different from other people  People with severe mental illness should not give up  People with severe mental illness should seek help from a mental health professional  It is better for a person with severe mental illness to hang out only with people who also have a mental disorder  It is better for people with severe mental illness to conceal their illness, so as to avoid life difficulties  Friends should not avoid a person with severe mental illness when he/she falls ill  It is better for a person with severe mental illness to avoid other people  People with severe mental illness should not hide their problem from family and friends  People with severe mental illness usually feel a burden to their families  People with severe mental illness usually feel inferior to other people  People treat differently a person with severe mental illness when he/she falls ill  People blame a person with severe mental illness for every misfortune occurs to his/her family  People with severe mental illness usually feel responsible for their illness  It is difficult for other people to understand how a person with severe mental illness feels  GSD scale  Decide to live in house building, where someone with mental illness also resides  Feel afraid to have a conversation with someone with mental illness  Be upset or disturbed about working on the same job with someone with mental illness  Feel upset or disturbed about rooming with someone with mental illness  Feel ashamed if people knew someone in your family has mental illness  Feel annoyed or disturbed about sitting next to someone with mental illness in the bus  Maintain a friendship with someone with mental illness  Marry someone with mental illness  Lend anything of yours to someone with mental illness  Accept a person with mental illness as your hairdresser  Rent your house to someone with mental illness  Hire someone with mental illness  Decide to live in neighbourhood, where an institution for the treatment of people with mental illness is operating  Start a friendship with a person with mental illness  Age  Family status  Single  Married/cohabiting  Divorced/widowed  Years with tenure  Duration of work experience  Profession (focal and control variable)  Personal experience with mental illness  Lived experience  Relative  Close friend (focal and control variable)  Acquaintance  Colleague (focal and control variable)  Income  Education (levels for this were not made clear)  Gender  The IRI was used to measure trait empathy  Perspective taking (one’s tendency to spontaneously adopt the psychological perspective of another person)  Fantasy (one’s ability to place oneself into the shoes of fictional characters in literature and movies)  Empathic concern (other-oriented feelings of sympathy and concern over misfortunes)  Personal distress (self-oriented feelings of anxiety and unease during intense interpersonal encounters) | For the ASMI and GSD scales the majority of participants expressed positivity for most of the items relevant to stigmatisation. However, roughly half of the participants expressed positivity for the first three items in the ASMI scale. Also, for the GSD scale most participants expressed negativity towards the prospect of marrying someone with mental illness, roughly half felt upset or disturbed about rooming with a person with mental illness, and roughly half expressed positivity at living in a neighbourhood with a psychiatric institution.  Age, family status, years with tenure, and duration of work experience were not found to be significantly correlated with or to have a significant impact on overall scores on either of the stigmatisation measures.  Compared to nurses, being a psychologist or social worker was a significant predictor of more overall positive responses on both stigmatisation measures. Being a psychiatrist was also a significant predictor of more overall positive responses on the ASMI scale compared to nurses. However, the overall scores for these two professions were not found to be significantly different for the GSD scale. Stigmatisation scores were not compared between psychologists, social workers and psychiatrists.  For the ASMI scale, lived experience with mental illness and having an acquaintance with mental illness were not found to have a significant impact on overall scores. Also, having a close friend or relative with mental illness were not found to be significant predictors of overall scores on the ASMI scale. However, having a colleague with a mental illness was a significant predictor of more overall positive responses on the ASMI scale. For the GSD scale, none of the forms of experience with mental illness, except for having a close friend with mental illness, were found to have a significant impact on overall scores. Having a close friend with mental illness was found to be a significant predictor of more overall positive responses on the GSD scale  Income, education, and gender were not found to be significantly correlated with or to have a significant impact on overall scores on the ASMI scale. Also, these variables were not found to be significant predictors of overall scores on the GSD scale.  Controlling for the significant predictors in the previous analyses (with the respective measures), perspective taking was found to be significant predictor of more overall positive responses on both measures of stigmatisation. Also, fantasy was found to be a significant predictor of overall scores on the GSD scale, but was not found to be a significant predictor of overall scores on the ASMI scale. Still controlling for the significant predictors in the previous analyses, the other two factors of the IRI were not found to be significant predictors of overall scores for either measure of stigmatisation. |
| Egan et al. (2014) | Clinical psychologists  (Australia) | Cross-sectional survey | Hierarchical regression analysis | Personality disorder (label) | APDQ  Enjoyment  Security  Acceptance  Purpose  Enthusiasm  Professional familiarity with personality disorder (i.e., what percentage of clients do you have that are diagnosed with a personality disorder)  Sex  Age  Recency of specialist training on personality disorder | Participants expressed more overall positive attitudes.  More professional familiarity with personality disorder was a significant predictor of positive attitudes. The other variables were not found to be significant predictors of attitudes towards personality disorder. |
| Egbe et al. (2014) | A psychiatric nurse  Auxiliary social workers  A range of other nurses  Lay counsellors |  |  |  |  | Nothing more was reported for this study as findings were not reported for psychiatric nurses separately. |
| Eker (1985) | Psychologists  Psychiatrists  Social workers  (Turkey, USA) | Cross-sectional survey | Between-groups ANOVA | Mental illness in general (label) | Attitudes were measured with the following semantic differentials  Weak/strong  Bad/good  Irresponsible/responsible  Foolish/wise  Voluntary/involuntary (it was not clear what this meant)  High education/low education  Strange/familiar  Predictable/unpredictable  Clean/dirty  Dangerous/safe  Slow/fast  Cold/warm  Psychology/biology (it was not clear what this meant)  Sociology/psychology (it was not clear what this meant)  Retarded/intelligent  Sick/healthy  Relaxed/tense  Country | Participants endorsed involuntary more than voluntary, and psychology more than biology and sociology.  For most of the other semantic differentials participants expressed more negative attitudes. The only exceptions to this were, Turkish participants endorsed wise more than foolish, and responded neutrally to slow/fast, and USA participants endorsed intelligent slightly more than retarded.  The only significant difference between Turkish participants and USA participants regarded the foolish/wise semantic differential. No other significant differences were found. |
| Elwy et al. (2013) | Unspecified physicians from a primary care practice  Resident physicians from a primary care practice |  |  |  |  | Nothing more was reported for this study as findings were not reported for primary care physicians separately. |
| Farmer & Greenwood (2009) | GPs  (England) | Cross-sectional survey and structured interviews | - | Problem drinkers (label) | Pessimism is the most realistic attitude to take towards drinkers  In general, I like drinkers  Causal attributions  Problem drinkers can be identified based on their appearance/they are unkempt  Frustration  Perceived difficulty | Just over half the participants disagreed that pessimism is the most realistic attitude to take towards drinkers. However, only a small proportion of participants agreed to liking drinkers (neutral responses were available).  Almost half of the participants agreed that alcohol misuse is a symptom of an underlying personality disorder, and under half agreed that alcohol misuse is self-inflicted (neutral responses were available).  A small proportion of participants believed that problem drinkers can be identified based on their appearance/they are unkempt.  Under half of the participants believed that problem drinkers are frustrating and difficult to treat. |
| Finamore et al. (2020) | Unspecified mental health professionals  (England) | Longitudinal survey  An intervention was used | Paired samples t-test | Personality disorder (label) | A measure of negative emotional reactions (this includes feelings of being manipulated and feeling overwhelmed, but specific items were not specified) | Before an intervention that addresses misconceptions about personality disorder, the participants expressed slightly more overall stigmatisation. After the intervention participants expressed slightly less overall stigmatisation, and time point was found to have a significant impact on overall stigmatisation. |
| Fitzgerald & McNicholas (2014) | Psychologists  Psychiatrists  Paediatricians  Unspecified primary care professionals |  |  |  |  | Nothing more was reported for this study as findings were not reported for mental health professionals separately. |
| Flanagan et al. (2016) | Unspecified mental health professionals  Unspecified primary care professionals  Unspecified obstetric and gynaecological professionals  A pharmacist  Other unspecified health professionals |  |  |  |  | Nothing more was reported for this study as findings were not reported for mental health professionals separately. |
| Flanagan et al. (2009) | Unspecified mental health professionals  (USA) | Structured and semi-structured interviews | Interpretive phenomenological analysis | Mental illness in general (label) | Causal attributions  People with mental illness have a lot of potential  General attitudes  General emotional reactions  Positive attributes in general  Perceived ability of people with mental illness to cope  People with mental illness are strong and courageous  Empathy  Happiness at the success of people with mental illness  Sadness  Perceived self-destructiveness  Perceived incompetence  People with mental illness are weak and have only him/herself to blame  People with mental illness are brain damaged  People with mental illness are drowsy  People with mental illness are emotional  People with mental illness are able to recover  People with mental illness have thinking problems  People with mental illness have attention problems  People with mental illness are different  People with mental illness are insightful  People with mental illness are confused  People with mental illness are unstable  People with mental illness ramble on  People with mental illness are unpredictable  People with mental illness are excessively loud or soft  People with mental illness are emotionally insightful  People with mental illness are no different  People with mental illness have a dishevelled appearance  People with mental illness are shameful  People with mental illness are obsessed  People with mental illness are aggressive  People with mental illness are normal  People with mental illness are scary  People with mental illness are difficult to talk to  People with mental illness are strange looking  People with mental illness are apathetic  People with mental illness are dirty  People with mental illness are likely to be violent  People with mental illness are incoherent  People with mental illness are smelly  People with mental illness have intelligence problems  People with mental illness have consciousness problems  People with mental illness stutter  People with mental illness are mentally retarded | One participant alluded to mental illness being caused by extraordinary life experiences coupled with difficulty in coping with such experiences. This participant also believed that people with mental illness have a lot of potential.  Some participants reported general positive perceptions and emotional reactions towards mental illness. Others however, reported general negative perceptions and emotional reactions towards mental illness, and some reported a mixture of both positive and negative reactions.  One participant highlighted the strengths of people with mental illness in general, and their ability to cope with their lives. Relatedly, one participant described people with mental illness as strong and courageous. Another participant felt privileged to be able to work with people with mental illness. A fourth participant described feeling empathy towards people with mental illness and happiness at their success. However, this participant also described feeling sad at the self-destructive behaviours of people with mental illness.  One participant described people with mental illness as incompetent.  Participants disagreed more that people with mental illness are weak, only have themselves to blame, and are brain damaged.  For the remaining measures, participants expressed more stigmatisation for some measures, less stigmatisation for some measures, and roughly neutral responses for other measures. |
| Foster & Onyeukwu (2003) | Forensic psychiatric nurses  (England) | Cross-sectional survey | Bivariate regression analysis | Substance abuse (label) | SAAS (items were not specified by the authors and only factors relevant to stigmatisation were included in this table)  Treatment optimism (an optimistic perception of  treatment and the possibility of a successful outcome)  Non-moralism (absence/avoidance of moralistic perspective when considering substance use and substance users)  Non-stereotyping (non-reliance on popular societal  stereotypes of substance use and substance users)  Sex  Nursing grade  Staff nurses  Other grades (i.e., ward managers, charge nurses, senior staff nurses, enrolled nurses)  Ethnicity  Black  Non-Black  Age  40 and below  41 and above  Level of training  Non-graduate  Undergraduate/postgraduate  Years of experience  Less than four years  Four years and more | Overall, participants expressed more stigmatisation.  Being male was a significant predictor of more stigmatisation on the non-moralism factor. No other significant findings were reported for sex.  Being a staff nurse was a significant predictor of less stigmatisation on the non-stereotyping factor. No other significant findings were reported for nursing grade.  Being non-Black was a significant predictor of more stigmatisation on the treatment optimism factor. No other significant findings were reported for ethnicity.  No other variables were found to be significant predictors of stigmatisation. |
| Foster et al. (2008) | Unspecified nurses from a psychiatric hospital  Medical orderlies  (Fiji) | Cross-sectional survey | - | Mental illness in general (label)  Alcohol abuser (label) | ATAMHS 33 (only items that were relevant to stigmatisation and summarised for nurses separately were included in this table)  Alcohol abusers have no self control  Mentally ill patients have no control over their emotions  Mental illness is the result of adverse social circumstances  Many normal people would become mentally ill if they had to live in a very stressful situation  Mental illnesses are genetic in origin  Those with a psychiatric history should never be given a job with responsibility  Psychiatric illness deserves as much attention as physical illness  Psychiatric drugs are used to control disruptive behaviour | Most nurses agreed that people who abuse alcohol have no self control. However, most nurses disagreed that mentally ill patients have no control over their emotions. Most nurses agreed that mental illness is the result of negative social circumstances, and many normal people would become mentally ill if they had to live in a very stressful situation. Despite this, most nurses also agreed the mental illnesses are caused by genetic factors.  Most nurses disagreed that those with a psychiatric history should never be given a job with responsibility, and most nurses agreed that psychiatric illness deserves as much attention as physical illness. Most nurses also agreed that psychiatric drugs are used to control disruptive behaviour.  Other relevant findings were excluded from this table as they were not reported for nurses separately. |
| Franz et al. (2021) | Primary care/family physicians  Internal medicine physicians  Emergency medicine physicians |  |  |  |  | Nothing more was reported for this study as findings were not reported for primary care/family physicians separately. |
| Fraser & Gallop (1993) | Unspecified nurses from psychiatric units  (Canada) | Behavioural observation  Actual patient interactions were used | Between-groups ANOVA | Schizophrenia (label and presentation)  BPD (label and presentation)  Affective disorder (label and presentation)  Other unspecified mental disorders (label and presentation) | Self-reported emotional responses  Helpless  Frustrated  Angry  Caring  Confused  Confirming/disconfirming behavioural responses (these were not made very clear)  Confirming  Disparagement  Inadequate  Ambiguous  Impervious  Indifferent  Tangential | Descriptive statistics were not reported for level of stigmatisation.  Participants expressed significantly more overall positive emotions towards schizophrenia and affective disorder compared to BPD. Further, participants expressed significantly more overall negative emotions towards BPD compared to schizophrenia and affective disorder. Other unspecified mental disorders were not compared to the other mental disorders, and no other significant differences were reported between the mental disorders for emotional responses.  Participants displayed significantly more disconfirming responses towards BPD than affective disorder and other unspecified mental disorders. No significant difference was found between BPD and schizophrenia. No other significant differences were reported between the mental disorders for disconfirming behavioural responses. |
| Fuss et al. (2018) | Psychologists  Psychiatrists  Other unspecified mental health professionals  (Germany, Austria, Switzerland) | Experiment  Vignettes were used | Between-groups ANOVA | Psychosis (description)  Exhibitionism (description)  Frotteurism (description)  Sexual sadism (description)  Paedophilia (description)  Sexual masochism (description) | Causal attributions  Blame  Social distance  Perceived dangerousness  Target sex | Psychosis was attributed to biological and psychological causes equally, whereas the other mental disorders were attributed to psychological causes more.  Participants desired social distance from and blamed all the mental disorders more. Participants perceived psychosis and sexual masochism as less dangerous, and sexual sadism and paedophilia as more dangerous. Female cases of exhibitionism were perceived as less dangerous, and male cases elicited roughly neutral responses. Female cases of frotteurism were perceived as less dangerous, and male cases were perceived as more dangerous.  Psychosis was attributed to biological causes significantly more than all other mental disorders, and paedophilia was attributed psychological causes significantly less than the other paraphilic disorders. No significant differences were found between the other paraphilic disorders for causal attributions.  For each measure of stigmatisation, a hierarchical pattern emerged, in which participants stigmatised the different mental disorders to varying degrees. This pattern was different for each measure of stigmatisation. Also, different patterns emerged depending on target sex. For a male target, paedophilia was consistently stigmatised more than exhibitionism, sexual masochism and psychosis across the measures of stigmatisation. Similarly, sexual sadism was stigmatised consistently more than frotteurism, sexual masochism and psychosis. Also, exhibitionism and frotteurism were stigmatised consistently more than sexual masochism and psychosis. For a male target, the only consistent difference found was paedophilia and sexual sadism were consistently stigmatised more than all the other disorders. No other consistent differences between mental disorders were found across the measures of stigmatisation. None of these differences between mental disorders were examined with inferential statistics. However, the mental disorders were compared with inferential statistics using an aggregate of blame, social distance and perceived dangerousness scores. In this analysis, psychosis was stigmatised significantly less than all other mental disorders, followed by sexual masochism, followed by exhibitionism, followed by frotteurism. Sexual sadism and paedophilia were not found to be significantly different.  Compared to a female target, a male target elicited significantly more social distance and perceived dangerousness for most of the mental disorders. The only exception to this was target sex was not found to have a significant impact on social distance and perceived dangerousness for sexual masochism. A male target with frotteurism was blamed significantly more than a female target with frotteurism. Target sex was not found to have a significant impact on blame for any of the other mental disorders. |
