# Supplementary material for: The stigmatization of mental illness by mental health professionals: Scoping review and bibliometric analysis
Source: PLoS One. 2023 Jan 20;18(1):e0280739. doi: 10.1371/journal.pone.0280739 (PMC9858369; doi:10.1371/journal.pone.0280739)
Supplement: S5 Appendix — (DOCX) [file pone.0280739.s005.docx]

| **Authors (year)** | **Populations**  **(countries)** | **Research methods** | **Analytical approaches** | **Disorders** | **Variables and measures** | **Findings** |
| --- | --- | --- | --- | --- | --- | --- |
| Gallop et al. (1989) | Unspecified nurses from psychiatric settings  (Canada) | Experiment  Vignettes were used | Binomial test for paired comparisons  Chi-square test of independence | BPD (description and label)  Schizophrenia (description and label) | QMEE (only behavioural categories relevant to stigmatisation were included in this table)  Belittles or contradicts patient  Expresses care or concern  Age (levels were not specified)  Years of professional experience (levels were not specified)  Education  Hospital nursing school  Community colleges  Universities | A small proportion of the participants expressed care or concern towards the targets. However, a smaller proportion belittled or contradicted the targets.  BPD was significantly more likely to elicit belittling or contradiction than schizophrenia. No significant difference was found between the mental disorders regarding the proportion of participants expressing care or concern.  There was a significant relationship between age and belittling or contradicting. The youngest age group belittled or contradicted less than the other age groups. Age was not found to have a significant relationship with belittling or contradicting.  Years of professional experience and education were not found to have a significant relationship with either of the QMEE behavioural categories. |
| Gateshill et al. (2011) | Psychiatrists  Mental health nurses  Social workers  GPs  Junior and middle-grade hospital doctors  Primary and secondary care nurses |  |  |  |  | Nothing more was reported for this study as findings were not reported for mental health professionals separately. |
| Gilchrist et al. (2011) | Psychiatrists  Psychologists  Social workers  Psychiatric nurses  General nurses working in general psychiatry and from specialist addiction services  GPs/first contact physicians  Unspecified physicians from primary care centres  Unspecified physicians working in general psychiatry  Unspecified physicians working in specialist addiction services  Unspecified nurses from primary care centres  Unspecified physicians from internal medicine emergency departments  (Bulgaria, Greece, Italy, Poland, Scotland, Slovakia, Slovenia, Spain) | Cross-sectional survey and structured interviews | Between-groups ANOVA | Problems related to alcohol (label)  Problems related to drugs (label)  Depression (label) | MCRS  Profession  Treatment service entry point  Primary care General psychiatry  Specialist addiction | Psychiatrists, psychologists, and social workers expressed more positive regard to all the mental disorders.  For psychiatrists, psychologists, and social workers, problems related to drugs elicited the least positive regard, followed by problems related to alcohol, followed by depression. This however was not assessed with inferential statistics for mental health professionals separately.  Profession was found to have a significant impact on regard for all of the mental disorders. For the most part, psychologists expressed more positive regard than social workers, and social workers expressed more positive regard than psychiatrists. The only exception to this was psychiatrists expressed slightly more positive regard towards depression than psychologists, and psychologists expressed more positive regard than social workers. It was found that psychologists expressed significantly more positive regard for problems related to both alcohol and drugs compared to psychiatrists. All other profession differences were either not clearly examined with multiple comparisons, or were not examined with multiple comparisons separately for mental health professionals.  For psychiatrists, psychologists, and social workers treatment entry point was found to have a significant impact on regard for problems related to alcohol and drugs (depression was not included in this analysis). In both cases, psychiatrists, psychologists, and social workers from specialist addiction expressed the most positive regard, followed by general psychiatry, followed by primary care. These differences were not clearly examined with inferential statistics.  Other relevant findings were excluded from this table as they were not reported for mental health professionals separately. |
| Gonzales (2021) | Clinical psychologists  Psychology students  Medical students |  |  |  |  | Nothing more was reported for this study as findings were not clinical psychologists separately. |
| Gonzales et al. (2021) | Clinical psychologists  Trainee psychologists  Medical students  General population  (USA) | Cross-sectional survey  Vignettes were used | - | Schizophrenia (label and description)  Mental illness in general (label) | AQ-27 (most items were not specified)  Familiarity with mental illness  Perceived personal responsibility  Pity  Anger  Fear  How scared of Harry would you feel?  Helping  How likely is it that you would help Harry?  Coercion-segregation  If I were in charge of Harry’s treatment, I would require him to take his medication  RIBS  A 14-item version of the MIMS-P was used to measure microaggressions (most items were not specified)  If I saw a person who I thought had a mental illness in public, I would be careful in case they snap  If someone I’m close to told me that they had a mental illness diagnosis, I would expect them to have trouble understanding some things | Clinical psychologists expressed more overall positivity on the AQ-27 towards the target with schizophrenia. They also expressed more overall positivity towards mental illness on the RIBS, and slightly more overall positivity on the MIMS-P.  Other relevant findings were excluded from this table as they were not reported for clinical psychologists separately. |
| Gove et al. (2016) | GPs  (England) | Semi-structured telephone interviews | Thematic analysis | Dementia (label) | Dementia is pitiful  Dementia is characterised by being curled up in a deformed position, crying, screaming, and being unable to have any kind of meaningful interaction  Sadness  Stage of dementia  People with dementia are incontinent  People with dementia have become non-persons and have lost the essence if their being  Frustration and irritation  Empathy  Sympathy  Concern  Protectiveness  Dementia is unjust  People with dementia are unable to give back to society | One GP described the advanced stages of dementia as pitiful, and characterised by being curled up in a deformed position, crying, screaming, and being unable to have any kind of meaningful interaction. This GP reported feeling sad thinking about this.  GPs stereotyped people with dementia as being incontinent. Again however, this was seen as occurring only in the advanced stages of dementia.  One GP referred to people with advanced dementia as becoming a non-person, and another stated that people with dementia have lost the essence of their being.  Some GPs found people with dementia to be frustrating and irritating. However, they mostly felt empathy, sympathy, concern, protectiveness, sadness, and a sense that dementia is unjust.  GPs expressed that people with dementia are unable to give back to society. |
| Graham et al. (2010) | Psychologists  Social workers  Occupational therapists  Counsellors  Speech pathologists  Managers  Unspecified nurses  Personal carers/aged care workers  Welfare workers  Youth workers/youth health workers  Those who work in a school environment  Housing support workers  Psychiatric disability rehabilitation and support service workers  Community workers  Women’s health/support workers  Case managers  Disability workers  Aboriginal health workers  Physiotherapists  Childcare/child welfare workers  Carer support workers  Administrators  Dieticians  Volunteers  Family support workers  Health promotion workers  Employment consultants  Police officers  Pastoral workers  Outreach workers  Other unspecified professionals |  |  |  |  | Nothing more was reported for this study as findings were not reported for mental health professionals separately. |
| Grausgruber et al. (2007) | Psychiatric nurses  Social workers  Occupational therapists  Psychologists  Physiotherapists |  |  |  |  | Nothing more was reported for this study as findings were not reported for mental health professionals separately. |
| Guise et al. (2010) | Psychiatric nurses  General nurses working in a mental healthcare organisation  Other unspecified non-psychiatric nurses working in a mental healthcare organisation  (England) | Cross-sectional survey | MANOVA | Mental illness in general (label) | CAMI questionnaire  Authoritarianism  Benevolence  Social restrictiveness  Community mental health ideology  Mode of data collection  Web-based  Paper-based | Participants displayed less stigmatisation across all the factors.  Mode of data collection was not found to have a significant impact on any of the CAMI factors. |
| Gupta et al. (1992) | GPs  (India) | Cross-sectional survey | - | Mental illness in general (label) | Casual attributions  Prognosis in general and if left alone  Social distance  Do people around mental illness tend to become odd or strange themselves? | Most participants believed that mental disorders are inherited genetically, can occur in a normal person under stress, and can be caused by an abnormal family or society. Most participants also believed that mental illness is an illness. Roughly half of the participants believed that mental illness can be caused by poor living conditions, and no participants believed that mental disorders are due to God’s punishment for some sin or wrongdoing.  Most participants did not believe that mental illness is untreatable. However, most participants did not believe that mental illness can improve if a patient is left alone and nothing is done.  About half of the participants expressed social distance towards mental illness.  Most participants did not believe that people around mental illness tend to become odd or strange themselves. |
| Gutierrez & Ruiz (1978) | Psychiatric nurses  (Spain) | Cross-sectional survey | - | Mental illness in general (label) | Causal attributions  Semantic differentials  Wise-foolish  Intelligent-ignorant  Sincere-insincere  Warm-cold  Clean-dirty  Good-bad  Beautiful-ugly  Valuable-worthless  Safe-dangerous  Familiar-strange  Healthy-sick  Active-passive  Fast-slow  Strong-weak  Understandable-mysterious  Relaxed-tense  Predictable-unpredictable  Prognosis  Mental patients have a strange expression in their eyes  Practically all mental patients are capable of attacking others without reason  Young women must always be particularly careful of mental patients  Mental patients should not be allowed to look after small children  There is something about mental patients which make them easily distinguishable from normal people  Mental patients should not be allowed to get married  I should hesitate to employ a mental patient | Most participants attributed mental illness to disturbed personality development. A small proportion of participants attributed mental illness to chemical brain disorders and heredity, and an even smaller proportion attributed mental illness to civilisation. No participants attributed mental illness to financial difficulties and drug and alcohol abuse, and a small proportion of participants did not respond.  For some of the semantic differentials, participants expressed more stigmatisation, whereas for other semantic differentials participants expressed more positive attitudes. Positive attitudes were more common than negative attitudes.  About half of the participants believed that mental illness requires long ambulatory treatment. Less than half of the participants believed that mental illness can be cured in a short time by a psychiatrist. Roughly the same amount of participants believed that mental illness is usually recurrent and needs frequent long-term hospitalisation. No participants believed that mental illness is incurable and needs permanent hospitalisation, and can be cured usually without a psychiatrist. A small proportion of participants did not respond.  For the remaining measures, either half or most of the participants expressed agreement with some of the measures, and either less than half or a small proportion expressed agreement for other measures. |
| Haddad et al. (2015) | Mental health nurses  GPs  Counsellors  Psychological therapists  A psychiatrist  Practice nurses |  |  |  |  | Nothing more was reported for this study as findings were not reported for mental health professionals separately. |
| Hamdan-Mansour & Wardam (2009) | Mental health nurses  (Jordan) | Cross-sectional survey | Mann-Whitney *U*-test  Chi-square test of independence  Correlation analysis | Mental illness in general (label)  Alcohol abuser (label)  Chronic schizophrenia (label)  Depression (label) | ATAMHS 33  Care or control  Semantic differentials  Therapeutic perspective  Hard to help  Positive attitudes  Age  Sex  Special training in psychiatric nursing  Place of work  Private  Governmental  Marital status  Married  Single  Level of training  Masters  Bachelor  Associate  Satisfaction with nursing care at the current organisation | Most participants agreed that alcohol abusers have no self control, and depression occurs in people with a weak personality. Most participants also agreed that mentally ill patients have no control over their emotions, and disagreed that many normal people would become mentally ill if they had to live in a very stressful situation, and people are born vulnerable to mental illness. However, most participants agreed that mental illness is the result of adverse social circumstances, and mental illnesses are genetic in origin.  Most participants disagreed that psychiatric illness deserves as much attention as physical illness, and agreed that patients with chronic schizophrenia are incapable of looking after themselves and it is hard to help patients who are emotionally disturbed. Most participants also agreed that psychiatric drugs are used to control disruptive behaviour, and people with mental illness are dangerous, immature, cold-hearted, harmful, pessimistic, and have poor hygiene. In contrast, only half of the participants believed that people with mental illness are rude and childlike.  The remaining items in the ATAMHS 33 were either not included in this table due to their irrelevance to stigmatisation, or were not summarised by the authors.  Age was found to have a significant impact on ATAMHS 33 scores. In particular, age was found to have a significant impact on the adult-child and caring-cold-hearted semantic differentials. This is all that was reported for the effect of age.  Females expressed more overall positive attitudes on the ATAMHS 33 compared to males. This overall difference was not examined with inferential statistics. However, there was a significant relationship between sex and the item patients with chronic schizophrenia are incapable of looking after themselves, and the polite-rude semantic differential. This was all that was reported for the former item. Regarding the latter, females were more likely to perceive people with mental illness as polite compared to males. No significant relationship was found between sex and the item those with a psychiatric history should never be given a job with responsibility. Nothing else was reported for the relationship between sex and the relevant items.  Having special training in psychiatric nursing was not found to be significantly related to the item mental illnesses are genetic in origin. This variable was significantly related to the items members of society are at risk from the mentally ill, and acute wards are little more than prisons. However, nothing else was reported for these relationships, and the relationship between special training in psychiatric nursing and the relevant items in general.  Place of work had a significant relationship with the items acute wards are little more than prisons, mental illnesses are genetic in origin, and the caring-cold-hearted semantic differential. Nothing else was reported for these relationships, and the relationship between place of work and the relevant items in general.  Marital status had a significant relationship with the item patients with mental illnesses are more likely to harm someone else than themselves. However, nothing else was reported for these relationships, and the relationship between marital status and the relevant items in general.  Level of training had a significant relationship with the items violence mostly results from mental illness, and psychiatric illness deserves as much attention as physical illness. Nothing else was reported for these relationships, and the relationship between level of training and the relevant items in general.  There was a significant negative correlation between satisfaction and the item patients with mental illnesses are more likely to harm someone else than themselves. There was also a significant positive correlation between satisfaction and perceiving people with mental illness as rude and childlike. Nothing else was reported for the relationship between satisfaction and the relevant items. |
| Harris et al. (2016) | Psychiatrists  Psychologists  Social workers  Unspecified nurses  Other unspecified health professionals |  |  |  |  | Nothing more was reported for this study as findings were not reported for mental health professionals separately. |
| Hartmann (1989) | Family physicians  (USA) | Quasi-experiment  An intervention was used | Independent samples t-test | Somatoform disorder (label) | Anger  Anxiety  Resentment  Perception that people with somatoform disorder take up too much time | The intervention used was a series of Balint groups using cognitive therapeutic approaches that focused on somatoform disorder.  Before the intervention, participants expressed a moderate level of anger and less anxiety, but more resentment and more of a perception that people with somatoform disorder take up too much time. In comparison, participants in the control group at baseline expressed less anger, anxiety, and resentment, and neutral responses to people with somatoform disorder taking up too much time.  Participants perceived that people with somatoform disorder take up too much time significantly less at postintervention, compared to preintervention. This was the only significant difference found between pre and postintervention. No significant differences were found between baseline and follow up scores for the control group. |
| Hayes et al. (2004) | Counsellors  (USA) | Experiment  Interventions were used | Mixed ANOVA  Correlation analysis | Drug addicts and alcoholics (label) | CASA (this is a modified version of the CAMI questionnaire that changes mental illness to drug addicts and alcoholics)  Authoritarianism (one unspecified item was removed from this factor)  Benevolence  Social restrictiveness  Community mental health ideology  Another measure of attitudes towards drug addicts and alcoholics (only the following examples were provided)  My client is not going to change no matter what I do  If my clients really wanted to get sober, they would  Burnout | Participants were randomly assigned to either training in acceptance and commitment therapy, multicultural training, or training on the role of methamphetamines and related chemicals in addiction.  Prior to the interventions, participants expressed more overall positive attitudes on both attitude measures.  There was a significant time point by intervention interaction effect for overall scores on the CASA.  For participants receiving acceptance and commitment therapy training, overall stigmatisation on the CASA decreased significantly from pre-intervention to follow-up. However, no significant difference was found between pre-intervention and post-intervention. For multicultural training, overall stigmatisation on the CASA decreased significantly from pre-intervention to post-intervention, but no significant difference was found between pre-intervention and follow up. For methamphetamine training, time point was not found to have a significant impact on overall CASA scores.  A significant time point by intervention interaction effect was not found for overall scores on the other measure of attitudes. For participants receiving acceptance and commitment therapy training, overall stigmatisation decreased significantly from pre-intervention to post-intervention, and pre-intervention to follow-up. For multicultural training, overall stigmatisation decreased significantly from pre-intervention to post-intervention, but no significant difference was found between pre-intervention and follow up. For methamphetamine training, time point was not found to have a significant impact on overall CASA scores.  For participants receiving acceptance and commitment therapy training, there was a significant positive correlation between stigmatisation on the other measure of attitudes, and burnout. A significant relationship was not found between these two variables for participants in the multicultural training group. The relationship between these two variables was not examined for the methamphetamine training group. |
| Hayes & Wall (1998) | Clinical psychologists  Other unspecified mental health professionals  (USA) | Experiment  Vignettes were used | Factorial ANOVA  Multiple regression analysis | PTSD (description)  Bulimia (description) | Perceived responsibility of cause  Perceived responsibility of problem solution  Level of responsibility attributed by the target  None  Low  High  Theoretical orientation  Psychodynamic  Humanistic  Leaning  Other | Participants attributed less responsibility to the targets for the cause of their problem. PTSD was attributed less responsibility for a solution, and bulimia was attributed more responsibility for a solution. However, when the target with bulimia attributed a high level of responsibility to them self for solving the problem, participants responded roughly neutrally for problem solution.  Participants attributed significantly more responsibility of cause and problem solution to the bulimia target than to the PTSD target.  Level of responsibility attributed by the target was not found to have a significant impact on perceived responsibility of cause or problem solution.  Psychodynamic orientation was a significant predictor of less perceived responsibility of cause. However, the reference group was not made clear. None of the other theoretical orientations were found to be significant predictors of perceived responsibility of cause. Again, the reference group was not stated. Theoretical orientation was not found to be a significant predictor of perceived responsibility of problem solution. |
| Heinz et al. (2019) | Counsellors  (Germany) | Cross-sectional survey | Correlation analysis  Fisher-Freeman-Halton test | Depression (label) | The personal stigma subscale of the DSS  People with depression could snap out of it if they wanted  Depression is a sign of personal weakness  Depression is not a real medical illness  People with depression are dangerous  It is best to avoid people with depression so you don’t become depressed yourself  People with depression are unpredictable  If I had depression I would not tell anyone  I would not employ someone if they knew they had a been depressed  I would not vote for a politician if I knew they had been depressed  Number of years working at current organisation  Age  Self-rated knowledge about depression and suicide  When do you pose concrete questions about suicidality?  If I get suspicious  Only if the caller mentions suicidality himself  If proof becomes more and more evident throughout the conversation  Reported management of callers at risk of suicide  Didn’t go into detail  Asked for reasons  Informed about specific contacts  Advice of seeking help immediately  Called ambulance/police  Others  I don’t know | Overall, participants expressed a lack of stigmatisation.  Number of years working at the current organisation was significantly correlated with less overall stigmatisation.  Older age was significantly correlated with less overall stigmatisation.  High self-rated knowledge about depression and suicide was significantly correlated with less overall stigmatisation.  Participants with a higher overall stigmatisation score were significantly more likely to pose concrete questions only if the caller mentions suicidality himself, compared to participants with lower overall stigmatisation scores. However, participants with a higher overall stigmatisation score were significantly less likely to pose concrete questions if they get suspicious, compared to compared to participants with lower overall stigmatisation scores. Also, participants with a higher overall stigmatisation score were slightly more likely to pose concrete questions if proof becomes more and more evident, compared to participants with lower overall stigmatisation scores. Whether this difference was statistically significant was not reported.  Reported management of callers at risk of suicide was not found to have a significant impact on overall stigmatisation. |
| Hengartner et al. (2012) | Psychiatrists  Psychologists  Social workers  Other unspecified physicians/therapists working in psychiatric institutions  Other unspecified physicians/therapists  Aides |  |  |  |  | Nothing more was reported for this study as findings were not reported for mental health professionals separately. |
| Heresco-Levy et al. (1999) | Psychiatrists  Psychologists  Social workers  Occupational therapists  Psychiatric nurses  Nurse assistants |  |  |  |  | Nothing more was reported for this study as findings were not reported for mental health professionals separately. |
| Holleman et al. (2000) | Primary care physicians  (USA) | Cross-sectional survey | Independent samples t-test  Structural equation modelling | Substance abuse (label) | A measure of negative attributions (only one example item was provided)  You can't win when treating substance-abuse patients  Sex  A measure of trait authoritarianism (only one example item was provided)  Those who contribute the  most to society should get better health care  Depressed mood  A measure of clinical uncertainty tolerance (only one example item was provided)  I do not enjoy treating patients whose illness is unlikely to respond to treatment  Excessive reliance on technological aspects of medicine (e.g., for me, the laboratory profile is the most important part of the medical record) | Participants expressed overall negative attributions regarding substance abuse.  Sex was not found to have a significant impact on overall negative attributions.  Higher authoritarianism, depressed mood, and intolerance of clinical uncertainty were found to be significant predictors of more overall negative attributions.  Higher reliance on the technological aspects of medicine was a significant predictor of less overall negative attributions. |
| Holmqvist (2000) | Unspecified nurses working in psychiatric units  Social workers  Psychologists  Psychiatric aides |  |  |  |  | Nothing more was reported for this study as findings were not reported for mental health professionals separately. |
| Holmqvist & Armelius (2004) | Unspecified nurses working in psychiatric units  Social workers  Psychologists  Psychiatric aides |  |  |  |  | Nothing more was reported for this study as findings were not reported for mental health professionals separately. |
| Hori et al. (2011) | Psychiatrists  Other unspecified physicians  Unspecified nurses  Pharmacologists  A community health worker  General population  (Japan) | Cross-sectional survey | - | Schizophrenia (label) | Patients with schizophrenia can work  Would oppose if one of his/her relatives would like to marry someone who has schizophrenia  Schizophrenia patients can be recognized by his/her appearance  Schizophrenia patients are dangerous  Would not like to have a neighbor with schizophrenia  Schizophrenia patients are untrustworthy  Schizophrenia patients could harm children  Schizophrenia patients should be kept in hospitals  I don't worry about examining a person who is diagnosed with schizophrenia  Schizophrenia can be treated  Patients with schizophrenia cannot comprehend their illness  Patients with schizophrenia cannot comprehend nor apply suggested treatment  Schizophrenia has the chance of recovery | Most psychiatrists responded positively to the measures. The only exception to this was most psychiatrists agreed that they would oppose if one of his/her relatives would like to marry someone who has schizophrenia.  Other relevant findings were excluded from this table as they were not reported for mental health professionals separately. |
| Howard & Holmshaw (2010) | Psychiatrists  Unspecified nurses from mental health services  Occupational therapists/assistants  Ward managers/team leaders  Health care assistants/support, time and recovery workers  Environment coordinators  Welfare rights workers  Senior house officers  Other unspecified staff |  |  |  |  | Nothing more was reported for this study as findings were not reported for mental health professionals separately. |
| Hsiao et al. (2015) | Mental health nurses  (Taiwan) | Cross-sectional survey  Short descriptions were used (the substance abuse description was not clear) | Repeated-measures ANOVA  Correlation analysis  Hierarchical regression analysis  Independent samples t-test | Substance abuse (description and possible label)  Schizophrenia (description and label)  Major depression (description and label) | AMIQ  Do you think that this would damage John’s career?  I would be comfortable if John was my colleague at  work?  I would be comfortable about inviting John to a dinner party  How likely do you think it would be for John’s wife to leave him?  How likely do you think it would be for John to get in trouble with the law?  Sex  Age  Years of mental health nursing experience  Seniority  Head nurses/supervisors  Staff nurse  Work setting  Acute psychiatric inpatient units  Psychiatric rehabilitation units  Outpatient clinics/community psychiatric rehabilitation centres  Attitudes towards empathy in patient care | Participants expressed more negative attitudes overall on the AMIQ towards the mental disorders.  Substance abuse elicited the most overall negative attitudes on the AMIQ, followed by schizophrenia, followed by major depression. Type of mental disorder was found to have a significant impact on AMIQ overall scores. However, differences between the mental disorders were not examined with multiple comparisons.  Sex was not found to have a significant impact overall AMIQ scores for any of the mental disorders.  Age was significantly positively associated with overall positive attitudes towards all the mental disorders on the AMIQ.  Having more years of experience in mental health nursing was a significant predictor of more overall positive attitudes on the AMIQ for all mental disorders.  Seniority was not found to be a significant predictor of overall attitudes on the AMIQ for any of the mental disorders.  Compared to working in psychiatric rehabilitation units, working in the other two settings were not found to be significant predictors of overall attitudes on the AMIQ for any of the mental disorders. In this analysis, these two settings were not compared. However, in a bivariate analysis, participants from acute psychiatric inpatient units expressed significantly more overall negative attitudes on the AMIQ towards substance abuse and schizophrenia, compared to participants from outpatient clinics/community psychiatric rehabilitation centres. A significant difference between these two groups was not found for major depression.  Having a more positive attitude towards empathy in patient care was a significant predictor of more overall positive attitudes towards all of the mental disorders on the AMIQ. |
| Hugo (2001) | Mental health nurses  Psychiatrists  Social workers  Clinical psychologists  Occupational therapists  Trainee psychiatrists  Unspecified medical officers from psychiatric facilities  Activity supervisors  (Australia) | Cross-sectional survey  Vignettes were used | Independent samples t-test | Schizophrenia (description)  Depression (description) | Prognosis with and without professional help  Full recovery with no further problems  Full recovery, but problems would probably recur  Partial recovery  Partial recovery, but problems would probably recur  No improvement  Get worse  Perceived long-term outcomes  Negative outcomes  Be violent  Drink too much  Take illegal drugs  Have poor friendships  Positive outcomes  Understand other’s feelings  Have a good marriage  Be a caring parent  Be a productive worker  Be creative or artistic | For both mental disorders, and with professional help, full recovery, but with problems likely recurring was the most common prognosis by nurses. For depression, the next most likely prognosis was full recovery with no further problems. However, for schizophrenia partial recovery, but problems likely recurring was the next most common prognosis. The remaining prognoses with professional help were selected by either no nurses or a small proportion of nurses for both mental disorders. Prognoses were much more likely to be poor if the targets were not receiving professional help. This was not examined with inferential statistics.  For depression, nurses mostly expressed a lack of stigmatisation. The only exception to this was two positive outcomes were perceived as less likely. For schizophrenia, nurses expressed more stigmatisation. However, one negative outcome was perceived as less likely, one positive outcome was perceived as more likely, and two negative outcomes were believed to be just as likely compared to the general population.  Nurses were overall significantly more optimistic about the prognosis of depression compared to schizophrenia. However, without professional help participants were more likely to predict that depression would partially recover, but with problems likely recurring, and not improve. This was not assessed with inferential statistics.  Nurses rated positive outcomes as more likely for depression than schizophrenia, and negative outcomes as less likely for depression than schizophrenia. The only exception to this was, schizophrenia was perceived as more likely than depression to be creative or artistic. These differences were not clearly examined with inferential statistics.  Other relevant findings were excluded from this table as they were not reported for mental health professionals separately. |
