# Supplementary material for: The stigmatization of mental illness by mental health professionals: Scoping review and bibliometric analysis
Source: PLoS One. 2023 Jan 20;18(1):e0280739. doi: 10.1371/journal.pone.0280739 (PMC9858369; doi:10.1371/journal.pone.0280739)
Supplement: S6 Appendix — (DOCX) [file pone.0280739.s006.docx]

| **Authors (year)** | **Populations**  **(countries)** | **Research methods** | **Analytical approaches** | **Disorders** | **Variables and measures** | **Findings** |
| --- | --- | --- | --- | --- | --- | --- |
| Ighodaro et al. (2015) | Primary care physicians  (Nigeria) | Cross-sectional survey | - | Mental illness in general (label)  Schizophrenia (label)  Major depression (label)  Bipolar disorder (label)  Anxiety disorder (label) | A measure of attitudes towards mental illness  Socializing  Not upset or disturbed about working on the same job  Willing to work with somebody with a mental illness  People with mental illness can work in regular jobs  People with mental illness are not a public nuisance  Do not object to having mentally ill people in my neighborhood  Not unwilling to share a room  Not afraid to have a conversation  Would invite somebody with mental illness into my home  Not afraid of people with mental problems living in residential neighborhoods  I am not afraid of people with mental illness  Are not dangerous because of violent behavior  Should have the same rights to a job as anyone else  Do not end to mentally retarded  Do not mind living next door to someone who has been mentally ill  Not ashamed if someone in your family had been diagnosed with a mental illness  Non-superstitious about witchcraft  Disagree that witchcraft can cause mental illness  Disagree that possession by evil spirits can cause mental illness  Disagree that a curse can cause mental illness  Disagree that God’s punishment can cause mental illness  Neighborly feelings  Would have casual conversations with neighbors with mental illness  Would have a former psychiatric patient as a friend  Would occasionally greet a former patient that came to live next door to you  Stress and abuse etiology  Stress can cause mental illness  Physical abuse can cause mental illness  Poverty can cause mental illness  Perceived effectiveness of treatment for the particular mental disorders | Most participants expressed positive attitudes on the measure of attitudes towards mental illness.  Participants believed that all the mental disorders would improve with treatment. The following mental disorders are listed from least likely to improve with treatment to most likely to improve.  Schizophrenia  Bipolar disorder  Major depression  Anxiety disorder  These differences were not examined with inferential statistics. |
| Imran & Haider (2007) | Family physicians  Unspecified house officers  Medical students |  |  |  |  | Nothing more was reported for this study as findings were not reported for family physicians separately. |
| Ishige & Hayashi (2005) | Psychiatric nurses  Public health nurses  Health care assistants  Medical case workers  Social welfare workers  Local welfare commissioners  Professional probation officers  Other unspecified non-psychiatric care workers  Unspecified non-care workers  (Japan) | Cross-sectional survey | - | Schizophrenia (label) | Semantic differentials  Safe vs harmful  Bad vs good  Annoying vs pleasing  Dark vs bright  Miserable vs merry  Wonderful vs fearful  Fierce vs gentle  Warm vs cool  Repellant vs attractive  Social distance | Psychiatric nurses stigmatised schizophrenia less overall for the semantic differentials, and expressed slightly more social distance towards schizophrenia.  Other relevant findings were excluded from this table as they were not reported for psychiatric nurses separately. |
| James & Cowman (2007) | Psychiatric nurses  (Ireland) | Cross-sectional survey | - | BPD (label)  Other unspecified clients (label)  Other unspecified psychiatric disorders (label) | Prognosis  Perceived difficulty | Half of the participants expressed that a number of people with BPD achieve some stability in their 40s (I don’t know was an available option).  Most of the participants agreed that clients with BPD are very or moderately difficult to look after.  Most of the participants agreed that clients with BPD are more difficult to look after than other clients and more difficult to care for than those with other psychiatric disorders. |
| James et al. (2012) | GPs  (Nigeria) | Cross-sectional survey | - | Depression (label) | DAQ (only items relevant to stigmatisation were included in this table)  Becoming depressed is a way that people with poor stamina deal with life’s difficulties  Becoming depressed is a natural part of old age  It is possible to distinguish two main groups of depression: one psychological in origin and the other caused by biochemical abnormalities  The majority of depression seen in general practice originates from patients’ recent misfortunes  Depressed patients are more likely to have experienced deprivation early in their lives compared to other people  An underlying biochemical abnormality forms the basis of severe cases of depression  Working with depressed patients can be difficult  Most depressive disorders seen in general practice improve without medication  Depression reflects a characteristic response that is not amenable to change | For the majority of the causal attribution items, most participants expressed agreement. However, roughly half the participants expressed agreement for the item becoming depressed is a way that people with poor stamina deal with life’s difficulties, and most participants disagreed that becoming depressed is a natural part of old age.  Most participants agreed that working with depressed patients can be difficult.  Less than half of the participants agreed that most depressive disorders seen in general practice improve without medication and most participants disagreed that depression reflects a characteristic response that is not amenable to change. |
| Jones et al. (2009) | Psychiatrists  Primary care physicians  (USA) | Structured interviews  Vignettes were used | Chi-square test of independence  Mann-Whitney *U*-test  One of the analyses was not clear | Mental illness in general (label)  Schizophrenia (descriptions and label) | Mental illness interferes with history taking and physical examination  Other stereotypes  Dangerous  Unpredictable  Unhealthy  Not intelligent  Unreliable  Difficult  Uncontrollable  Unkempt  Tiresome  Abnormal  Non-adherent to medications  A measure of attitudes  Level of discomfort dealing with the target  Prematurely ending a visit with the target  Perceived negative impact of the target on examination  Type of schizophrenia symptoms  Positive  Negative  Profession | Most participants believed that mental illness interferes with history taking sometimes, and a small proportion believed that this always occurs. Also, a small proportion of primary care physicians believed that this never occurs. Most psychiatrists believed that mental illness interferes with physical examination sometimes, a small proportion believed that this never occurs, and a small proportion believed this this always occurs. For primary care physicians, roughly half believed that mental illness interferes with physical examination sometimes, roughly half believed that this never occurs, and a small proportion believed that this always occurs.  Participants stereotyped schizophrenia more overall.  Overall participants expressed more negative attitudes towards schizophrenia on the measure of attitudes.  A significant relationship was not found between profession and the view that mental illness interferes with history taking. However, there was a significant relationship between profession and the perception that mental illness interferes with physical examination. Primary care physicians were more likely to believe that this never occurs and less likely to believe that it occurs sometimes. Profession was not found to have a significant impact on overall stereotyping of schizophrenia, and attitudes towards schizophrenia on the measure of attitudes.  For psychiatrists, there was no difference between positive and negative symptoms for the measure of attitudes. Primary care physicians expressed more negative attitudes towards positive symptoms compared to negative symptoms. However, this difference was not found to be statistically significant. |
| Jones et al. (2013) | Psychiatrists  (England) | Cross-sectional survey | Mann-Whitney *U*-test | Anorexia nervosa (label)  Bulimia nervosa (label) | Causal attributions  The mental disorders represent abnormal behaviour in the context of a weak, manipulative or inadequate personality  The mental disorders are essentially untreatable  It is appropriate that the MHA enables compulsory re-feeding of patients with anorexia nervosa  The MHA should not be used to enforce admission to hospital for patients with anorexia nervosa  The MHA should not be used when patients clearly believe that the advantages of anorexia nervosa outweigh the disadvantages  The MHA should be used more frequently to protect the health and safety of patients with anorexia nervosa  Grade  Consultants  Non-consultants  Seniority  Psychiatrists of higher seniority  Junior psychiatrists | Roughly half of the participants believed that the mental disorders are culturally determined by women’s role in society, and less than half believed that the mental disorders are a neurophysiological disorder of unknown origin.  Very few participants believed that the mental disorders represent abnormal behaviour in the context of a weak, manipulative or inadequate personality, and very few believed that the mental disorders are essentially untreatable.  Most participants believed that it is appropriate that the MHA enables compulsory re-feeding of patients with anorexia nervosa, and very few believed that the MHA should not be used to enforce admission to hospital for patients with anorexia nervosa. Very few participants also believed that the MHA should not be used when patients clearly believe that the advantages of anorexia nervosa outweigh the disadvantages, and less than half believed that the MHA should be used more frequently to protect the health and safety of patients with anorexia nervosa.  Bulimia nervosa was slightly more likely to be seen as culturally determined by women’s role in society, and anorexia nervosa was more likely to be seen as a neurophysiological disorder of unknown origin. Further, anorexia nervosa was slightly more likely to be perceived as abnormal behaviour in the context of a weak, manipulative or inadequate personality, and essentially untreatable. Differences between anorexia nervosa and bulimia nervosa were not examined with inferential statistics.  Non-consultants were significantly more likely to perceive anorexia nervosa as essential untreatable, compared to consultants. This was the only significant difference reported for grade.  Junior psychiatrists were significantly more likely to view bulimia nervosa as abnormal behaviour in the context of a weak, manipulative or inadequate personality, compared to psychiatrists of higher seniority. Also, senor psychiatrists were significantly more likely to believe that the MHA should not be used to enforce admission to hospital for patients with anorexia nervosa. No other significant differences were reported for seniority. |
| Jorm et al. (1999) | Clinical psychologists  Psychiatrists  GPs  General population  (Australia) | Cross-sectional survey  Vignettes were used | Between-groups ANOVA | Major depression (description)  Schizophrenia (description) | Long term outcomes following treatment compared to other people in the community  Negative  Be violent  Drink too much  Take illegal drugs  Have poor friendships  Positive  Understand other’s feelings  Have a good marriage  Be a caring parent  Be a productive worker  Be creative or artistic  Profession  Age (levels were not specified)  Sex  Payment method  Private practice  Salaried  Mixed  Frequency of contact with the mental disorders described (it was not clear if this was professional or personal contact) | For major depression, psychologists believed that negative outcomes were less likely, and positive outcomes were either more likely or slightly more likely. GPs and psychiatrists believed that some negative outcomes were more likely and slightly more likely, and some were less likely and slightly less likely. GPs only believed that one positive outcome was more likely, and one positive outcome was just as likely. Psychiatrists believed some positive outcomes were more likely and slightly more likely, and some positive outcomes were less likely and slightly less likely. For schizophrenia there was more consistency across the items and professions. All of the relevant participants believed negative outcomes were either more likely or slightly more likely. Positive outcomes were believed to be less likely by the relevant participants. The only two exceptions to this were GPs and psychologists believed that being creative or artistic was slightly more likely.  For the relevant participants, schizophrenia was stigmatised more than major depression for all items. The only exception to this was GPs and psychologists believed that a person with schizophrenia was more likely than a person with major depression to be creative or artistic. Differences between the mental disorders were not examined with inferential statistics.  Across the different items and disorders, each profession expressed more stigmatisation than the other professions at least once, and less stigmatisation at least once. However, psychologists often expressed less stigmatisation than the other two professions. Differences between the professions were either not examined with inferential statistics separately to the general population, or were not clearly examined with inferential statistics.  For GPs, age was not found to have a significant impact on negative outcomes for schizophrenia. Younger psychiatrists believed more that negative outcomes were more likely for both disorders, and there were no age differences for psychologists. It was not clear if these differences were statistically significant or not. Nothing else was reported for the impact of age on stigmatisation.  Female GPs believed more that negative outcomes were more likely for schizophrenia, and there were no sex differences for psychologists. Whether these results were examined with inferential statistics was not clear, and nothing else was reported for the impact of sex on stigmatisation.  Salaried psychiatrists believed more that negative outcomes were more likely for depression, compared to private practice psychiatrists, and psychologists in mixed practice believed more that positive outcomes were more likely for depression compared to salaried psychologists. Whether these differences were statistically significant was not reported, and nothing else was reported for the impact of payment method on stigmatisation.  Frequency of contact with the mental disorders described was not associated with stigmatisation for any profession or mental disorder. Whether this was explored with inferential statistics was not clear.  Other relevant findings were excluded from this table as they were not reported for mental health professionals separately. |
