# Supplementary material for: The stigmatization of mental illness by mental health professionals: Scoping review and bibliometric analysis
Source: PLoS One. 2023 Jan 20;18(1):e0280739. doi: 10.1371/journal.pone.0280739 (PMC9858369; doi:10.1371/journal.pone.0280739)
Supplement: S7 Appendix — (DOCX) [file pone.0280739.s007.docx]

| **Authors (year)** | **Populations**  **(countries)** | **Research methods** | **Analytical approaches** | **Disorders** | **Variables and measures** | **Findings** |
| --- | --- | --- | --- | --- | --- | --- |
| Kahle & White (1991) | Psychologists  Counsellors  (USA) | Cross-sectional survey | Chi-square test of independence | Alcoholism (label) | Causal attributions  It is somewhat difficult to feel truly empathic towards an alcoholic  I would feel uncomfortable being around alcoholics a lot  If over the course of  providing therapy for your client he/she continues to drink, after providing referral sources you would suspend/terminate the counselling relationship  Alcoholics can be cured  Profession | Most participants disagreed that alcoholics tend to be weak-willed individuals. However, only roughly half of the psychologists disagreed that alcoholism is a symptom of an underlying personality disorder, and only roughly half of the counsellors disagreed that an alcoholic is as blameless for his/her disease as a diabetic. In comparison, most of the counsellors disagreed that alcoholism is a symptom of an underlying personality disorder, and most of the psychologists disagreed that an alcoholic is as blameless for his/her disease as a diabetic.  Most participants disagreed that it is somewhat difficult to feel truly empathic towards an alcoholic. However, only roughly half disagreed that they would feel uncomfortable being around alcoholics a lot, and they would suspend/terminate the counselling relationship if over the course of  providing therapy the client continues to drink. Further, roughly half of the counsellors agreed that alcoholics can be cured, compared to most psychologists agreeing that alcoholics can be cured.  Profession was found to have a significant relationship with two causal attributions. Counsellors were more likely to agree that an alcoholic is as blameless for his/her disease as a diabetic, and psychologists were more likely to agree that alcoholism is a symptom of an underlying personality disorder.  Profession was found to have a significant relationship with the measure it is somewhat difficult to feel truly empathic towards an alcoholic and alcoholics can be cured. Psychologists were more likely to agree with both of these statements, compared to counsellors. Profession was not found to have a significant relationship with any other measure of stigmatisation. |
| Kahn (1976) | Psychiatric nurses  (USA) | Cross-sectional survey | - | Mental illness in general (label) | OMI scale  Authoritarianism  Benevolence  Mental hygiene ideology  Social restrictiveness  Interpersonal aetiology | Participants expressed more negative attitudes on the authoritarianism, benevolence, and social restrictiveness factors, and more positive attitudes on the mental hygiene ideology factor.  Participants expressed less agreement with interpersonal aetiology. |
| Kaitz et al. (2021) | Psychiatrists  Psychologists  Social workers  Unspecified nurses  Other unspecified providers within a Veteran’s Affairs healthcare system |  |  |  |  | Nothing more was reported for this study as findings were not reported for mental health professionals separately. |
| Kapungwe et al. (2011) | Clinical officers in psychiatry  Psychiatric nurses  General clinical officers  Primary care nurses  Environmental health technologists  (Zambia) | Cross-sectional survey | - | Mental illness in general (label) | Stereotypes  People with mental illness are dangerous  If people become mentally ill once, they easily become ill again  People with mental illness have unpredictable behaviour  Level of comfort when working with mental illness  Restrictiveness  Violent mental patients should be handcuffed  Detention in a solitary place should be considered for people with mental illness  Sedation of mental patients would guarantee safety for other people in all cases  Profession  Level of psychiatric nurse training  Registered  Enrolled | Most general clinical officers did not agree with the three stereotypes (neutral responses were available).  For the clinical officers in psychiatry and general clinical officers, most of the participants were uncomfortable working with mental illness, and clinical officers in psychiatry were more likely to be uncomfortable. For the psychiatric nurses, most enrolled nurses felt uncomfortable working with mental illness, whereas all of the registered nurses were comfortable. These differences between the professions were not examined with inferential statistics.  Clinical officers in psychiatry were more likely to be restrictive overall compared to the other mental health professionals. This was not assessed with inferential statistics.  Other relevant findings were excluded from this table as they were not reported for mental health professionals separately. |
| Kassam et al. (2012) | Social workers  Psychiatric nurses  Counsellors  Psychologists  Occupational therapists  Recreational therapists  Unspecified nurses  Unspecified physicians  Pharmacists  Social work students  Psychiatric nursing students  Psychology graduate students  Occupational therapy students  Nursing students  Medical students  Other unspecified health professionals |  |  |  |  | Nothing more was reported for this study as findings were not reported for mental health professionals separately. |
| Kerr et al. (1995) | Psychiatrists  GPs  (Wales) | Cross-sectional survey | Independent samples t-test | Depression (label) | DAQ  Treatment attitude  Most depressive disorders seen in general practice improve without medication  An underlying biochemical abnormality is at the basis of severe cases of depression  Becoming depressed is a way that people with poor stamina deal with life difficulties  Psychotherapy tends to be unsuccessful with depressed patients  Antidepressants usually produce a satisfactory result in the treatment of depressed patients in general practice  If psychotherapy were freely available, it would be more beneficial than antidepressants for most depressed patients  Professional ease  I feel comfortable in dealing with depressed patients’ needs  Working with depressed patients is heavy going  It is rewarding to spend time looking after depressed patients  Psychotherapy for depressed patients should be left to a specialist  Depression malleability  Depression reflects a characteristic response in patients that is not amenable to change  Becoming depressed is a natural part of being old  Depressed patients are more likely to have experienced deprivation in early life than other people  If depressed patients need antidepressants, they are better off with a psychiatrist than a GP  Depression identification  The majority of depression seen in general practice originates from patients’ recent misfortunes  It is difficult to differentiate whether patients are presenting with unhappiness or with clinical depressive disorder that needs treatment  There is little to be offered to those depressed patients who do not respond to what GPs do  The practice nurse could be a useful person to support depressed patients  During the past five years, I have seen an increase in the number of patients presenting with depressive symptoms  It is possible to distinguish two main groups of depression: one psychological in origin and the other caused by biochemical mechanisms  Profession | Participants agreed more that an underlying biochemical abnormality is at the basis of severe cases of depression, and disagreed more that becoming depressed is a way that people with poor stamina deal with life difficulties. Also, participants disagreed more with the depression malleability factor. The remaining DAQ items were either not summarised separately or were not included in this table due to being irrelevant to stigmatisation.  Compared to GPs, psychiatrists agreed significantly more with the item an underlying biochemical abnormality is at the basis of severe cases of depression, and disagreed significantly more with the item becoming depressed is a way that people with poor stamina deal with life difficulties. No significant difference was found between GPs and psychiatrists for the depression malleability factor. However, GPs agreed significantly more than psychiatrists that depression reflects a characteristic response in patients that is not amenable to change, and becoming depressed is a natural part of being old. Professions were not compared for the remaining relevant DAQ items. |
| Keuroghlian et al. (2016) | Counsellors  Social workers  Psychiatrists  Psychologists  Primary care physicians  Unspecified nurses  Internists  Physician assistants  Psychiatry residents |  |  |  |  | Nothing more was reported for this study as findings were not reported for mental health professionals separately. |
| Kingdon et al. (2004) | Psychiatrists  Trainee psychiatrists  (UK) | Cross-sectional survey | - | Mental illness in general (label)  Schizophrenia (label) | Causal attributions  A woman (or man) would be foolish to marry someone who has suffered from mental illness  Most women formerly in a mental hospital can be trusted as babysitters  Perceived unpredictability  Perceived difficulty to talk to  People with schizophrenia feel the same way we all do at times  Grade  Consultant  Other grades | Roughly half of the consultant psychiatrists believed schizophrenia is caused by biological factors, and a balance of biological and social factors. In comparison, most psychiatrists from other grades believed that schizophrenia is caused by biological factors, and under half believed schizophrenia is caused by a balance of biological and social factors.  Most of the psychiatrists did not agree that a woman (or  man) would be foolish to marry someone who has suffered from mental illness. Consultant psychiatrists agreed with this more than psychiatrists from other grades (neutral responses were available).  Most consultant psychiatrists believed that most women formerly in a mental hospital can be trusted as babysitters, and roughly half of the psychiatrists from other grades agreed with this statement (neutral responses were available).  Under half of the psychiatrists perceived schizophrenia as unpredictable, difficult to talk to, and feeling the way we do at times. There was no difference between the grades for perceived unpredictability, and consultant psychiatrists were more likely to agree with the other two items compared the psychiatrists from other grades.  Differences between the grades were not examined with inferential statistics.  Other relevant findings were excluded from this table as they were not reported for psychiatrists separately. |
| Kirkby & James (1979) | GPs  Psychiatrists  Other unspecified non-psychiatric medical practitioners  Other unspecified medical practitioners |  |  |  |  | Nothing more was reported for this study as findings were not reported for mental health professionals separately. |
| Kloss & Lisman (2003) | Counsellors  Unspecified nurses working in mental health or alcohol rehabilitation facilities  Other unspecified mental health clinicians  (USA) | Cross-sectional survey  Vignettes were used | Correlation analysis  Independent samples t-test  Between-groups ANOVA | Alcoholism (description and label)  Schizophrenia (description and label)  Comorbid alcoholism and schizophrenia (description and label) | Perceived blame  The target is responsible for his problems  The target could have avoided the problems that he has  The target could have controlled the cause of his problems  Perceived control  The target is personally responsible for creating a solution  The target can overcome his problems by himself  The target can control the solution to his problems  Endorsement of the alcoholism disease model (items were not specified)  Agency type  Mental health  Alcohol rehabilitation  Whether the participants are recovering alcoholics  Whether the participant is a certified addiction counsellor  Whether participants refer patients to Alcoholics Anonymous | Overall perceived blame and control was low. Participants also endorsed the alcoholism disease model to a moderate to high degree.  Endorsement of the alcoholism disease model was not found to be significantly correlated with overall perceived blame.  Participants from the mental health facility blamed comorbid alcoholism and schizophrenia significantly more than participants from the alcohol rehabilitation facility. The impact of agency type on perceived blame was not examined for the other mental disorders.  Agency type was not found to have a significant impact on perceived control.  Recovering alcoholics attributed significantly less blame to alcoholism compared to participants not recovering from alcoholism. This was not clearly examined for the other mental disorders, and this variable was not found to have a significant impact on endorsement of the alcoholism disease model.  Certified addiction counsellors attributed significantly less blame to alcoholism compared to the other participants. This was not clearly examined for the other mental disorders, and this variable was not found to have a significant impact on endorsement of the alcoholism disease model.  Participants that refer patients to Alcoholics Anonymous attributed significantly less blame to alcoholism compared participants that do not make this referral. This was not clearly examined for the other mental disorders, and this variable was not found to have a significant impact on endorsement of the alcoholism disease model. |
| Knaak et al. (2015) | Social workers  Counsellors  Occupational therapists  Psychologists  Psychiatrists  Directors  Managers  Unspecified nurses  Other unspecified health professionals  Unspecified students |  |  |  |  | Nothing more was reported for this study as findings were not reported for mental health professionals separately. |
| Kopera et al. (2015) | Psychiatrists  Psychotherapists  (Poland) | Cross-sectional survey  GNAT | Correlation analysis | Mental illness in general (label)  Schizophrenia, depressive, and neurotic (labels; only these examples were given for the GNAT) | Emotions  Positive  Compassion  Interest  Sadness  Acceptance  Negative  Anger  Dislike  Anxiety  Aversion  Distrust  Indifference  OMI scale  Authoritarianism  Benevolence  Mental hygiene ideology  Social restrictiveness  Interpersonal aetiology  Implicit attitudes (only the following example was provided)  General attitudes  Age | Participants reported more overall positive emotions and less overall negative emotions towards mental illness.  Participants expressed low authoritarianism and low social restrictiveness towards mental illness. Scores for the other OMI factors were not reported by the authors.  Participants expressed negative implicit attitudes towards mental illness (exemplified by at least the terms schizophrenia, depressive, and neurotic).  Age was not found to be significantly correlated with any of the stigmatisation measures overall. |
| Koutrelakos et al. (1978) | Psychologists  Social workers  Business administration officers |  |  |  |  | Nothing more was reported for this study as findings were not reported for mental health professionals separately. |
| Krawitz (2004) | Psychologists  Social workers  Occupational therapists  Unspecified nurses from mental health and substance abuse services  Unspecified doctors from mental health and substance abuse services  (Australia) | Longitudinal survey  An intervention was used | Repeated-measures ANOVA  One analysis was not made clear | BPD (label) | Willingness to work with BPD  Optimism in working with BPD  Age  Profession  Work setting (levels were not made clear) | Before an educational workshop on BPD, participants were more willing to work with BPD and more optimistic in working with BPD.  Postintervention participants were significantly more willing and optimistic. Six months after the intervention participants were less willing and optimistic than at postintervention, but were still significantly more willing and optimistic compared to before the intervention. The differences between postintervention and six months after the intervention were not found to be statistically significant.  Age, profession, and work setting were not found to be related to differences across the time points. Inferential statistics were not reported for this. |
| Kua et al. (2000) | GPs  Psychiatrists  Other unspecified doctors  (Singapore) | Experiment (GPs were randomly assigned to vignettes)  Cross-sectional survey (Psychiatrists were assigned to all the vignettes)  Vignettes were used | - | Major depression (description)  Schizophrenia (description)  Mania (description) | Long term outcomes compared to other people in the community  Negative  Be violent  Drink too much  Take illegal drugs  Have poor friendships  Positive  Understand other’s feelings  Have a good marriage  Be a caring parent  Be a productive worker  Be creative or artistic  Prognosis with and without professional help  Profession | For depression, mental health professionals rated all the negative outcomes as less likely and all the positive outcomes as more likely. The pattern of responses for mania and schizophrenia were not as straight forward. For mania, all negative outcomes were rated as less likely, and some of the positive outcomes were rated as more likely. However, GPs rated three of the positive outcomes as roughly as likely, and psychiatrists rated one of the positive outcomes as less likely and one as roughly as likely. For schizophrenia, GPs rated all of the negative outcomes as less likely, and the psychiatrists rated three of the negative outcomes as less likely, and one as more likely. Also, psychiatrists rated all positive outcomes as less likely, and GPs rated one of the positive outcomes as more likely, and one as less likely. For the remaining positive outcomes, GPs rated them as roughly as likely.  For all of the mental disorders, most mental health professionals gave poor prognoses without professional help. In contrast, most mental health professionals gave good prognoses with professional help (don’t know was an available option). This difference between receiving professional help and not receiving professional help was not examined with inferential statistics.  For GPs, there was no consistent difference between the mental disorders across the negative outcomes. For positive outcomes, GPs mostly stigmatised schizophrenia the most, followed by mania, followed by depression. The only exception to this was for one of the positive outcomes, mania was stigmatised the most, followed by schizophrenia, followed by depression. For psychiatrists, schizophrenia was stigmatised more than mania and depression for one negative outcome. In this case mania and depression were stigmatised to the same degree. For all remaining items (including prognosis items), mental health professionals stigmatised more or were more likely to stigmatise schizophrenia, followed by mania, followed by depression. Differences between the mental disorders were not examined with inferential statistics.  Across the mental disorders, for some items GPs expressed more or were more likely to express stigmatisation, whereas for other items, psychiatrists expressed more or were more likely to express stigmatisation. Differences between GPs and psychiatrists were not examined with inferential statistics separately to the other unspecified doctors. |
| Kukulu & Ergun (2007) | Unspecified nurses working in psychiatric wards  (Turkey) | Cross-sectional survey | - | Schizophrenia (label) | Causal attributions  Social distance  People diagnosed with schizophrenia are aggressive  People diagnosed with schizophrenia are not able to make correct decisions about their own lives  People with schizophrenia never completely recover  Schizophrenia is a treatable illness | Most participants agreed that schizophrenia is an illness, and disagreed that schizophrenia is a state and contagious. Further, most participants agreed that schizophrenia is an illness present from birth, and roughly half agreed that schizophrenia is caused by social problems. However, roughly half of the participants also agreed that schizophrenia is a state of emotional weakness (I don’t know was an available option).  Overall, either roughly half or most of the participants expressed social distance towards schizophrenia. The only exception to this was most participants disagreed that people diagnosed with schizophrenia should not be allowed to  move freely within society (I don’t know was an available option).  Most participants agreed that people diagnosed with schizophrenia are aggressive and are not able to make correct decisions about their own lives (I don’t know was an available option).  Most participants agreed that people with schizophrenia never completely recover, but most participants also agreed that schizophrenia is a treatable illness (I don’t know was an available option). |
| Kusalaruk et al. (2015) | Psychiatrists  (Thailand) | Cross-sectional survey | Chi-square test of independence  Fisher's exact test | OCD (label)  Other unspecified mental disorders (label) | Positive feelings  Admiration  Pity  Understanding  Empathy  Negative feelings  Tiredness  Annoyance  Perceived difficulty  People with OCD are not compliant to treatment  I don’t want to treat people with OCD compared to other mental disorders  People with OCD require more time compared to other mental disorders  People with OCD require more patience compared to other mental disorders  People with OCD talk to much compared to other mental disorders  People with OCD are more difficult to build a therapeutic relationship with compared to other mental disorders  Years of professional experience  1-5  6-10  11-20  >20  Workplace  Mental hospital  Medical university hospital  General/provincial hospital  Private hospital/clinic/others  Estimated number of outpatients treated over a three hour period  1-10  11-20  21-30  >30  Estimated number of OCD patients treated  1-10  11-20  21-30  >30  Estimated time spent on visits with OCD patients  1-15 minutes  15-30 minutes  30-45 minutes  >45 minutes  Estimated time spent in follow-up sessions with OCD patients  1-15 minutes  15-30 minutes  30-45 minutes  >45 minutes  Experience and proficiency with exposure and response prevention therapy  None  Known but never practice  Practice but not proficient  Proficient  Overall confidence in treating OCD  Confidence in treating OCD with behaviour therapy  Confidence in treating OCD with medication  Preferred treatment mode for OCD  Drugs only  Drugs and behaviour therapy  Drugs and other psychotherapy  Drugs, behaviour therapy, and other psychotherapy  Sex  Age  <35  35-45  >45 | Most participants expressed positive attitudes towards OCD across the measures and items. The only exceptions to this were most participants disagreed with admiring people with OCD, most participants agreed that people with OCD are difficult, and roughly half of the participants agreed that people with OCD exhibit poor compliance with behaviour therapy.  Years of professional experience was significantly associated with annoyance and the perception that people with OCD have a poor compliance with medication. Participants with six to ten years of experience reported these responses the most often. Nothing else was reported for the relationship between years of professional experience and stigmatisation.  Workplace was significantly associated with annoyance. Participants in the general/provincial hospitals felt this the most often, followed by  participants in the mental hospitals, followed by participants in the medical university hospitals. Nothing else was reported for the relationship between workplace and stigmatisation.  Estimated number of outpatients treated over a three hour period was significantly associated with admiration. Participants with less than ten patients were most likely to report admiration. Estimated number of outpatients treated over a three hour period was also significantly associated with the perception that people with OCD have a poor compliance with behaviour therapy, and people with OCD require more time compared to other mental disorders. Participants with more than 30 patients were the most likely to agree with these responses. Nothing else was reported for the relationship between estimated number of outpatients treated over a three hour period and stigmatisation.  Estimated number of OCD patients treated was significantly associated with the perception that people with OCD have a poor compliance with behaviour therapy. Participants that had treated 11 to 20 patients were the least likely to agree with this perception. Nothing else was reported for the relationship between estimated number of OCD patients treated and stigmatisation.  Estimated time spent on visits with OCD patients was significantly associated with pity. Participants that spent less than 15 minutes with OCD patients were the least likely to feel pity, and participants that spent more than 45 minutes with OCD patients were the most likely to feel pity. Estimated time spent on visits with OCD patients was also significantly associated with the perception that people with OCD have a poor compliance with behaviour therapy. All the participants that spent less than 15 minutes with OCD patients agreed with this perception, and roughly a third of the participants that spent more than 45 minutes with OCD patients agreed with this perception. Nothing else was reported for the relationship between estimated time spent on visits with OCD patients and stigmatisation.  Estimated time spent in follow-up sessions with OCD patients was significantly associated with the view that people with OCD are more difficult to build a therapeutic relationship with compared to other mental disorders. Participants that spent more than 30 minutes in follow-up sessions with OCD patients were the most likely to agree with this view. Nothing else was reported for the relationship between estimated time spent in follow-up sessions with OCD patients and stigmatisation.  Experience and proficiency with exposure and response prevention therapy was significantly associated with admiration and pity. Most proficient participants felt admiration towards people with OCD, and all proficient participants felt pity towards people with OCD. In comparison, none of the participants with no experience felt admiration, and half of these participants felt pity. Experience and proficiency with exposure and response prevention therapy was significantly associated with tiredness. A small proportion of proficient participants felt tired, and most of the participants without experience felt tired. Experience and proficiency with exposure and response prevention therapy was significantly associated with the perception that people with OCD have a poor compliance with medication. Proficient participants were most likely to agree with this perception, and none of the participants without experience agreed with this perception. Experience and proficiency with exposure and response prevention therapy was also significantly associated with the view that people with OCD require more time than other mental disorders. Participants either without any experience or without practice experience were the most likely to agree with this, whereas none of the proficient participants agreed with this view. Nothing else was reported for the relationship between experience and proficiency with exposure and response prevention therapy and stigmatisation.  Overall confidence in treating OCD was significantly associated with tiredness and annoyance. Confident participants were less likely to feel tired and annoyed compared to participants that lacked confidence. Overall confidence in treating OCD was significantly associated perceived difficulty. Most confident participants agreed with this perception, however all of the participants that lacked confidence agreed with this perception. Overall confidence in treating OCD was significantly associated with not wanting to treat people with OCD compared to other mental disorders. Confident participants were less likely to agree with this compared to participants without confidence. Overall confidence in treating OCD was also significantly associated with the view that people with OCD are more difficult to build a therapeutic relationship with compared to other mental disorders. Again, confident participants were less likely to endorse this view compared to participants without confidence. Nothing else was reported for the relationship between overall confidence in treating OCD and stigmatisation.  Confidence in treating OCD with behavioural therapy was significantly associated with the view that people with OCD exhibit poor compliance with other psychotherapy, and people with OCD require more patience than other mental disorders. These variables were reportedly related to confidence in treating OCD with behaviour therapy in a similar way to how variables were related to overall confidence in treating OCD. Nothing else was reported for the relationship between confidence in treating OCD with behaviour therapy and stigmatisation.  Confidence in treating OCD with medication was significantly associated with pity and tiredness. Nothing else was reported for the relationship between confidence in treating OCD with medication and stigmatisation.  Preferred treatment mode for OCD, sex, and age were not found to be significantly associated with stigmatisation. |
| Kuyken et al. (1992) | Clinical psychologists  (England) | Semi-structured interviews | - | Depression (label) | Causal attributions | Participants attributed depression to a range of causes with varying proportions. Life-events or traumatic experiences were among the most likely causes, and loneliness or poor social support were among the least likely causes.  The following aetiological approaches are listed from the most explanatory power to the least, according to the participants.  Diathesis-stress approach  Cognitive approach  Psychodynamic approach  Biological approach  Differences between aetiological approaches were not assessed with inferential statistics. |
| Lam et al. (2013) | Family physicians  (China) | Cross-sectional survey with open-ended questions and experiment  Vignettes were used | Thematic analysis  Multiple ordinal logistic regression analysis | Mental illness in general (label)  Depression (description)  Schizophrenia (description)  Other unspecified patients (label) | Perceived difficulty  Perceived helplessness/prognosis  Sympathy  People with mental illness are unfortunate  Patience  People with mental illness are vulnerable  Perceived unpredictability  I would like to have the target on my practice list for their mental issues  I would not like to have the target on my practice list for their physical issues  I will spend more time on the target than other patients  The target arouses sympathy  The target is likely to annoy me  I am worried that I am unable to predict how the target would behave or respond  I am afraid to ask the target personal questions  I will avoid confronting the target directly  I will have greater distance with the target than other patients  I expect the target to be more demanding than other patients  I doubt the ability of the target to be involved in treatment decisions  I believe the target will comply with treatment and advice  The target is likely to have improvement if treated  Years of professional experience  Sex  Setting  Hospital  Community  Sector  Public  Private  Practice  Solo  Group  Have a relative/friend with a mental illness | Participants described mental illness via a number of adjectives with varying proportions. Challenging, time consuming, demanding, difficult, and helpless were among the most likely adjectives. Sympathy, curable, unfortunate, patience, troublesome, bizarre, vulnerable, and unpredictable were among the least likely adjectives. Also, chronic was stated slightly less frequently than curable.  For some of the target specific measures, most participants expressed positive attitudes towards the mental disorders, and for a few other measures most participants expressed negative attitudes. Also, for some measures, either roughly half, less than half, or just under half of the participants expressed positive attitudes (neutral responses were available).  For eight of the target specific measures schizophrenia was significantly more likely to be stigmatised than depression. Depression was significantly more likely to be stigmatised than schizophrenia for only one of the measures. Mental disorder was not found to be a significant predictor of the remaining measures.  For two of the target specific measures, participants with more professional experience were significantly less likely to stigmatise the mental disorders compared to participants with less professional experience. For one measure participants with more professional experience were significantly more likely to stigmatise the mental disorders. Years of professional experience was not found to be a significant predictor of the remaining measures.  For two of the target specific measures, females were significantly more likely to stigmatise the mental disorders compared to males. Sex was not found to be a significant predictor of the remaining measures.  For three of the target specific measures, participants from a community setting were significantly more likely to stigmatise the mental disorders compared to participants from a hospital setting. Setting was not found to be a significant predictor of the remaining measures.  For two of the target specific measures, participants from the private sector were significantly more likely to stigmatise the mental disorders compared to participants from the public sector. Sector was not found to be a significant predictor of the remaining measures.  For one of the target specific measure, participants that practice in a group format were significantly less likely to stigmatise the mental disorders compared to participants that practice in a solo format. Practice was not found to be a significant predictor of the remaining measures.  For four of the target specific measures, participants without a relative/friend with a mental illness were significantly more likely to stigmatise the mental disorders, compared to participants with a relative/friend that has a mental illness. For one measure, participants with a relative/friend that has a mental illness were significantly more likely to stigmatise mental illness. Having a relative/friend with a mental illness was not found to be a significant predictor of the remaining measures. |
| Lammie et al. (2010) | Unspecified nurses from forensic mental health settings  Nursing assistants |  |  |  |  | Nothing more was reported for this study as findings were not reported for nurses separately. |
| Lampe et al. (2013) | GPs  Psychiatrists  (Australia) | Cross-sectional survey | Independent samples t-test  Correlation analysis | Depression (label) | DAQ (only factors relevant to stigmatisation were included in this table)  Treatment attitudes  Inevitable course of depression/pessimism about depression  Profession  NEO-FFI  Neuroticism  Extraversion  Openness  Agreeableness  Conscientiousness | Participants agreed slightly more with treatment attitudes, suggesting more agreement with attributing depression to poor stamina/a biochemical abnormality. However, GPs disagreed more with inevitable course of depression/pessimism about depression, and psychiatrists responded roughly neutrally to this factor.  Profession was not found to have a significant impact on treatment attitudes. Psychiatrists agreed significantly more with inevitable course of depression/pessimism about depression compared to GPs.  For GPs, extraversion was significantly negatively correlated with both DAQ factors, and neuroticism was significantly positively correlated with inevitable course of depression/pessimism about depression. No other significant correlations were found between the NEO-FFI and the DAQ for GPs. For psychiatrists, no significant correlations were found between the NEO-FFI and the DAQ. |
| Lauber et al. (2004) | Psychiatrists  (Switzerland) | Structured telephone interviews  A vignette was used | - | Mental illness in general (label)  Schizophrenia (description and label) | Social distance  Mental health facilities should be kept out of residential neighbourhoods  Local residents have good reason to resist the location of mental health services in their neighbourhood  Local residents should accept the location of mental health facilities in their neighbourhood to serve the needs of the local community  Locating mental health facilities in a residential area downgrades the neighbourhood  Locating mental health services in a residential neighbourhood does endanger local residents  It is frightening to think of people with mental problems living in residential neighbourhoods | Participants expressed more social distance towards schizophrenia for some items, and less social distance for other items. Participants expressed social distance on most of the items.  Overall, participants expressed a lack of stigmatisation for the remaining measures. |
| Lauber et al. (2006) | Psychologists  Psychiatrists  Social workers  Unspecified nurses from psychiatric wards  Physiotherapists  Vocational workers |  |  |  |  | Nothing more was reported for this study as findings were not reported for mental health professionals separately. |
| Lawrie et al. (1998) | GPs  (Scotland) | Experiment  Vignettes were used | Mann-Whitney *U*-test  Correlation analysis | Schizophrenia (description and label)  Depression (description and label)  Unspecified patients (label) | You would be happy to have the target on your list  The target is likely to take up a lot of time  The target is more likely to be violent than most patients  The target is unlikely to comply with advice or treatment given  You would be concerned about the welfare of the target’s child  The target is likely to drink to excess  The target is likely to take illegal drugs  The target arouses your sympathy  Age | For most of the measures, participants expressed more positive attitudes to both mental disorders. The only two exceptions to this were participants responded neutrally to the measure you would be concerned about the welfare of the target’s child, and agreed more with the measure this person is likely to take up a lot of time.  For most of the measures there was either no difference between schizophrenia and depression, or differences between schizophrenia and depression were not found to be statistically significant. The only significant difference between schizophrenia and depression was participants were significantly less happy to have a patient with schizophrenia on their list.  Age was not found to be significantly correlated with any of the measures. |
| Lawrie et al. (1996) | GPs  (Scotland) | Cross-sectional survey  A vignette was used | - | Schizophrenia (description and label)  Unspecified patients (label) | You would be happy to have the target on your list  The target is likely to take up a lot of time  The target is more likely to be violent than most patients  The target is unlikely to comply with advice or the treatment given  The target is likely to drink to excess  The target is likely to take illegal drugs  The target arouses your sympathy  You would be concerned why the target is wanting to join your practice | Overall, participants expressed either neutral or more positive responses. The only exception to this was participants agreed more that the target is likely to take up a lot of time. |
| Lebowitz & Ahn (2014) | Psychiatrists  Psychologists  Social workers  Counsellors  Other unspecified mental health professionals  (USA) | Experiment  Vignettes were used | Correlation analysis  Mixed factorial ANOVA  Paired samples t-test | Schizophrenia (description)  Social phobia (description)  Depression (description)  OCD (description) | Empathy  Sympathetic  Soft-hearted  Warm  Compassionate  Tender  Moved  Personal distress  Alarmed  Troubled  Distressed  Upset  Disturbed  Worried  Prognosis without treatment  Explanations provided for the mental disorders  Biological/both explanations but the biological explanation is prominent  Psychosocial/both explanations but the psychosocial explanation is prominent  Profession  Medical doctors  Non-medical doctors (e.g., counsellors) | Across the different conditions and professions, participants expressed more empathy at times and less empathy at times. Mostly, participants either expressed slightly less empathy or slightly more empathy towards the mental disorders. Participants expressed less personal distress towards the mental disorders. For all mental disorders, participants believed that the likelihood of improvement without treatment was less than 50%. Also, participants believed that the mental disorders would persist for somewhere between one month to five years. None of the participants believed that the mental disorders were indefinite.  Across the different conditions, professions, and measures of stigmatisation, participants expressed positive and negative attitudes towards the mental disorders to varying degrees. However, no consistent mental disorder differences emerged, and every mental disorder was stigmatised more than every other mental disorder at least once. Differences between the mental disorders were not examined with inferential statistics.  The correlation between overall empathy and overall personal distress was investigated in the first and second studies. Across the different conditions there was a positive correlation between these two variables. However, in most cases the correlations were not found to be statistically significant.  Biological/prominent biological explanations elicited significantly less overall empathy than psychosocial/prominent psychosocial explanations for all mental disorders. Explanation was not found to have a significant impact on overall personal distress. The only exception to this was biological explanations elicited significantly more overall personal distress for schizophrenia. For the first and second studies, biological explanations elicited significantly worse prognoses for one of the prognosis items, compared to psychosocial explanations (for all of the mental disorders). However, explanation was not found to have a significant impact on the other prognosis item for the first and second studies (for all of the mental disorders), and explanation was not found to have a significant impact on prognosis for the third study (this study was with social phobia and depression only).  Medical doctors expressed significantly less empathy and significantly more personal distress than non-medical doctors. However, it was not reported whether the effect of profession on personal distress was significant for the second study. In this study, medical doctors expressed more personal distress than non-medical doctors in all cases but one. Non-medical doctors expressed slightly more personal distress towards depression in the biological condition compared to medical doctors.  For the first and second studies, profession was not found to moderate the relationship between explanation and overall empathy. Also, for the first study profession was not found to moderate the relationship between explanation and overall personal distress. Whether this was also the case for the second study was not reported. However, in the third study, prominent biological explanations elicited significantly less overall empathy for non-medical doctors, and explanation was not found to have a significant impact on overall empathy for medical doctors. Similarly, in the second study biological explanations elicited a significantly worse prognosis for one item among non-medical doctors, and explanation was not found to have a significant impact on this prognosis item for medical doctors. Although profession was found to moderate the relationship between explanation and one of the prognosis items in the third study, the nature of this moderation was not reported. Whether this moderation effect emerged for the first study in general, or the other prognosis items in the second and third studies was not reported. |
| Lebowitz & Ahn (2016) | Psychiatrists  Psychologists  Social workers  Other unspecified mental health professionals  (USA) | Experiment  Vignettes were used  An intervention was used | Paired samples t-test  Mixed ANOVA | Depression (description) | Empathy  Sympathetic  Soft-hearted  Warm  Compassionate  Tender  Moved  Personal distress  Alarmed  Troubled  Distressed  Upset  Disturbed  Worried  Social distance  Profession | The intervention included a personification component and an agency reorientation component. The former component involved providing the participants with a photo purportedly depicting the targets and non-diagnostic personal information about the target. The latter component involved providing participants with the information that the target was currently in the process of making a decision about which clinician they should see. The control group was provided with an apparently real fMRI image of the targets brain.  Participants expressed slightly more overall empathy, less overall personal distress, and more social distance.  Intervention was not found to have a significant impact on overall empathy and overall personal distress. Participants in the intervention group expressed significantly less social distance than participants in the control group. Although the intervention was not found to have an impact on overall empathy, overall empathy was significantly negatively correlated with social distance for participants in the intervention group.  Profession was not found to moderate the effect of intervention on overall empathy, overall personal distress, and social distance. |
| Lester et al. (2005) | GPs  Practice nurses  (England) | Focus groups | Discourse analysis | Mental illness in general (label)  Psychosis (label)  Recurrent depression (label) | Perceived difficulty to communicate with  People with mental illness regularly do not attend appointments and present when the GP is not available  Appointment non-attendance by people with mental illness reflects irrational behaviour and a chaotic lifestyle  Prognosis | GPs suggested that it can be difficult to communicate effectively with people with mental illness.  One GP perceived people with mental illness as regularly not attending appointments and presenting when the GP is not available. Appointment non-attendance by patients with mental illness was described by most GPs as reflecting irrational behaviour and a chaotic lifestyle.  Most if not all GPs saw psychosis and recurrent depression as chronic lifelong conditions.  Other relevant findings were excluded from this table as they were not reported for GPs separately. |
| Levitt et al. (1963) | Unspecified nurses from a drug addiction facility  A social worker  A vocational rehabilitation counsellor  Aides  A purchasing agent  A barber  Manual arts therapists  A cook  A security aide  Financial management employees  Clerical workers |  |  |  |  | Nothing more was reported for this study as findings were not reported for mental health professionals separately. |
| Lewis & Appleby (1988) | Psychiatrists  (England, Wales, Scotland) | Experiment  Vignettes were used | Factorial ANOVA  Correlation analysis | Personality disorder (description and label)  Depression (description and label)  Anxiety state (label)  Adjustment reaction (label)  Neurasthenia (label)  Drug dependence (label) | The target is manipulative  If the target had an overdose it would be to seek attention  Would not like to have the target in one’s clinic  The target poses difficult management problems  The target is unlikely to complete treatment  The target is unlikely to comply with advice/treatment  The target is likely to become dependent on me  Prognosis  Annoyance  Sympathy  Diagnosis  Years of professional experience  The target is not mentally ill  The cause of the target’s debt is under their control  Suicidal urges are under the target’s control  The target’s case does not merit National Health Service time  The target should be discharged from out-patient follow up | For depression, participants expressed more positive attitudes for most of the stigmatisation measures. The only exception to this was participants agreed more that the target with depression is likely to become dependent on me. For personality disorder, participants expressed positive, negative, and roughly neutral responses across the measures of stigmatisation. Scores for each measure were not reported for the remaining mental disorders.  Personality disorder elicited significantly more stigmatisation than depression for most of the measures. However, personality disorder was not found to be significantly different to depression for the measure the target is likely to become dependent on me.  Diagnosis had a significant impact on some of the measures of stigmatisation, but was not found to have a significant impact on other measures of stigmatisation. Although, differences between the diagnoses were not specified or examined with multiple comparisons.  There was a significant vignette (personality disorder or depression) by diagnosis interaction effect for the target is manipulative. All that was noted was participants that diagnosed the target in the personality disorder vignette with depression endorsed this stereotype more than the participants who received the depression vignette. It was also noted that for the most part no other interaction between vignette and diagnosis were found for the measures of stigmatisation. It was concluded by the authors that personality disorder was stigmatised more than depression independent of participant diagnosis.  Believing that if the target had an overdose it would be to seek attention was significantly positively correlated with believing that the target is manipulative, not expressing sympathy, and annoyance. Believing the target is manipulative was significantly positively correlated with not expressing sympathy, but was not found to be significantly correlated with annoyance. Not expressing sympathy was significantly positively correlated with annoyance.  Participants with more experience expressed more negative attitudes for several measures of stigmatisation. This was not assessed with inferential statistics, and only annoyance was provided as an example.  Believing that the target is not mentally ill, that the cause of the target’s debt and suicidal urges are under their control, and that the target’s case does not merit National Health Service time was significantly positively correlated with believing that if the target had an overdose it would be to seek attention, believing that the target is manipulative, not expressing sympathy, and annoyance.  Believing that the target should be discharged from out-patient follow up was significantly positively correlated with believing that if the target had an overdose it would be to seek attention, believing that the target is manipulative, and not expressing sympathy. This variable was not found to be significantly correlated with annoyance. |
| Linden & Kavanagh (2012) | Mental health nurses  (Ireland) | Cross-sectional survey | MANOVA | Mental illness in general (label) | CAMI questionnaire  Authoritarianism  Benevolence  Social restrictiveness  Community mental health ideology  Social distance  Work setting  Inpatient  Community  Years of professional experience  1-4 years  5-9 years  10-14 years  15-19 years  20-24 years  25-29 years  30-34 years  35-39 years | Participants displayed more positive attitudes on all measures.  Compared to participants from a community setting, participants from an inpatient setting expressed significantly more negative attitudes for social restrictiveness and social distance, and significantly less positive attitudes for community mental health ideology. Work setting was not found to have a significant impact on authoritarianism and benevolence.  Years of experience was found to have a significant impact on social distance. Participants with 10-14 years of experience expressed significantly more positive attitudes than participants with 5-9 years of experience. No other significant differences were found for social distance. Years of experience was not found to have a significant impact on any of the CAMI factors. |
| Loch et al. (2013) | Psychiatrists  (Brazil) | Structured interviews | Generalized linear model | Schizophrenia (label) | Positive stereotypes  Creative  Healthy  Self-controlled  Gifted  Reasonable  Negative stereotypes  Dangerous  Unpredictable  Stupid  Bedraggled  Abnormal  Unreliable  Weird  Social distance  Age  Personal experience with psychological treatment  Never sought help  Sought help, no medication  Sought help, medication | Participants attributed overall positive stereotypes to schizophrenia less and attributed overall negative stereotypes to schizophrenia more. However, participants also expressed little social distance towards schizophrenia.  Older age was a significant predictor of less overall negative stereotypes.  Having never sought help was a significant predictor of less overall negative stereotypes, compared to having sought help, medicated. Having sought help, no medication, was not found to be a significant predictor of overall negative stereotypes, compared to having sought help, medication. Having never sought help and having sought help, no medication, were not compared. |
| Loch et al. (2011) | Psychiatrists  (Brazil) | Structured interviews | Correlation analysis  Chi-square test of independence  Stepwise multiple logistic regression analysis | Schizophrenia (label) | Positive stereotypes (items were not specified)  Negative stereotypes (items were not specified)  Social distance  Perceived negative attitudes in the general population (items were not specified)  Acceptance of psychotropic medication side-effects (items were not specified)  Attitude towards psychotropic medication (items were not specified)  Age  <30  31-40  41-50  >50  Sex  Working in a university hospital  Having children  Level of training  No doctoral degree  Doctoral/post-doctoral degree  Professional experience  Started working before 1980  Started working between 1981 and 1990  Started working between 1991 and 2000  Started working in 2001 and later  Personal familiarity with mental illness  No ill family member or no contact with ill member  Rarely sees ill member  Sees ill member several times per month  Sees ill member several times per week  Sees ill member daily  Receiving psychopharmacological treatment  Working in a public hospital  Working in a private hospital  Working in a public outpatient institution  Working in a private office  Working for a mental health insurance | Participants endorsed more overall negative stereotypes and less overall positive stereotypes. However, participants expressed less social distance.  Overall negative stereotypes were significantly negatively correlated with overall positive stereotypes. Also, overall positive stereotypes were significantly negatively correlated with social distance, and overall negative stereotypes were significantly positively correlated with social distance.  Perceiving negative attitudes towards schizophrenia in the general population was significantly negatively correlated with overall positive stereotypes, and significantly positively correlated with overall negative stereotypes and social distance.  Acceptance of psychotropic medication side-effects was significantly negatively correlated with overall positive stereotypes, and significantly positively correlated with overall negative stereotypes. This variable was not found to be significantly correlated with social distance.  Having a negative attitude towards psychotropic medication was significantly positively correlated with overall positive stereotypes, and significantly negatively correlated with overall negative stereotypes. This variable was not found to be significantly correlated with social distance.  Overall negative stereotypes were not found to be significantly related to age or any of the remaining variables.  Compared to participants that were less than 30 years old, participants that were 41 to 50 years old and participants that were 50 or older, were significantly more likely to endorse overall positive stereotypes. Being 31 to 40 years old was not found to be a significant predictor of overall positive stereotypes compared to being less than 30 years old. No other comparisons were made regarding age and overall positive stereotypes. Also, whether sex and working in a university hospital were found to be significant predictors of overall positive stereotypes was not reported.  Compared to males, female participants were significantly less likely to express social distance. Further, compared to participants that were not working in a university hospital, participants that were working in a university hospital were significantly less likely to express social distance. Whether age was found to be significant predictor of social distance was not reported.  Whether the remaining variables were found to be significantly related to overall positive stereotypes and social distance was not reported. |
| Loch et al. (2013) | Psychiatrists  (Brazil) | Structured interviews | Latent profile analysis  Multinomial logistic regression analysis | Schizophrenia (label) | Positive stereotypes compared to the general population  Healthy  Self-controlled  Reasonable  Talents  Creative  Intelligent  Gifted  Negative stereotypes compared to the general population  Dangerous  Unpredictable  Stupid  Unreliable  Strange  Bedraggled  Abnormal  Weird  Restrictiveness  Involuntary admission  Restriction of voting rights  Revocation of driver’s licenses  Social distance  Age  18-30  31-40  41-50  51-60  61 and above  Frequency of contact with a family member with a psychiatric disorder  No such family member  Rare contact  Frequent contact  Sex  Marital status  Single  Married  Previously married  Have offspring  Level of training  No doctoral degree  Doctoral or post-doctoral degree  Sought professional help for a psychiatric disorder/have been prescribed psychotropic drugs  Place of work  Public hospital  Private hospital  Public outpatient institution  University hospital  Private office  Mental health insurance | Amongst the participants three profiles were identified. Participants in the first profile (profile 1) were the most likely to believe that the positive stereotypes overall occur more often in people with schizophrenia, and the negative stereotypes overall occur less often in people with schizophrenia. Participants in this profile were also the most likely to disagree with two of the restrictiveness items, and had intermediate scores for social distance. Participants in the second profile (profile 2) were the most likely to view people with schizophrenia as being equal to the general population in terms of the stereotypes overall, and agree to involuntary admission. Participants in this profile also expressed the least amount of social distance. Participants in the final profile (profile 3) were the most likely to believe that the positive stereotypes occur less often in people with schizophrenia, and the negative stereotypes occur more. Participants in this profile were also the most likely to agree with two of the restrictiveness items, and express social distance.  In comparison to profile 1, participants in profile 2 were significantly more likely to be 18-30 years old, 31-40 years old, and 41-50 years old with 61 and above years old as the reference group. It was not found that participants in profile 2 were significantly more likely to be 51-60 years old again with 61 and above years as the reference group. Whether profile 2 was used to predict the other variables was not clear.  In comparison to profile 1, participants in profile 3 were significantly more likely to be 31-40 years old with 61 as the reference group. Profile 3 was not found to be a significant predictor of the other age groups. Participants in profile 3 were also significantly less likely to have rare contact with a family member with a psychiatric disorder, using frequent contact as a reference group. Profile 3 was not found to be a significant predictor of not having a family member with a psychiatric disorder. Further, profile 3 was not found to be a significant predictor of any of the other variables.  Variables were not predicted with either profile 2 or 3 as the respective reference group. |
| Loh et al. (2018) | GPs  Residents and physicians in internal medicine  Residents and physicians in obstetrics and gynaecology  Residents and physicians in cardiology  Residents and physicians in endocrinology  Residents and physicians in pulmonology  Medical interns |  |  |  |  | Nothing more was reported for this study as findings were not reported for GPs separately. |
| Lovi & Barr (2009) | Unspecified nurses from alcohol and other drugs units  (Australia) | Unstructured interviews | Descriptive phenomenological analysis | Drug and alcohol dependence (label) | Perceived general attitudes  Advocacy of clients in general  Ensuring clients are given more than once chance if they relapse  Providing support and understanding  People with alcohol or drug dependence are old nasty olgars  People with alcohol or drug dependence are homeless  People with alcohol or drug dependence will attack and steal from you to maintain their dependence | Participants perceived hostile attitudes in other nurses that work in the alcohol and other drugs units.  In general, it was important to the participants to be advocates for the clients. Part of this involved ensuring that the clients were given more than one chance if they relapsed, and providing them with support and understanding during this time. This is in comparison to discharging them.  One participant stated that people with alcohol or drug dependence are just people with another condition, and not old nasty olgars. This participant also stated that people that are dependent on alcohol or drugs are not necessarily homeless, and not necessarily going to attack and steal from you to maintain their dependence. |
| Lucas et al. (2005) | GPs  (England) | Semi-structured interviews | Thematic analysis | Mental illness in general (label)  Depression (label) | Causal attributions | Participants attributed mental illness to medical, social, environmental, and psychological factors. Depression in particular was attributed to uncertainty, loss of identity, greed, or boredom. |
