# Supplementary material for: The stigmatization of mental illness by mental health professionals: Scoping review and bibliometric analysis
Source: PLoS One. 2023 Jan 20;18(1):e0280739. doi: 10.1371/journal.pone.0280739 (PMC9858369; doi:10.1371/journal.pone.0280739)
Supplement: S8 Appendix — (DOCX) [file pone.0280739.s008.docx]

| **Authors (year)** | **Populations**  **(countries)** | **Research methods** | **Analytical approaches** | **Disorders** | **Variables and measures** | **Findings** |
| --- | --- | --- | --- | --- | --- | --- |
| Magliano et al. (2019) | GPs  Medical specialists  Medical  Surgical  Diagnostic/laboratory |  |  |  |  | Nothing more was reported for this study as findings were not reported for GPs separately. |
| Magliano et al. (2004) | Psychiatrists  Unspecified nurses from mental health services  Relatives of clients  (Italy) | Cross-sectional survey  A vignette was used | Kruskal-Wallis test | Schizophrenia (description) | Causal attributions  Perceived competence  Prognosis  Perceived unpredictability  It’s easy to notice that these patients have had this disorder  Segregation  Opinions on civil rights  Patients with this disorder should not get married  Patients with this disorder should not have children  The law should allow a woman to divorce from his husband as soon as possible if he suffers from this disorder  Patients with this disorder should not vote  Profession | Psychiatrists and nurses attributed schizophrenia to a range of causes with varying proportions. Heredity, stress, family conflict, and the misuse of alcohol and drugs were among the most likely causes. Magic, spirit possession, and spells, bad friendships, physical illness, and incorrect therapy were among the least likely causes.  Most of the psychiatrists and nurses believed that it is partially true that the target is competent.  Most of the psychiatrists believed that it is not true that there is little to be done for the target, and roughly half of the nurse believed this is not true.  Most psychiatrists believed that it is partially true that the target is unpredictable and easy to notice that they have a disorder. Roughly half of the nurses believed this to be partially true.  Most of the psychiatrists and nurses believed it was not true the target should be sent to an asylum.  For the opinions on civil rights, roughly half of the psychiatrists and nurses responded partially true for one item, and not true for another item, and most of the psychiatrists and nurses responded not true for a third item. For the item patients with this disorder should not get married, most of the psychiatrists responded not true, and roughly half of the nurses responded partially true.  Profession was found to have a significant impact on prognosis, it’s easy to notice that these patients have had this disorder, and perceived unpredictability. In all of these cases, nurses were either more likely to respond completely true and partially true, or more likely to respond completely true, compared to psychiatrists. Profession was not found to have a significant impact on perceived competence and segregation. This was all that was reported for the impact of profession on each measure of stigmatisation individually.  Profession was found to have a significant impact on the first three civil rights items. For all of these items, nurses were either more likely to respond completely true and partially true, or more likely to respond completely true, compared to psychiatrists. Profession was not found to have a significant impact on the item patients with this disorder should not vote. This was all that was reported for the impact of profession on each civil rights item individually.  Other relevant findings were excluded from this table as they were not reported for mental health professionals separately. |
| Magliano et al. (2004) | Psychiatrists  Psychologists  Social workers  Occupational therapists  Unspecified nurses working in a mental health centre  Sociologists  Auxiliary and administrative personnel  General population |  |  |  |  | Nothing more was reported for this study as findings were not reported for mental health professionals separately. |
| Maier et al. (2015) | Mental health professionals from the following fields specialising in psychotraumatology  Medicine  Psychology  Social work  Other unspecified mental health professionals specialising in psychotraumatology  Psychiatry residents  (Switzerland) | Experiment  Vignettes were used | Between-groups ANOVA | Depression (description)  PTSD (description) | Likability  Perceived dependency  Perceived autonomy  Perceived personal responsibility  Expected quality of therapeutic relationship  Prognosis with and without professional help | Across the measures, mental health professionals mostly expressed less stigmatisation towards both mental disorders. However, both mental disorders were perceived as less autonomous, and depression was blamed more. Also, although the mental health professionals gave a good prognosis in the context of professional help, a bad prognosis was given when the targets were not receiving professional help. This was not examined with inferential statistics.  Compared to depression, mental health professionals perceived PTSD as significantly more likable and autonomous, and significantly less dependent and blameworthy. However, PTSD was given a significantly worse prognosis than depression in the context of not receiving professional help. No significant differences were found between PTSD and depression for expected quality of therapeutic relationship and prognosis with professional help.  Other relevant findings were excluded from this table as they were not reported for mental health professionals separately. |
| Malcolm et al. (1977) | Family physicians  Family physician residents |  |  |  |  | Nothing more was reported for this study as findings were not reported for family physicians separately. |
| Manis et al. (1963) | Staff members from the following fields  Psychology  Psychiatry  Social work  (USA) | Cross-sectional survey | - | Mental illness in general (label) | A measure of beliefs about mental illness (most items were not specified)  Will power is the basis of personal adjustment  Pleasant preoccupation is the basis of mental health  The maintenance of mental health is through a dependence on strong persons in the environment  Mental troubles are caused by physical exhaustion, and financial and social problems  Persons become more susceptible to emotional disorders as they grow older  Mental disorder is brought on by organic factors  Moralism (e.g., if a person becomes mentally ill it is because he did not live right)  The mentally ill look and act differently  There is little that can be done to cure a mental disorder | Participants disagreed more with every belief. The only exception to this was participants agreed slightly more with the maintenance of mental health is through a dependence on strong persons in the environment. |
| Mannarini et al. (2020) | Psychiatric nurses  Psychiatrists  Psychologists  Social workers  Educators  Psychology students  Psychiatric patients and their relatives |  |  |  |  | Nothing more was reported for this study as findings were not reported for mental health professionals separately. |
| Manning et al. (2020) | Psychologists  Social workers  Occupational therapists  Unspecified doctors from mental health services  Unspecified nurses from mental health services  Support workers  Other unspecified providers from mental health services |  |  |  |  | Nothing more was reported for this study as findings were not reported for mental health professionals separately. |
| Markham & Tower (2003) | Mental health nurses  (England) | Experiment | Withing-groups ANOVA  Correlation analysis | BPD (label)  Schizophrenia (label)  Depression (label) | Attributions of challenging behaviours (e.g., acting in a violent manner, setting off a fire alarm when there was no fire, failing to attend a planned therapeutic activity or refusing to carry out a request from staff)  Internality  Stability  Globality  Control of cause  Control of event  Sympathy  Prognosis  Personal experience with the target disorders  Positive  Negative | Attributions were unable to be fully interpreted with the information provided.  Challenging behaviour was perceived as the most internal for schizophrenia, followed by BPD, followed closely by depression. BPD was not found to be significantly different to either schizophrenia or depression. Whether there was a statistically significant difference between schizophrenia and depression was not reported.  Challenging behaviour was perceived as significantly more stable for BPD compared to schizophrenia and depression. Challenging behaviour was perceived as slightly more stable for schizophrenia compared to depression. Whether this difference was statistically significant was not reported.  Mental disorder was not found to have a significant impact on globality.  Control of cause and event were attributed to BPD significantly more compared to schizophrenia and depression. Also, control of cause and event were attributed to depression more than schizophrenia. Whether these differences were statistically significant was not reported.  Participants expressed significantly less sympathy and a significantly worse prognosis for BPD than schizophrenia and depression. Schizophrenia elicited more sympathy and a worse prognosis than depression. Whether these differences were statistically significant was not reported.  For all mental disorders, control of the cause and event were significantly negatively correlated with sympathy.  For BPD, personal experience was not found to be significantly associated with attributions of challenging behaviour. The relationship between these variables was not assessed for the other mental disorders. For all the mental disorders, a negative personal experience was significantly positively correlated with a poor prognosis. |
| Martensson et al. (2014) | Mental health nurses  Assistant nurses  Nurses  Hospital orderlies |  |  |  |  | Nothing more was reported for this study as findings were not reported for mental health nurses separately. |
| Masland et al. (2018) | Psychiatrists  Social workers  Psychologists  Counsellors  Unspecified nurses  Other unspecified health professionals |  |  |  |  | Nothing more was reported for this study as findings were not reported for mental health professionals separately. |
| McGillion et al. (2000) | GPs  (England) | Cross-sectional survey | - | Drug misuse (label)  Other unspecified patients (label) | Working with drug misusers is more stressful than any other patient  Most drug misusers are unreliable and disruptive  Most patients presenting with a drug problem are not serious about needing help  Most drug users are unresponsive to help with their drug problem | Most participants believed that working with drug misusers is more stressful than any other patient.  For the reaming measures, roughly half of the participants expressed agreement. |
| McGovern et al. (1986) | Clinical psychologists  Psychology interns |  |  |  |  | Nothing more was reported for this study as findings were not reported for clinical psychologists separately. |
| McKeown et al. (2003) | GPs  (Scotland) | Semi-structured telephone interviews | Thematic analysis | Drug misusers (label) | Causal attributions  Endorsed and perceived willingness to provide general medical care/treatment  Behaviour, motivation, and commitment of the patient  Negative viewpoints in general  Drug misusers are difficult and intentionally misleading  Perceived dangerousness | Drug misuse was attributed to coping with societal issues (e.g., poverty, unemployment) rather than medical factors.  Most of the participants were comfortable providing drug misusers with general medical care. One participant stated that they treat drug misusers like anyone else. However, participants acknowledged that not all GPs treat drug misusers in this way.  It was suggested that the behaviour, motivation, and commitment of the patient is related to willingness to provide treatment. More specifically, it was suggested that verbal and physical aggression is related to less willingness to provide treatment. Nothing more was reported about motivation and commitment.  The majority of participants had some negative viewpoints regarding drug misusers. One participant perceived drug misusers as difficult and intentionally misleading. Also, several participants were concerned about drug misusers becoming violent. |
| Mclntyre & Schwartz (1998) | Psychotherapists  (USA) | Cross-sectional survey  Audiotaped interviews presenting cases of BPD or major depression were used | Factorial ANOVA  Correlation analysis | BPD (presentation)  Major depression (presentation) | IMI (items were not specified)  Dominant (perceptions of exhibitionism, attention seeking, and hunger for approval from others)  Hostile (tendencies to criticize, ridicule, or punish another individual; perceptions of doubt concerning the attitudes and intentions of others; and emotional detachment to minimize emotional investment)  Submissive (perceptions such as a willingness to accept blame, a tendency to belittle oneself, passiveness, the appearance of helplessness, and a preference of yielding responsibility)  Friendly (feelings of agreeableness, cooperativeness, consideration, sympathy, fondness, and warmth)  SAS (items were not specified and only factors relevant to stigmatisation were included in this table)  Difficulty (a belief that the target is a significant stressor and feelings of anxiety)  Sex  Years of professional experience | Overall, participants endorsed the IMI factors between not at all and somewhat for both mental disorders.  The level of difficulty perceived by the participants was unable to be fully interpreted with the information provided.  BPD elicited significantly more dominant perceptions and hostile reactions than major depression, and major depression elicited significantly more submissive perceptions and friendly reactions than BPD. No significant difference was found between BPD and major depression for difficulty.  Sex was not found to have a significant impact on stigmatisation, and the interaction between sex and mental disorder was not found to be significant for any of the factors.  Years of professional experience was significantly negatively correlated with dominant perceptions and difficulty for depression. No other significant correlations between years of professional experience and stigmatisation were found. |
| Meza et al. (2001) | Counsellors  Social workers  Psychiatrists  Other unspecified physicians from drug and alcohol treatment facilities  (Canada) | Cross-sectional survey | Between-groups ANOVA | Problem drinkers (label) | In alcohol-dependent individuals, drinking is explainable on the grounds of self-medication of distressing psychiatric or psychological symptoms  A significant number of problem drinkers can give up alcohol without any professional treatment or self-help groups  Profession | For the measure in alcohol-dependent individuals, drinking is explainable on the grounds of self-medication of distressing psychiatric or psychological symptoms, counsellors and social workers agreed more. However, psychiatrists and other physicians disagreed more. Profession was found to have significant impact on this measure. Social workers agreed with this measure significantly more than all other professions. Counsellors agreed significantly more than other physicians, but were not found to be significantly different to psychiatrists. Psychiatrists were not found to be significantly different to other physicians.  Participants agreed more that a significant number of problem drinkers can give up alcohol without any professional treatment or self-help groups. Profession was not found to have a significant impact on this measure. |
| Miller & Davenport (1996) | Unspecified nurses from psychiatric units  (USA) | Quasi-experiment  An intervention was used | Between-groups ANOVA  Correlation analysis | BPD (label) | A measure of attitudes towards BPD (items were not specified)  Whether the participants had completed an educational program on BPD  Knowledge about BPD  Source of previous knowledge about BPD (the sources were not specified)  Current position (levels were not specified)  Years in current position  Years in psychiatric nursing  Age  Formal education level (it was not clear if this meant general education or education in nursing)  Most recent learning about BPD  Most recent contact with a patient with BPD | At pre-test, both the educational program group and the control group had more negative attitudes.  At post-test, the control group had more negative attitudes and the educational program group had a mix of positive and negative attitudes. Controlling for pre-test scores, the educational program group had significantly more positive attitudes than the control group at post-test.  At both pre-test and post-test, there was a significant positive correlation between knowledge about BPD and positive attitudes.  Source of previous knowledge about BPD, current position, years in current position, years in psychiatric nursing, age, formal education level, most recent learning about BPD, and most recent contact with a patient with BPD were not found to be significantly correlated with attitudes. |
| Mittal et al. (2014) | Mental health nurses  Psychiatrists  Psychologists  Primary care physicians  Primary care nurses |  |  |  |  | Nothing more was reported for this study as findings were not reported for mental health professionals separately. |
| Mittal et al. (2016) | Mental health nurses  Psychiatrists  Psychologists  Primary care physicians  Primary care nurses |  |  |  |  | Nothing more was reported for this study as findings were not reported for mental health professionals separately. |
| Mittal et al. (2019) | Primary care physicians  Primary care nurses |  |  |  |  | Nothing more was reported for this study as findings were not reported for primary care physicians separately. |
| Modgill et al. (2014) | Social workers  Psychologists  Psychiatrists  Family physicians  Anaesthetists  Surgeons  Emergency physicians  Other unspecified physicians  Unspecified nurses  Medical students  Nurse students  Occupational therapy students  Pharmacy students  Unspecified non-medical staff  Other unspecified professionals or students  (Canada) | Cross-sectional and longitudinal survey  Interventions were used | Between-groups ANOVA  Paired samples t-test | Mental illness in general (label) | OMS-HC (only factors relevant to endorsed provider-based stigmatisation were included in this table)  Negative attitudes  Preference for greater social distance  Profession | Social workers, psychologists, psychiatrists, and family physicians expressed less negative attitudes and less of a preference for social distance.  For negative attitudes, family physicians expressed the most stigmatisation, followed by social workers, closely followed by psychologists, closely followed by psychiatrists. Psychiatrists expressed significantly less negative attitudes than family physicians. The other profession differences were not fully examined with inferential statistics. For social distance, psychologists expressed the most stigmatisation, closely followed by psychiatrists, closely followed by family physicians, closely followed by social workers. Psychiatrists expressed significantly less social distance than family physicians, and no significant difference was found between psychologists and social workers. The other profession differences were either not fully examined with inferential statistics, or were not examined with inferential statistics separately for mental health professionals.  Social workers participated in an anti-stigma workshop that was about BPD and dialectical behaviour therapy. Negative attitudes and social distance were not found to differ significantly between before and after the intervention.  Other relevant findings were excluded from this table as they were not reported for mental health professionals separately. |
| Mohamed-Kaloo & Laher (2014) | GPs  (South Africa) | Semi-structured telephone interviews | Thematic analysis | Mental illness in general (label)  Anxiety (label)  Depression (label)  Stress disorders (label) | Causal attributions | Participants believe that mental illness is caused by organic factors, and stress (including financial difficulties and family conflict) and lifestyle factors. The latter two factors were believed to impact anxiety, depression, and stress disorders in particular. |
| Molina-Mula et al. (2018) | Mental health nurses  Emergency nurses |  |  |  |  | Nothing more was reported for this study as findings were not reported for mental health nurses separately. |
| Moodley-Kunnie (1988) | Psychiatric nurses  Unspecified non-psychiatric nurses  Psychiatric nursing students  Non-psychiatric nursing students |  |  |  |  | Nothing more was reported for this study as findings were not reported for psychiatric nurses separately. |
| Morant (2006) | Psychiatrists  Clinical psychologists  Psychiatric nurses  Social workers  Occupational therapists  A psychotherapist  An art therapist  Case mangers  Movement therapists  A community programme worker  A community centre receptionist  (England, France) | Semi-structured interviews | Thematic analysis | Mental illness in general (label)  Neurosis (label) | Causal attributions | One French psychiatrist reported that treating neurosis involved changing the personality of an individual. Another French psychiatrist alluded to psychoanalytic processes as an explanation for mental illness.  Other relevant findings were excluded from this table as they were not reported for mental health professionals separately. |
| Morrison & Becker (1975) | Professionals in the following fields that provide psychiatric services  Psychology  Psychiatry  Social work  Nursing  (USA) | Longitudinal survey and structured interviews with open-ended questions  An intervention was used | Repeated measures t-test | Mental illness in general (label) | A measure of agreement with the medical model called the CAQ (only the following items were specified)  Mental hospitals should be abolished  Mental patients are not able to fool a psychiatrist  Only the following interview questions were specified (these questions were derived from the CAQ)  What would you say are common causes of mental health problems?  How do you feel about mental hospitals? | Overall, participants endorsed more of a psychosocial model than a medical model.  Compared to pre-test scores, participants endorsed the psychosocial model significantly more after a seminar on theoretical and operational approaches to mental illness. No significant differences were found between the three post-test time points.  Participants with scores that changed substantially from pre to post-test were interviewed. Social, familial, and environmental factors were stressed, and participants believed that hospitals contribute to the depersonalization and dehumanization of people with mental illness. |
| Morrison & Hanson (1978) | Psychologists  Social workers  Psychiatrists  Psychiatric nurses  (USA) | Cross-sectional survey | Two proportion z-test | Mental illness in general (label) | A subset of items from the CAQ  I believe that mental illness is an illness like any other  People have been duped or fooled into believing that there is such a thing as mental illness  Psychiatrists and psychologists almost always can tell a mentally ill person from a normal person  Mostly women, rather than men, end up being diagnosed as schizophrenic and psychotic  There is really no such thing as mental illness, just people with problems  Mental hospitals should be abolished  Profession | Compared to the medical model, roughly half of the psychologists endorsed a psychosocial orientation overall, whereas most of the other professions endorsed a medical orientation overall (not sure was an available option).  Most of the participants did not agree that mental hospitals should be abolished. Psychologists were the most likely to agree with this statement, followed by psychiatrists, followed by nurses. No social workers agreed that mental hospitals should be abolished. These differences were not examined with inferential statistics (not sure was an available option).  Psychologists were significantly more likely to be oriented towards the psychosocial view overall, compared to the other professions. For the remaining three professions, social workers were the most likely to be oriented towards the psychosocial view overall, followed by nurses, followed by psychiatrists. Differences between these professions were not examined with inferential statistics. |
| Morrison & Nevid (1976) | Psychiatrists  Psychiatric nurses  Psychologists  Social workers  (USA) | Cross-sectional survey | Independent samples t-test | Mental illness in general (label) | CAQ (items were not specified)  Profession | Compared to the medical model, participants agreed more with a psychosocial orientation overall.  Psychologists agreed with a psychosocial orientation significantly more than the other professions, and social workers agreed with a psychosocial orientation significantly more than nurses and psychiatrists. Nurses agreed slightly more with a psychosocial orientation than psychiatrists, but this was not examined with inferential statistics. |
| Morrison et al. (1977) | Social workers  Counsellors  Other unspecified mental health professionals  Public health nurses from a county health department and alcohol rehabilitation program  Lawyers  Outreach workers  Other unspecified health professionals  (USA) | Cross-sectional survey | - | Mental illness in general (label) | A revised version of the CAQ called the CAQ-B (items were not specified) | In comparison to a medical orientation, the unspecified mental health professionals agreed more with a psychosocial orientation.  Other relevant findings were excluded from this table as they were not reported for mental health professionals separately. |
| Morrison et al. (1979) | Social workers  (USA) | Cross-sectional survey | - | Mental illness in general (label) | CAQ-B (only the following items were specified)  There is no such thing as mental illness, just people with problems  There are some people who clearly suffer from schizophrenia | Participants were mostly unsure about whether they endorsed a medical or psychosocial orientation. |
| Morrison et al. (1976) | Psychiatric nurses  (USA) | Cross-sectional survey | - | Mental illness in general (label) | CAQ (only the following items were specified)  People have been duped or fooled into believing that there is such a thing as mental illness  Mental hospitals should be abolished | Participants agreed slightly more with a psychosocial orientation overall, compared to a medical orientation. |
| Morgan (2016) | Mental health nurses  Palliative care/hospice nurses  Several other non-mental health nurses  (USA) | Semi-structured interviews | Thematic analysis | Mental illness in general (label) | People with mental illness refuse to take medication | One mental health nurse expressed that patients with mental illness will always refuse medication.  Other relevant findings were excluded from this table as they were not reported for mental health nurses separately. |
| Mosaku & Wallymahmed (2017) | Unspecified doctors working in primary care centres  Unspecified nurses  Health assistants  Community health officers  Community extension workers |  |  |  |  | Nothing more was reported for this study as findings were not reported for doctors separately. |
| Moss & Davidson (1982) | Social workers  Adherence counsellors  Teachers  Psychology students |  |  |  |  | Nothing more was reported for this study as findings were not reported for social workers separately. |
| Motteli et al. (2019) | Psychologists  Social workers  Unspecified physicians from a psychiatric facility  Unspecified nurses from a psychiatric facility  Occupational therapists  Other unspecified therapists |  |  |  |  | Nothing more was reported for this study as findings were not reported for mental health professionals separately. |
| Muga & Jenkins (2008) | Psychiatric nurses  General nurses  Unspecified doctors  Unspecified clinical officers |  |  |  |  | Nothing more was reported for this study as findings were not reported for psychiatric nurses separately. |
| Mulango et al. (2018) | GPs  Social workers  Pharmacy attendants  Primary care nurses  Nurse assistants |  |  |  |  | Nothing more was reported for this study as findings were not reported for mental health professionals separately. |
| Munro & Baker (2007) | Mental health nurses  Healthcare assistants |  |  |  |  | Nothing more was reported for this study as findings were not reported for mental health nurses separately. |
| Murray et al. (2006) | GPs  Counsellors (two of which were trained as clinical psychologists)  Practice nurses  (England) | Unstructured interviews | Thematic analysis | Depression (label) | Causal attributions | GPs and counsellors believed that depression should not be regarded as a normal reaction to getting old. Also, GPs alluded to social (e.g., loneliness) and socioeconomic factors as causes of depression.  Other relevant findings were excluded from this table as they were not reported for mental health professionals separately. |
| Muyambi et al. (2021) | Social workers  General nurses  Peer support workers |  |  |  |  | Nothing more was reported for this study as findings were not reported for social workers separately. |
| Najavits (2002) | Social work professionals  Counsellors  Psychology professionals  Psychiatrists  A GP  Nursing professionals from mental health and substance abuse setting, and forensic practices and homeless shelters  Probation officers  Business administrators  Other unspecified professionals |  |  |  |  | Nothing more was reported for this study as findings were not reported for mental health professionals separately. |
| Ndetei et al. (2011) | Occupational therapists  Unspecified doctors from primary care and other health care facilities  Medical interns  Unspecified clinical officers  Nurses  Pharmacists  Dentists  Rehabilitation workers  Physiotherapists  Laboratory medicine workers  Medical students  Pharmacy students  Dentistry students  Administrator students  Other unspecified students |  |  |  |  | Nothing more was reported for this study as findings were not reported for mental health professionals separately. |
| Newhill & Korr (2004) | Social workers  (USA) | Cross-sectional survey with closed and open-ended questions | Content analysis  Independent samples t-test | Mental illness in general (label) | Avoidance  Dislike working with mental illness  Discomfort working with mental illness  Frustration  Prognosis  Perceived dangerousness  Prefer not to treat mental illness  Primary employment  Community mental health  Private practice | A small proportion of participants reported that they no longer work with mental illness because they don’t like working with mental illness, they feel uncomfortable working with mental illness, it is frustrating working with mental illness, and they are unlikely to make an impact. Another small proportion of participants stated that they no longer work with mental illness due to fear of violence.  Most participants preferred not to treat mental illness.  Participants from private practice expressed significantly more that they prefer not to treat mental illness, compared to participants from community mental health. |
| Newton-Howes et al. (2008) | Mental health professionals from the following fields  Medicine  Nursing  Social work  Other unspecified mental health professionals  (England) | Cross-sectional survey  Information was provided on patients (it was not clear what form this was presented in) | Mann-Whitney *U-*test | Personality disorder (a label was used and it was not clear how the remaining information was presented)  Schizophrenia (it was not clear how this was presented)  Depression (it was not clear how this was presented)  Anxiety (it was not clear how this was presented)  Alcohol dependence (it was not clear how this was presented)  Drug dependence (it was not clear how this was presented)  Any psychiatric diagnosis (it was not clear what this meant and how it was presented) | Perceived difficulty  Perceived instability  Perceived aggressiveness  Perceived engagement  Perceived compliance  Personality disorder presentation  Label  No label | Stigmatisation scores were unable to be fully interpreted with the information provided.  Compared to the other mental disorders, personality disorder was perceived as significantly more difficult and aggressive, and significantly less compliant. Mental disorder was not found to have a significant impact on the other measures of stigmatisation. Mental disorders other than personality disorder were not compared.  Compared to personality disorder without a label, the presence of a personality disorder label elicited significantly more perceptions of difficulty, instability, and aggression. Personality disorder presentation was not found to have a significant impact on the other measures of stigmatisation. |
| Ngako et al. (2012) | Psychiatric nurses  (South Africa) | Focus groups | Thematic analysis | Mental illness in general (label) | Perceptions of safety  Fear  Helplessness  Frustration  Anger  Ambivalence  Guilt  Demotivation  Perceived unpredictability  Perception that you get nothing in return for working with mental illness  Desire to develop general positive attitudes  Quality of nursing care | Participants expressed that working with mental illness involves entering an unsafe world.  Participants expressed fear, helplessness, frustration, anger, ambivalence, guilt, and a feeling of demotivation towards people with mental illness.  One participant perceived people with mental illness as unpredictable, and stated that this is where the fear comes from.  Another participant expressed that despite the hard work put in, you get nothing in return.  Participants expressed a desire for developing positive attitudes in general towards people with mental illness. Participants also suggested that this would increase the quality of nursing care for people with mental illness. |
| Nordt et al. (2006) | Psychiatrists  Unspecified nurses from psychiatric facilities  Psychologists  Social workers  Vocational workers  Physiotherapists  Other unspecified professionals from psychiatric facilities  General population  (Switzerland) | Structured telephone interviews and experiment  Vignettes were used | Between-groups ANOVA | Mental illness in general (label)  Major depression (description)  Schizophrenia (description) | Stereotypes  Dangerous  Unpredictable  Stupid  Bedraggled  Abnormal  Unreliable  Weird  Reasonable  Self-controlled  Healthy  Restrictions  What do you think: should a woman who had suffered severely from a mental illness have an abortion in the case of a pregnancy?  Do you approve of the right to vote and to run for office for somebody who had suffered severely from a mental illness?  What do you think: should somebody who is severely mentally ill have her/his driver’s license revoked?  What do you think: should somebody be admitted to a psychiatric hospital even against his/her will and if needed retained, or should a person under no circumstances be compulsorily admitted to a psychiatric hospital?  Social distance towards depression and schizophrenia  Profession  Sex | Psychiatrists, nurses, and psychologists expressed more overall stigmatisation for stereotyping. Although most of these participants agreed with involuntary admission, most of these participants did not agree with the other restrictions for people with mental illness. The only exception to this was roughly half of the nurses agreed with driver’s licence revocation. For social distance, the responses of the psychiatrists, nurses, and psychologists were different for the two mental disorders. For major depression, these participants expressed less social distance. For schizophrenia psychologists and nurses expressed roughly neutral responses, and psychiatrists expressed more social distance.  Psychiatrists, nurses, and psychologists expressed significantly more social distance towards schizophrenia than major depression.  For stereotypes, psychiatrists expressed the most overall stigmatisation towards mental illness compared to nurses, followed by psychologists. Psychiatrists expressed significantly more overall stigmatisation for stereotyping mental illness than nurses and psychologists. The difference between nurses and psychologists was not examined with inferential statistics. For driver’s licence revocation, nurses were the most likely to agree, followed by psychiatrists, followed by psychologists. These differences were not examined with inferential statistics, and profession was not found to have a significant impact on the remaining restrictions items. For schizophrenia, psychiatrists expressed the most social distance, followed by psychologists, closely followed by nurses. However, for major depression, nurses expressed the most social distance, followed by psychiatrists, followed by psychologists. Differences between these professions for social distance were not examined with inferential statistics.  Sex was not found to have a significant impact on overall stereotyping for the psychiatrists, nurses, and psychologists.  Other relevant findings were excluded from this table as they were not reported for mental health professionals separately. |
| Norton et al. (2011) | Family physicians  (France) | Cross-sectional survey | Multiple standardised regression analysis | Depression (label) | DAQ (only relevant items and factors were included in this table)  Origins of depression and its amenability to change  The majority of depression seen in general practice originates from patients’ recent misfortunes  It is possible to distinguish two main groups of depression: one psychological in origin and the other caused by biochemical mechanisms  Becoming depressed is a way that people with poor stamina deal with life’s difficulties  Depressed patients are more likely to have experienced deprivation early in life than other people  Depression reflects a characteristic response that is not amenable to change  Becoming depressed is a natural part being old  An underlying biochemical abnormality forms the basis of severe cases of depression  Most depressive disorders seen in general practice improve without medication  Working with depressed patients can be difficult  Desire for future training in mental health  Received training in mental health  Type of training received in mental health  Two-day continuing medical education seminar  Evening continuing medical education seminar  Hospital psychiatry  Balint group  Personal training  Age (control variable)  Sex (control variable) | Participants disagreed more with the majority of the DAQ items. The only exceptions to this were participants agreed more that the majority of depression seen in general practice originates from patients’ recent misfortunes, and an underlying biochemical abnormality forms the basis of severe cases of depression.  Controlling for age and sex, none of the non-stigmatisation variables were found to be significant predictors of origins of depression and its amenability to change overall. Also, the interaction between having a desire for future training in mental health and having received training in mental health was not found to be a significant predictor of origins of depression and its amenability to change overall. |
| Nunnally (1957) | Psychologists  Psychiatrists  General population  (USA) | Cross-sectional survey | - | Mental illness in general (label) | Causal attributions  People with mental illness look and act different to normal people  Prognosis  People with mental illness speak in words that can be understood  Profession | Mental health professionals disagreed more that mental illness reflects a lack of will power, mental health is maintained by having pleasant thoughts, older people are more susceptible to mental illness, and mental illness is caused by poor diet and diseases of the nervous system. Mental health professionals responded roughly neutrally to good mental health being maintained through a dependence on strong persons, and mental illness being caused by a lack of affection in childhood. The causal attribution regarding external factors compared to personality factors was unable to be interpreted with the information provided.  Mental health professionals disagreed more that people with mental illness look and act different to normal people, and that they are unlikely to recover. Mental health professionals also agreed more that people with mental illness speak in words that can be understood.  It was reported that psychologists and psychiatrists responded similarly. This was not examined with inferential statistics.  Other relevant findings were excluded from this table as they were not reported for mental health professionals separately. |
| Nutt et al. (2016) | Psychologists  Psychiatrists  Social workers  GPs  Unspecified nurses from addiction/mental health services and general practice  Occupational therapists  Health or social care assistants  A nursing student  An office manager  An unspecified senior practitioner  (Scotland) | Cross-sectional survey | - | Depression (label)  Alcohol misuse (label)  Drug misuse (label)  Comorbid substance misuse and depression (label) | MCRS  I prefer not to work with patients like this  Patients like this irritate me  I enjoy giving extra time to patients like this  Patients like this are particularly difficult for me to work with  Working with patients like this is satisfying  I feel especially compassionate toward patients like this  I wouldn’t mind getting up on call nights to care for patients like this  I can usually find something that helps patients like this feel better  There is little I can do to help patients like this  Insurance plans should cover patients like this to the same degree that they  cover patients with other conditions  Treating patients like this is a waste of medical dollars  Profession | Psychologists, psychiatrists, social workers, and GPs expressed more overall positive regard towards all of the mental disorders. The only exception to this was GPs expressed slightly less overall positive regard towards drug misuse.  For psychologists, psychiatrists, social workers, and GPs, a hierarchical pattern emerged in which participants stigmatised the different mental disorders to varying degrees. This pattern was different for each of these professions. The only consistent finding across these professions was depression elicited more positive regard overall than comorbid substance misuse and depression. Differences between the mental disorders were not examined with inferential statistics.  For psychologists, psychiatrists, social workers, and GPs, the only consistent finding across the mental disorders was GPs consistently expressed less overall positive regard than psychiatrists and social workers. Differences between these professions were not examined with inferential statistics separately to the irrelevant subsets of the sample.  Other relevant findings were excluded from this table as they were not reported for mental health professionals separately. |
