# Supplementary material for: The stigmatization of mental illness by mental health professionals: Scoping review and bibliometric analysis
Source: PLoS One. 2023 Jan 20;18(1):e0280739. doi: 10.1371/journal.pone.0280739 (PMC9858369; doi:10.1371/journal.pone.0280739)
Supplement: S9 Appendix — (DOCX) [file pone.0280739.s009.docx]

| **Authors (year)** | **Populations**  **(countries)** | **Research methods** | **Analytical approaches** | **Disorders** | **Variables and measures** | **Findings** |
| --- | --- | --- | --- | --- | --- | --- |
| Ogden et al. (1999) | GPs  (England) | Cross-sectional survey | It was not clear what statistical analysis was used | Depression (label) | Causal attributions  Lived experience with depression | Participants agreed the most with psychological causes of depression (e.g., unhappy childhood), followed by medical causes (e.g., hormones), followed by external causes (e.g., time of the year).  Lived experience with depression was not found to have a significant impact on causal attributions. |
| Ola et al. (2014) | Primary care physicians  (Nigeria) | Cross-sectional survey | - | Depression (label) | DAQ (only items relevant to stigmatisation were included in this table)  Becoming depressed is a way that people with poor stamina deal with life’s difficulties  The majority of depression seen in general practice originates from patients’ recent misfortunes  Becoming depressed is a natural part of old age  It is possible to distinguish two main groups of depression: one psychological in origin and the other caused by biochemical abnormalities  Depressed patients are more likely to have experienced deprivation in early life than other people  An underlying biochemical abnormality forms the basis of severe cases of depression  Most depressive disorders seen in general practice improve without medication  Depression reflects a characteristic response that is not amenable to change | Most of the participants agreed that becoming depressed is a way that people with poor stamina deal with life’s difficulties, and the majority of depression seen in general practice originates from patients’ recent misfortunes. In comparison, most participants disagreed that becoming depressed is a natural part of old age, and it is possible to distinguish two main groups of depression: one psychological in origin and the other caused by biochemical abnormalities. Also, roughly half of the participants disagreed that depressed patients are more likely to have experienced deprivation in early life than other people, and an underlying biochemical abnormality forms the basis of severe cases of depression.  Most of the participants agreed that most depressive disorders seen in general practice improve without medication. However, most participants also agreed that depression reflects a characteristic response that is not amenable to change. |
| Ori et al. (2020) | Psychiatrists  Trainee psychiatrists |  |  |  |  | Nothing more was reported for this study as findings were not reported for psychiatrists separately. |
| Ozer et al. (2017) | Psychiatrists  Psychiatric nurses  Other unspecified mental health workers  Paramedics  (Turkey) | Cross-sectional survey | - | Mental illness in general (label) | BMI scale (items were not specified)  Perceived dangerousness  Perceived incurability and disturbance in interpersonal relationships  Shame  Self-reported use of stigmatising language (items were not specified clearly) | Psychiatrists expressed less stigmatisation overall on the BMI scale (individual factor scores could not be interpreted with the information provided).  The self-reported use of stigmatising language score for psychiatrists was unable to be interpreted with the information provided.  Other relevant findings were excluded from this table as they were not reported for mental health professionals separately. |
| Pace et al. (2011) | Family physicians  Obstetricians  Gynaecologists  Internists  Paediatricians |  |  |  |  | Nothing more was reported for this study as findings were not reported for family physicians separately. |
| Payne et al. (2011) | Primary care physicians  Primary care nurses |  |  |  |  | Nothing more was reported for this study as findings were not reported for primary care physicians separately. |
| Pepper et al. (2007) | Primary care physicians  Primary care nurses  Physician assistants |  |  |  |  | Nothing more was reported for this study as findings were not reported for primary care physicians separately. |
| Peris et al. (2008) | Clinical psychologists  Social workers  Counsellors  Psychiatrists  Other unspecified mental health professionals  Clinical psychology students  (USA) | Cross-sectional survey  IAT | Between-groups ANOVA  One of the analyses was not clear | Mental illness in general (label) | Implicit general attitudes (only example categories were specified)  Good (e.g., wonderful, joyful)  Bad (e.g., terrible, awful)  Explicit general attitudes  Explicit stereotypes  Blameworthy/innocent  Helpless/competent  Profession | Mental health professionals expressed slightly more positive implicit general attitudes towards mental illness.  Clinical psychologists expressed more positive implicit general attitudes towards mental illness than the other unspecified mental health professionals, followed by social workers and counsellors. Although profession was found to have a significant impact on implicit general attitudes, differences between the professions were not examined with multiple comparisons.  Profession was not found to have a significant impact on overall explicit stigmatisation.  Other relevant findings were excluded from this table as they were not reported for mental health professionals separately. |
| Pfeffer & Erdal (2015) | Unspecified doctors from a mental health unit  Unspecified clinical officers from a mental health unit  Unspecified nurses from a mental health unit  Village health team members  A range of mental health trainees  General population |  |  |  |  | Nothing more was reported for this study as findings were not reported for mental health professionals separately. |
| Pinikahanaet et al. (2002) | Unspecified nurses from a substance use and mental illness treatment facility  Psychologists  Social workers  Psychiatrists  Occupational therapists  Other unspecified mental health professionals  (Australia) | Cross-sectional survey | - | Mental illness in general (label)  Drug and alcohol dependence (labels) | A measure of attitudes towards drugs and alcohol based on the SAAS (only items relevant to stigmatisation were included in this table)  Cannabis use leads to mental illness  Recreational drug use leads to drug misuse  Alcohol dependence is associated with a weak will  All heroin use leads to dependence  Weekend users will progress to drug misuse  Heroin is so addictive that no one can really recover once he/she becomes dependent  Drug dependence is a treatable illness  Alcohol dependence is a treatable illness  An alcohol or drug dependent person who has relapsed several times probably cannot be treated  Most alcohol and other drug dependent persons are unpleasant to work with as patients  Angry confrontation is necessary in the treatment of alcohol and other drug dependent people  Chronic alcohol dependent people who refuse treatment should be legally committed to long term treatment | For the causal attribution items, most participants disagreed.  For the remaining items, most participants expressed a lack of stigmatisation. |
| Potamianos et al. (1985) | GPs  Psychiatrists  Psychiatric nurses  Other unspecified physicians  Other unspecified nurses  Patients with alcohol related problems  (England) | Cross-sectional survey | - | Problem drinker (label) | A measure of attitudes towards problem drinkers  Sociable-solitary  Confident-not confident  Law abiding-law breaking  Realist-escapist  Happy-unhappy  Attractive-unattractive  Healthy-ill  Conscientious at work-not conscientious at work  Happy childhood-unhappy childhood  Responsible-irresponsible  Unlikely to become dependent on other drugs-likely to become dependent on other drugs  Responsible for own condition-not responsible for own condition | GPs stigmatised problem drinkers more overall.  Other relevant findings were excluded from this table as they were not reported for mental health professionals separately. |
| Pranckeviciene et al. (2018) | Psychologists  Social workers  Psychology students  Social work students  (Lithuania) | Cross-sectional survey | ANCOVA | Mental illness in general (label) | Social distance  Profession  Years of professional experience  Level of training  Master’s degree  No master’s degree  Social desirability  Conscious  Unconscious  Personal familiarity with mental illness  Visited a psychiatrist or psychologist due to personal problems  Have a family member/friend with mental illness | For mental health professionals with six to ten years of experience and more than ten years of experience, psychologists expressed more social distance, and social workers expressed less social distance. However, for mental health professionals with up to five years of experience, psychologists expressed less social distance and social workers expressed more social distance. Differences between the professions were not examined with inferential statistics for the mental health professionals separately.  Controlling for social desirability and personal familiarity with mental illness, psychologists with less than five years of experience expressed significantly less social distance than psychologists with more than ten years of experience, and this was reversed for social workers. For social workers, social distance was highest for participants with up to five years of experience, and was roughly the same for participants with six to ten years of experience, and more than ten years of experience. For psychologists, social distance was lowest for participants with up to five years of experience, was higher for participants with six to ten years of experience, and was highest for participants with more than ten years of experience. These differences were not examined with inferential statistics, and the interaction between profession and years of professional experience was not examined with inferential statistics for mental health professionals separately.  Controlling for social desirability and personal familiarity with mental illness, level of training was not found to have a significant impact on social distance for social workers. The impact of level of training on social distance was not examined for the psychologists.  Unconscious social desirability had a significant impact on social distance for psychologists. The nature of this effect, and whether conscious social desirability was found to have an impact on social distance for psychologists was not reported. Conscious social desirability had a significant impact on social distance for social workers. The nature of this effect, and whether unconscious social desirability was found to have an impact on social distance for social workers was not reported.  Personal familiarity with mental illness was not found to have a significant impact on social distance for the mental health professionals. |
