# Supplementary material for: The stigmatization of mental illness by mental health professionals: Scoping review and bibliometric analysis
Source: PLoS One. 2023 Jan 20;18(1):e0280739. doi: 10.1371/journal.pone.0280739 (PMC9858369; doi:10.1371/journal.pone.0280739)
Supplement: S10 Appendix — (DOCX) [file pone.0280739.s010.docx]

| **Authors (year)** | **Populations**  **(countries)** | **Research methods** | **Analytical approaches** | **Disorders** | **Variables and measures** | **Findings** |
| --- | --- | --- | --- | --- | --- | --- |
| Qureshi et al. (2004) | GPs  (Saudi Arabia) | Longitudinal survey  An intervention was used | McNemar test | Mental illness in general (label)  Depression (label)  Schizophrenia (label)  Panic attacks (label) | Causal attributions (it was not clear what these were)  Social distance  Some people with mental illness are very difficult to communicate with and are less assertive  Views about mental hospitals (it was not clear what this measure was about)  Closure of mental hospitals  Chronicity and prognosis of mental disorder  Admission to mental hospital (it was not clear what this measure was about)  Productivity and people with mental illness  Creativity and people with mental illness  Fear of mental disorder  Clarity of mental disorder (it was not clear what this measure was about)  Reproduction and people with mental illness | For most of the measures, there was either a lack of clarity as to what was being measured, or not enough information was provided for a complete summary of the findings.  Prior to a course on psychiatry, roughly half of the participants expressed positive attitudes for one of the social distance items towards mental illness, and most participants disagreed that some people with mental illness are very difficult to communicate with and are less assertive (don’t know was an available option). None of the other social distance items or measures were able to be summarised in this manner.  After the course on psychiatry, participants were significantly more likely to express positive attitudes for the previously mentioned social distance item, and significantly more likely to disagree that some people with mental illness are very difficult to communicate with and are less assertive. For most of the remaining items, time point was not found to have a significant impact on the measures. The only exceptions to this were the causal attribution items for depression, schizophrenia, and panic attacks. For these items, time point had a significant impact. However, the change that occurred between pre and post-intervention was not made clear. |
| Ralley et al. (2009) | Mental health nurses  Nursing assistants |  |  |  |  | Nothing more was reported for this study as findings were not reported for mental health nurses separately. |
| Rao et al. (2009) | Unspecified nurses working in mental health and medical facilities  Unspecified doctors working in mental health and medical facilities  Healthcare assistants  Other unspecified health professionals |  |  |  |  | Nothing more was reported for this study as findings were not reported for mental health professionals separately. |
| Reavley et al. (2014) | GPs  Clinical psychologists  Psychiatrists  (Australia) | Experiment  Vignettes were used | Between-groups ANOVA | Depression (description)  Depression with suicidal thoughts (description)  Early schizophrenia (description)  Chronic schizophrenia (description)  Social phobia (description)  PTSD (description) | A measure of stigmatising attitudes  Weak not sick  The target could snap out of the problem  The problem the target is experiencing is a sign of personal weakness  The problem the target is experiencing is not a real medical illness  It is best to avoid people with the target’s problem  Dangerous/unpredictable  People with the target’s problem are dangerous  People with the target’s problem are unpredictable  It is best to avoid people with the target’s problem (this item was also included with the previous factor)  If I had that target’s problem, I wouldn’t tell anyone  I would not employ someone with the target’s problem  I would not vote for a politician with the target’s problem  Social distance  Sex  Age  <39  40-49  50-59  60+  Profession | Participants expressed less stigmatisation for weak not sick, dangerous/unpredictable, and social distance for all mental disorders. The only exception to this was participants expressed more social distance towards chronic schizophrenia.  The following are listed from the mental disorders considered the most weak not sick to the least.  Social phobia  Depression  PTSD  Both types of schizophrenia  The following are listed from the mental disorders considered the most dangerous/unpredictable to the least (this was the same for social distance, except depression was stigmatised more than PTSD).  Both types of schizophrenia  PTSD and depression  Social phobia  Although it was reported that there were statistically significant differences between the mental disorders, pairs that were significantly different were not noted by the authors.  Compared to depression, depression with suicidal thoughts was stigmatised slightly more for dangerous/unpredictable, more for social distance, and less for weak not sick. Also, compared to early schizophrenia, chronic schizophrenia was stigmatised slightly less for weak not sick, and more for dangerous/unpredictable and social distance. Again, whether these differences were statistically significant was not reported.  Compared to females, males expressed significantly more stigmatisation for both factors and social distance.  Age was not found to have an impact on weak not sick. For dangerous/unpredictable and social distance age had a significant impact. For these two variables, as age increased, stigmatisation increased. However, differences between the age groups were not examined with multiple companions.  For the two attitude factors, GPs expressed significantly more stigmatisation than psychiatrists and psychologists. For social distance, no significant difference was found between GPs and psychiatrists, and GPs expressed significantly more stigmatisation than psychologists. For dangerous/unpredictable and social distance, psychiatrists expressed significantly more stigmatisation than psychologists. For weak not sick, psychologists expressed significantly more stigmatisation than psychiatrists.  Some statistically significant interaction effects were found between sex, age, and profession. However, this was all that was reported. |
| Reid et al. (2001) | GPs  (England) | Cross-sectional survey with closed and open-ended questions  A vignette was used | Mantel-Haenszel test | MUS (description and label) | Patients with MUS are difficult to manage  Most MUS are related to not wanting to go back to work, and medical sick benefits usually help to keep symptoms away  Received postgraduate psychiatric training  Practice based psychological support (i.e., psychologist, psychiatric nurse, counsellor) | Most participants agreed that MUS are difficult to manage.  One participant added the comment, most MUS are related to not wanting to go back to work, and medical sick benefits usually help to keep symptoms away.  Having received postgraduate psychiatric training and having access to practice based psychological support was not found to have a significant impact on perceptions of difficulty. |
| Richmond & Foster (2009) | Social workers  Occupational therapists  Psychologists  A psychiatrist  Unspecified nurses from mental health and non-mental health facilities  Other unspecified doctors from mental health and non-mental health facilities  Support workers |  |  |  |  | Nothing more was reported for this study as findings were not reported for mental health professionals separately. |
| Rivera-Segarra et al. (2019) | GPs  Internal medicine physicians  Unspecified nurses  (Puerto Rico) | Semi-structured interviews | Thematic analysis | Mental illness in general (label) | People with mental illness do not love their family members  Perceived dangerousness  People with mental illness drink a lot  People with mental illness are not educated  Causal attributions  People with mental illness should be able to reach their psychiatrists  Prognosis | One GP believed that people with mental illness do not love their family members and will try to kill them. They stated that people with mental illness drink a lot and are not educated. Also, not being educated was believed to be part of what causes mental illness. However, this GP believed that people with mental illness should be able to reach their psychiatrists.  Another GP believed that mental illness is not curable. |
| Robinson (1973) | Psychiatric nurses  Student nurses  (New Zealand) | Cross-sectional survey | Independent samples t-test | Mental illness in general (label) | OMI scale  Authoritarianism  Benevolence  Mental hygiene ideology  Social restrictiveness  Interpersonal aetiology  Sex  Seniority  Ward sister/charge  Staff nurse | For authoritarianism, psychiatric nurses expressed more negative attitudes. However, for benevolence and mental hygiene ideology psychiatric nurses expressed more positive attitudes. The only exception to this was female staff nurses expressed less positive attitudes for benevolence. For social restrictiveness, ward sisters expressed more negative attitudes, female staff nurses expressed less negative attitudes, and male staff nurses expressed roughly neutral attitudes.  For interpersonal aetiology, ward sisters and charges expressed more agreement, whereas female staff nurses expressed less agreement, and male staff nurses expressed roughly neutral responses.  Male psychiatric nurses expressed either more or slightly more agreement with every OMI factor compared to female psychiatric nurses. Sex was not examined with inferential statistics for psychiatric nurses separately.  For male psychiatric nurses, seniority was not found to have a significant impact on any of the OMI factors.  For female psychiatric nurses, ward sisters expressed significantly more agreement with authoritarianism, benevolence, and interpersonal aetiology, compared to staff nurses. Seniority was not found to have a significant impact on mental hygiene ideology and social restrictiveness for female psychiatric nurses.  Other relevant findings were excluded from this table as they were not reported for psychiatric nurses separately. |
| Roche et al. (1991) | GPs  (Australia) | Focus groups | The analytical procedure used was not clear | Substance abusers (label)  Opioid dependence (label)  Alcohol abuse (label) | Perceived difficulty  Substance abusers are nasty  Perceived dangerousness  Substance abusers are a waste of time and resources  Substance abusers are time consuming  Substance abusers are not financially rewarding enough to counsel  Prognosis  Whether substance abusers are motivated to change  Substance abusers need to be listened to and encouraged  Substance abusers need to be given support  Causal attributions  People dependent on opioids present to surgery at inconvenient times  People dependent on opioids look scruffy  People dependent on opioids look unhealthy  People dependent on opioids have long-winded stories  Avoidance  Vehement dislike  Discomfort with avoidance  People dependent on opioids never really want help | Participants endorsed the stereotypes that substance abusers are difficult, nasty, dangerous, and a waste of time and resources. Also, participants expressed that substance abusers are time consuming, and one participant expressed that it is not financially rewarding to counsel them.  One participant expressed that substance abusers cannot be helped. However, another participant stated that GPs have a role in substance abuse to the extent that substance abusers are motivated to change.  One participant stated that you need to listen to and encourage substance abusers, and another stated that GPs should provide support to substance abusers.  One participant expressed that there is nothing that can be done when substance abuse is due to poverty.  Participants stated that people dependent on opioids tend to present to surgery at inconvenient times, look scruffy and unhealthy, and have long-winded stories. Most participants wanted nothing to do with people dependent on opioids, would often send them away, and vehemently disliked them in general. However, some participants were prepared to provide treatment. Also, some participants felt uncomfortable sending people dependent on opioids away. Despite this, the participants also justified this based on the appearance and general behaviour of people dependent on opioids. People dependent on opioids were seen by one participant as never really wanting help.  Many participants believed that alcohol abuse is a lifestyle problem, and this is why you can’t help people that abuse alcohol. Participants were also reluctant to counsel people that abuse alcohol because of this hopelessness. |
| Ronzani et al. (2009) | Social workers  Psychologists  Administrative technicians  Community health workers  Nursing assistants  Unspecified nurses  Unspecified physicians  Dental care professionals  Unspecified students  Other unspecified professionals |  |  |  |  | Nothing more was reported for this study as findings were not reported for mental health professionals separately. |
| Rosendal et al. (2005) | GPs  (Denmark) | Experiment  An intervention was used | Mann-Whitney *U-*test  Independent samples t-test | Somatoform disorder (label) | People with somatoform disorder take up too much time  Anxiety  Anger  Somatising patients are more likely to have experienced deprivation in early life than other people  The majority of somatising conditions in general practice originates from patients’ conditions of life  It is possible to distinguish two main groups of somatisation: stress induced and more genuine somatisation disorders  Somatising is a way that people with poor stamina deal with life difficulties  An underlying biochemical abnormality is at the basis of severe cases of somatisation  Most somatising conditions in general practice improve without treatment  Somatisation reflects a characteristic response in patients which is not amenable to change  I feel comfortable in dealing with somatising patients | No significant differences were found between the control group and the intervention group prior to being assigned to these groups. Participants believed that people with somatoform disorder take up too much time, but experience anxiety and anger in the presence of people with somatoform disorder less. Participants also agreed more with all of the measures about causal attributions. The only exception to this was participants disagreed more that an underlying biochemical abnormality is at the basis of severe cases of somatisation. For the remaining measures, participants expressed more disagreement.  One year following the intervention (an educational programme on treating somatoform disorder) participants in the intervention group expressed significantly less anxiety and significantly more comfort in dealing with somatising patients, compared to before the intervention. Control group participants became more anxious and less comfortable. For the remaining measures of stigmatisation, no significant difference was found between pre and post-intervention scores. |
| Roskin et al. (1988) | Psychiatrists  Psychologists  Social workers  Psychiatric nurses  Psychiatric residents  (USA) | Cross-sectional survey | Between-groups ANOVA | Mental illness in general (label) | Causal attributions  Profession | Participants (excluding psychiatrists and psychiatric residents) agreed more with a biological aetiology of mental illness, but agreed even more with a psychodynamic aetiology of mental illness. The only exception to this was psychologists responded neutrally to a biological aetiology of mental illness. An overview of scores for moral weakness as a cause for mental illness was not reported.  Profession had a significant impact on biological aetiology. All professions were significantly different from each other. The following are the professions listed from least endorsement of biological aetiology to most endorsement.  Psychologists  Social workers  Nurses  Profession had a significant impact on psychodynamic aetiology. Differences between psychologist, social workers, and nurses were not found to be statistically significant. Profession was not found to have a significant impact on moral weakness as a cause of mental illness.  Other relevant findings were excluded from this table as they were not reported for mental health professionals separately. |
| Ross et al. (1999) | GPs  (Scotland) | Cross-sectional survey  Vignettes were used | Bivariate logistic regression analysis | Depression (label) | DAQ  Inevitable course of depression  Depression reflects a characteristic response in patients that is not amenable to change  Becoming depressed is a natural part of being old  There is little to be offered to those depressed patients who do not respond to what GPs do  Psychotherapy tends to be unsuccessful with depressed patients  If depressed patients need antidepressants, they are better off with a psychiatrist than a GP  Professional confidence  I feel comfortable in dealing with depressed patients’ needs  Working with depressed patients is heavy going  It is rewarding to spend time looking after depressed patients  Antidepressants usually produce a satisfactory result in the treatment of depressed patients in general practice  If psychotherapy were freely available, it would be more beneficial than antidepressants for most depressed patients  Social model of depression  The majority of depression seen in general practice originates from patients’ recent misfortunes  Most depressive disorders seen in general practice improve without medication  Depressed patients are more likely to have experienced deprivation in early life than other people  The practice nurse could be a useful person to support depressed patients  During the past five years, I have seen an increase in the number of patients presenting with depressive symptoms  An underlying biochemical abnormality is at the basis of severe cases of depression  It is difficult to differentiate whether patients are presenting with unhappiness or with clinical depressive disorder that needs treatment  It is possible to distinguish two main groups of depression: one psychological in origin and the other caused by biochemical mechanisms  Becoming depressed is a way that people with poor stamina deal with life difficulties  Psychotherapy for depressed patients should be left to a specialist  Psychiatric drug prescribing behaviour | Most of the participants disagreed that depression reflects a characteristic response in patients that is not amenable to change. However, less than half of the participants agreed that most depressive disorders seen in general practice improve without medication. Most participants disagreed that becoming depressed is a natural part of being old, and becoming depressed is a way that people with poor stamina deal with life difficulties. In contrast, most participants agreed that an underlying biochemical abnormality is at the basis of severe cases of depression. Less than half of the participants agreed that depressed patients are more likely to have experienced deprivation in early life than other people, and roughly half of the participants disagreed that the majority of depression seen in general practice originates from patients’ recent misfortunes, and it is possible to distinguish two main groups of depression: one psychological in origin and the other caused by biochemical mechanisms (neutral responses were available).  An overview of the findings for the other DAQ items was not included in this table as they were not relevant to stigmatisation.  Participants that scored high on overall social model of depression were significantly less likely to prescribe psychiatric drugs for a vignette that portrayed a crying patient with suicidal thoughts. Overall social model of depression was not found to be a significant predictor of psychiatric drug prescribing behaviour for a vignette portraying a patient that feels tired all the time. |
| Roussy et al. (2015) | Counsellors  Unspecified allied health staff  Unspecified oral health staff  Health promotion staff  Receptionists |  |  |  |  | Nothing more was reported for this study as findings were not reported for mental health professionals separately. |
| Russell et al. (2021) | Physicians, nurse practitioners, residents, and physician assistants with one of the following specialities  Addition medicine  Family medicine  Psychiatry  Emergency medicine  Geriatrics  Internal medicine  Paediatrics  Obstetrics and gynecology  Palliative care  Surgery  Other unspecified  Administrative staff  Unspecified students  Other unspecified participants |  |  |  |  | Nothing more was reported for this study as findings were not reported for mental health professionals separately. |
