# Supplementary material for: The stigmatization of mental illness by mental health professionals: Scoping review and bibliometric analysis
Source: PLoS One. 2023 Jan 20;18(1):e0280739. doi: 10.1371/journal.pone.0280739 (PMC9858369; doi:10.1371/journal.pone.0280739)
Supplement: S11 Appendix — (DOCX) [file pone.0280739.s011.docx]

| **Authors (year)** | **Populations**  **(countries)** | **Research methods** | **Analytical approaches** | **Disorders** | **Variables and measures** | **Findings** |
| --- | --- | --- | --- | --- | --- | --- |
| Sabin et al. (2015) | Psychiatric nurses  Clinician therapists  Psychiatrists  Psychologists  Case managers  Program managers/supervisors  Case aides  Employment specialists  Peer support counsellors  Residential support counsellors  Co-occurring disorder specialists  Other unspecified health professionals |  |  |  |  | Nothing more was reported for this study as findings were not reported for mental health professionals separately. |
| Sagduyu et al. (2008) | GPs  (Turkey) | Structured interviews  Vignettes were used | Multiple regression analysis | Depression (description and label)  Addiction (label) | Causal attributions  Prognosis  Social distance  Perceived aggression  Segregation  How depression was presented  Description  Label  Having a relative with a psychiatric disorder  Age  Marital status  Married  Widow/widower  Separated/divorced  Never married | Most participants agreed that depression is a disease. Slightly more participants agreed with this when depression was presented with a description compared to a label. Roughly half of the participants agreed that depression is a state of mental weakness. However, most participants disagreed with this when depression was presented with a description. Most participants agreed that depression occurs because of social problems. Slightly more participants agreed with this when depression was presented with a label, compared to a description. Also, most participants agreed that changes in the environment contribute greatly to depression recovery. Most participants disagreed that depression is congenital and contagious (no opinion was an available option). Causal attribution differences between the description of depression and the label were not examined with inferential statistics.  Most participants disagreed that drugs used in the treatment of depression create addiction (no opinion was an available option).  For the remaining measures of stigmatisation, most participants expressed a lack of stigmatisation towards depression.  Having a relative with a psychiatric disorder, older age, and being married (the reference group was not specified) were significant predictors of less social distance towards depression. It was also suggested that beliefs about whether depression is a disease, and other aetiological beliefs were included as predictors of social distance towards depression. However, it was not clear whether these variables were found to be significant predictors of social distance, and the levels for the other aetiological beliefs were not specified. |
| Sakellari et al. (2020) | Unspecified nurses from a psychiatric hospital  Midwives  Health visitors  (Greece) | Cross-sectional survey | - | Schizophrenia (description and a possible label) | AQ-27 (items were not specified)  Familiarity with mental illness  Perceived personal responsibility  Pity  Anger  Fear  Helping  Coercion-segregation  Attitudes towards breastfeeding among women with schizophrenia (items were not specified) | Scores on both measures were unable to be interpreted with the information provided.  Other relevant findings were excluded from this table as they were not reported for mental health professionals separately. |
| Salime et al. (2019) | Psychiatrists  Psychologists  Mental health nurses  Other unspecified physicians  Other unspecified nurses  Nurse assistants  Medico-psychological assistants  (France) | Unstructured interviews | Categorical analysis  Similarity analysis | Mental illness in general (label) | People with mental illness are in cognitive, physical, and relational decline  Causal attributions  Perceived dangerousness  Perceived likelihood of violence  Fear  Perceived difficulty  Prognosis  People with mental illness are unable to make judgments  People with mental illness are passive  People with mental illness are ugly  People with mental illness are dependent  People with mental illness cause burnout  Profession | A proportion of mental health professionals perceived elderly people with mental illness as being in cognitive, physical, and relational decline (this was likely due to many of the participants viewing mental illness in elderly people as dementia).  Another proportion of mental health professionals perceived mental illness as a disease. One subcomponent of this was personality structure. However, this was all that was reported. Also, other subcomponents of the disease theme were not made clear.  Psychologists and mental health nurses associated mental illness with danger, violence, and fear. These two groups also viewed mental illness in the elderly as difficult to care for and untreatable. Also, psychologists perceived people with mental illness as unable to make judgments and passive. Both psychologists and psychiatrists viewed mental illness as ugly, and all three groups of mental health professionals associated mental illness with dependence and burnout. Psychologists associated mental illness with the most amount of negative attributes, and psychiatrists associated mental illness with the least amount of negative attributes.  Other themes were identified that may have been relevant to stigmatisation. However, whether these themes were relevant to stigmatisation was not clear. Thus, these themes were not included in this table.  Other relevant findings were excluded from this table as they were not reported for mental health professionals separately. |
| Salt et al. (2005) | GPs  (England) | Cross-sectional survey | - | ADHD (label) | Causal attributions  People with ADHD under achieve in an educational setting  People with ADHD are antisocial  Prognosis | Participants attributed ADHD to a range of causes with varying proportions. Genetic inheritance, chemical imbalance, and quality of parenting were among the most likely causes, and social class, peer group influences, and ethnicity were among the least likely causes.  Most participants believed that people with ADHD under achieve in an educational setting and are antisocial.  Most participants disagreed that ADHD only exists in children. |
| Sandhu et al. (2019) | Psychiatrists  Psychiatric residents  Medical students  Business, engineering, health science, science, and social science students |  |  |  |  | Nothing more was reported for this study as findings were not reported for psychiatrists separately. |
| Scheerder et al. (2011) | Psychologists  Psychiatrists  Mental health nurses  Counsellors  Social workers  A range of other nurses  Nurse assistants  A range of other doctors  Police officers  Priests  Pastoral workers  Youth advice service workers  Teachers  Pharmacists  Volunteers |  |  |  |  | Nothing more was reported for this study as findings were not reported for mental health professionals separately. |
| Schwartz (2003) | Social workers  (Israel) | Cross-sectional survey | - | Mental illness in general (label) | CLAS-MI (items were not specified by the authors and only factors relevant to stigmatisation were included in this table)  Empowerment (relates to the rights of persons with mental illness to make their opinions felt in decisions and policies affecting their lives)  Exclusion (the desire to segregate persons with mental illness from community life)  Sheltering (the extent to which the respondent believes that persons with mental illness need to have others supervise them in their daily lives or protect them from the dangers of community life) | Participants expressed more positive attitudes on the empowerment and exclusion factors, and neutral responses on the sheltering factor. |
| Servais & Saunders (2007) | Clinical psychologists  (USA) | Cross-sectional survey | Repeated-measures ANOVA  Multiple regression analysis | Borderline features (label)  Schizophrenia (label)  Moderate depression (label) | Effective-ineffective  Understandable-incomprehensible  Safe-dangerous  Worthy-unworthy  Desirable to be with-undesirable to be with  Similar-dissimilar | Overall, stigmatisation was dependent on the type of mental disorder and measure of stigmatisation. In some cases, participants expressed more stigmatisation, and in other cases participants expressed less stigmatisation. However, moderate depression elicited less negative attitudes for all measures of stigmatisation.  For ineffectiveness and incomprehensible, the following mental disorders are listed from most stigmatisation to least.  Schizophrenia  Borderline features  Moderate depression  All of these mental disorders were significantly different from each other.  For dangerous, unworthy, and undesirable to be with, the following mental disorders are listed from most stigmatisation to least.  Borderline features  Schizophrenia  Moderate depression  All of these mental disorders were significantly different from each other, except moderate depression and schizophrenia were not found to be significantly different for unworthy.  When only examining schizophrenia, perceptions of ineffectiveness and dangerousness were significant predictors of participants perceiving a person with schizophrenia as dissimilar to them. Unworthy and incomprehensible were not found to be significant predictors of perceived dissimilarity with schizophrenia. Whether these predictors of similarity regarded all of the mental disorders or just schizophrenia was not made clear.  When only examining borderline features, perceptions of ineffectiveness and dangerousness were significant predictors of participants not seeing a person with borderline features as desirable to be with. Unworthy and incomprehensible were not found to be significant predictors of a person with borderline features being seen as undesirable. Whether these predictors of desirability regarded all of the mental disorders or just borderline features was not made clear. |
| Sevigny et al. (1999) | Unspecified doctors working in a psychiatric hospital  Unspecified nurses working in a psychiatric hospital  (China) | Cross-sectional survey | The analyses were not clear | Mental illness in general (label) | Chinese version of the CAMI (only items relevant to stigmatisation were included in this table)  Benevolence, kindness, and sympathy  Locating mental health institutions in a residential area downgrades the neighbourhood  The state should provide more funding on the care and treatment of the mentally ill  As far as possible community based mental health services should be provided  Increased spending on mental health services is a waste  No one has the right to exclude the mentally ill from their neighbourhood  The mentally ill should not be treated as if they are outcasts of society  We have a responsibility to provide the best medical treatment for the mentally ill  Exclusion based on characteristics attributed to the mentally ill  It is very easy to tell the mentally ill from normal people  Anyone with a history of mental problems should be excluded from taking public office  Mental health facilities should be kept out of residential neighbourhoods  The mentally ill should not be given any social responsibilities  Social exclusion based on the respondent’s own personal reactions  Having mental patients living within residential neighbourhoods might be the best therapy but the risks to residents are too great  Mental patients need the same kind of control and discipline as a young child  I would not want to have a neighbour who has been mentally ill  It is frightening whenever to think of people with mental problems living nearby  It is best not to have any contact with a person who has mental problems  As soon as a person shows signs of mental disturbance, he should be hospitalized  The mentally ill should not be isolated from the rest of the community  The most effective therapy for many mental patients is to let them go back to a normal community  Mental illness is an illness like any other  The mentally ill are a burden on society  The mentally ill are far less of a danger than most people imagine  The situation that the mentally ill have for too long been the subject of ridicule should be put to an end  A woman would be very unwise to marry a man who has suffered from mental illness, even though he seems to have regained normality  There should not be any over-emphasis that the mentally ill endanger the public  Society should adopt a far more tolerant attitude toward the mentally ill  Residents should accept the location of mental health institutions in their neighbourhood to serve the needs of the residents  Mental patients should be encouraged to assume the responsibilities of normal life  Residents have good reason to resist the location of mental health institutions in residential areas  The best way to handle the mentally ill is to keep them behind locked doors  Our mental hospitals seem more like prisons than like places where the mentally ill can be treated  Locating mental health facilities in the community does not endanger local residents  Mental hospitals are an out-dated means of treating the mentally ill  The mentally ill don’t deserve our sympathy  One of the main causes of mental illness is a lack of self-discipline and will power  Residents have nothing to fear from people coming into their neighbourhood to obtain mental health services  Virtually anyone can become mentally ill  Most women who were once patients in a mental hospital can be trusted to take care of babies  Profession | CAMI factor scores were unable to be fully interpreted with the information provided. Overviews were reported for only 11 CAMI items individually. One of these items was mental illness is an illness like any other. For this item, most doctors agreed, whereas less than half of the nurses agreed. For the other items, most doctors expressed positive attitudes. The only exception to this was for one of the items only half of the doctors expressed positive attitudes. For the nurses, most nurses expressed negative attitudes for most of the items. However, for two items, roughly half of the nurses expressed positive attitudes, and for one item most nurses expressed positive attitudes.  Doctors were significantly more likely to agree that mental illness is an illness like any other, compared to nurses. For the remaining items that were summarised individually, doctors were significantly less likely to stigmatise mental illness compared to nurses. For the items that were not summarised individually, profession was not found to have a significant impact (i.e., most of the items). |
| Shanks et al. (2011) | Social workers  Counsellors  Psychologists  Psychiatrists  Unspecified nurses  Physician assistants  Probation officers |  |  |  |  | Nothing more was reported for this study as findings were not reported for mental health professionals separately. |
| Shao et al. (1997) | Psychiatrists  Family physicians  Obstetricians and gynaecologists  Internists  Psychiatric residents  Family physician residents  Resident obstetricians and gynaecologists  Resident internists |  |  |  |  | Nothing more was reported for this study as findings were not reported for mental health professionals separately. |
| Shinan-Altman et al. (2014) | Social workers  Unspecified nurses  (Israel) | Cross-sectional survey | - | Alzheimer’s disease (label) | Causal attributions  Prognosis  Perceived personal control  Negative emotions (only the following examples were provided)  Depression  Fear  Anger  People with Alzheimer’s disease are apathetic  People with Alzheimer’s disease are grumpy  People with Alzheimer’s disease are restless | Social workers agreed the most with attributing cause to risk factors such as heredity and smoking, followed by personality, stress, and worry, followed by an accident or chance, followed by germs and viruses.  Social workers perceived Alzheimer’s disease as more chronic, and reflecting slightly more personal control.  Social workers expressed less overall negative emotions.  Most social workers believed that people with Alzheimer’s disease are apathetic, grumpy, and restless.  Other relevant findings were excluded from this table as they were not reported for social workers separately. |
| Shinan-Altman et al. (2016) | Social workers  Unspecified nurses |  |  |  |  | Nothing more was reported for this study as findings were not reported for social workers separately. |
| Siegfried et al. (1999) | Psychiatrists  Psychologists  Social workers  Occupational therapists  Unspecified nurses  Other unspecified health professionals |  |  |  |  | Nothing more was reported for this study as findings were not reported for mental health professionals separately. |
| Smith & Cashwell (2010) | Social workers  Psychologists  Counsellors  Business professionals  Social work students  Psychology students  Counselling students  Business administration students  (USA) | Cross-sectional survey | MANOVA | Mental illness in general (label) | CAMI  Authoritarianism  Benevolence  Social Restrictiveness  Community Mental Health Ideology  Currently receiving clinical supervision | Mental health professionals expressed less stigmatisation across all CAMI factors.  Mental health professionals that were receiving clinical supervision had significantly more positive attitudes for benevolence compared to mental health professionals that were not receiving clinical supervision. Currently receiving supervision was not found to have a significant impact on the other CAMI factors for the mental health professionals.  Other relevant findings were excluded from this table as they were not reported for mental health professionals separately. |
| Smith & Cashwell (2011) | Social workers  Psychologists  Counsellors  Business professionals  Social work students  Psychology students  Counselling students  Business students  (USA) | Cross-sectional survey | Factorial ANOVA | Mental illness in general (label) | Social distance  Profession | Mental health professionals expressed less social distance.  Psychologists and counsellors expressed the exact same amount of social distance. Psychologists and counsellors expressed significantly less social distance than social workers.  Other relevant findings were excluded from this table as they were not reported for mental health professionals separately. |
| Smith et al. (2017) | Mental health nurses  Psychiatrists  Psychologists  Primary care physicians  Primary care nurses  (USA) | Experiment  Vignettes was used | Multiple regression analysis | Schizophrenia (description and label)  A range of physical conditions (description and labels) | Social distance  Semantic differentials  Valuable-worthless  Clean-dirty  Sincere-insincere  Safe-dangerous  Warm-cold  Wise-foolish  Strong-weak  Predictable-unpredictable  Tense-relaxed  AQ-9  I would think that it was the patient’s own fault that he is in the present condition  I would think that the patient is dangerous  I would think that the patient should be forced into treatment with his doctor even if he does not want treatment  I would be angry with the patient  I would be scared of the patient  I would try to stay away from the patient  Profession  Age (control variable)  <30  31-40  41-50  51-60  >60  Sex (control variable)  Race (control variable)  White  Non-White  Years of professional experience (control variable) | Mental health nurses, psychiatrists, psychologists, and physicians expressed less overall negative attitudes on all measures of stigmatisation towards both vignettes (stigmatisation scores were not provided for schizophrenia separately).  Accounting for the control variables, the interaction between profession and vignette type was a significant predictor of social distance. For the schizophrenia vignette, physicians and psychiatrists expressed significantly more social distance than psychologists and mental health nurses. Other profession comparisons were not reported for schizophrenia alone. However, taking the two vignettes together, physicians expressed more social distance than psychiatrists, and no significant difference was found between psychologists and mental health nurses (accounting for the control variables). The difference between physicians and psychiatrists was not examined with inferential statistics.  Accounting for the control variables, the interaction between profession and vignette type was a significant predictor of overall semantic differential scores. Physicians expressed significantly more negative attitudes than psychologists and mental health nurses. Again, other profession comparisons were not reported for schizophrenia alone. Taking the two vignettes together, physicians expressed more negative attitudes than psychiatrists, who in turn expressed more negative attitudes than mental health nurses, and psychiatrists and mental health nurses were not found to be significantly different to psychologists (accounting for the control variables). The differences between physicians and psychiatrists, and psychiatrists and mental health nurses, were not examined with inferential statistics.  Accounting for the control variables, the interaction between profession and vignette type was not found to be a significant predictor of overall AQ-9 scores. Thus, professions were not compared for schizophrenia separately. Taking the two vignettes together, physicians expressed the most negative attitudes, followed by psychiatrists, followed by mental health nurses, followed by psychologists. These differences were not examined with inferential statistics.  Other relevant findings were excluded from this table as they were not reported for mental health professionals separately. |
| Spagnolo et al. (2018) | Primary care physicians  (Tunisia) | Cross-sectional survey | - | Mental illness in general (label) | I would use the terms “crazy,” “nutter,” “mad,” etc. to describe to colleagues people with a mental illness who I have seen in my work  People with severe mental illness can never recover enough to have a good quality of life  I feel comfortable talking to a person with mental illness as I do talking to a person with physical illness  People with mental illness are dangerous more often than not  The public does not need to be protected from people with mental illness | For the first two measures, most participants expressed positive attitudes. However, for the remaining measures most participants expressed negative attitudes. |
| Stang et al. (2006) | Family physicians  Internists  Resident physicians |  |  |  |  | Nothing more was reported for this study as findings were not reported for family physicians separately. |
| Stefanovics et al. (2016) | Social workers  Psychiatrists  Nurses from a psychiatric hospital  Primary care physicians  Other unspecified nurses  Medical students  (USA, Brazil, Ghana, Nigeria, China) | Cross-sectional survey | - | Mental illness in general (label) | A measure of attitudes towards mental illness (items were not specified)  Socialising (positive attitudes towards socialising with people with mental illness)  Normalising (a belief that socially people with mental illness should adopt normalised roles)  Non-supernatural factor (not endorsing witchcraft or curses as causes of mental  Illness)  Biopsychosocial model (a belief in the biopsychosocial causation of mental illness) | For a subset of the psychiatrists and all of the nurses from a psychiatric hospital (i.e., the sample from China), most participants expressed overall positive attitudes for the first two factors, and most participants agreed with the non-supernatural factor overall, and biopsychosocial causation overall.  Other relevant findings were excluded from this table as they were not reported for mental health professionals separately. |
| Stefanovics et al. (2016) | Psychologists  Social workers  Psychiatrists  Nurses from a psychiatric hospital  Primary care physicians  Other unspecified nurses  Medical students  (USA, Brazil, Ghana, Nigeria, China) | Cross-sectional survey | Correlation analysis | Mental illness in general (label) | The same measure used in the above study (items were not specified) | Factor scores were not reported.  For a subset of the psychiatrists and all of the nurses from a psychiatric hospital (i.e., the sample from China) there was a significant positive correlation between overall biopsychosocial model and overall socialising, and between overall biopsychosocial model and overall normalising. Significant correlations were not found between overall non-supernatural factor, and overall socialising and normalising.  Other relevant findings were excluded from this table as they were not reported for mental health professionals separately. |
| Steinberg & Wetterneck (2017) | Professionals and students from the following fields  Clinical psychology  Behaviour analysis  Organisational psychology  Counselling psychology  School psychology  General psychology  Marriage and family therapy  Social work  Other |  |  |  |  | Nothing more was reported for this study as findings were not reported for mental health professionals separately. |
| Stephens et al. (2021) | Psychiatrists  Psychologists  Social workers  Counsellors or psychotherapists  Occupational therapists  Behavioural therapists  Unspecified physicians  Other unspecified clinicians  Unspecified healthcare students |  |  |  |  | Nothing more was reported for this study as findings were not reported for mental health professionals separately. |
| Stromwall et al. (2011) | Unspecified behavioural health clinicians  Peer employees  (USA) | Cross-sectional survey | - | Mental illness in general (label) | Perceived stigmatisation in general | The level of stigmatisation perceived by the behavioural health clinicians was unable to be interpreted with the information provided.  Other relevant findings were excluded from this table as they were not reported for the behavioural health clinicians separately. |
| Stuber et al. (2014) | Psychiatric nurses  Unspecified therapists and psychologists  Psychiatrists  Program managers and directors  Case managers  Other unspecified health professionals  (USA) |  |  |  |  | Nothing more was reported for this study as findings were not reported for mental health professionals separately. |
| Stull et al. (2013) | Social work professionals  Psychology professionals  Psychiatry professionals  Unspecified nursing professionals  Education professionals  Other unspecified professionals |  |  |  |  | Nothing more was reported for this study as findings were not reported for mental health professionals separately. |
| Sullivan et al. (2015) | Mental health physicians  Primary care physicians  Mental health nurses  Primary care nurses |  |  |  |  | Nothing more was reported for this study as findings were not reported for mental health professionals separately. |
| Sun et al. (2014) | Psychiatrists  Psychiatric nurses  (China) | Cross-sectional survey | ANCOVA | Mental illness in general (label) | A measure of attitudes that combined the FABI and the CAMI  Community based treatment, social integration, and a biopsychosocial model of causation  Personal preferences for direct personal relationships with people with mental illness    Fear-free and positive view of specific interactions with people with mental illness  In interacting with someone with mental illness, you were not upset or disturbed about working on the same job  Physical abuse cannot cause mental illness  People with mental illness are not a public nuisance  People with mental illness are not dangerous because of violent behavior  In interacting with someone with mental illness, I would not be unwilling to share a room  You would not avoid conversations with neighbors who had suffered from mental illness  Disbelief in superstitious explanations of mental illness  Profession  Age (control variable)  Sex (control variable)  Marital status (control variable)  Single  Married  Years of education in total (control variable)  Birthplace (control variable)  Urban  Semi-urban  Rural  Residence (control variable)  Urban  Semi-urban  Rural | For most of the factors, participants expressed either more or slightly more positive attitudes. The only exception to this was nurses expressed mixed attitudes for fear-free and positive view of specific interactions with people with mental illness.  After accounting for the control variables, profession was not found to have a significant impact on stigmatisation. |
| Tay et al. (2004) | Unspecified nurses working in a psychiatric hospital  Midwives working in a psychiatric hospital  Assistant nurses  (Singapore) | Cross-sectional survey | Independent samples t-test  Between-groups ANOVA | Mental illness in general (label) | AMI questionnaire  One can always tell a mentally ill person by his physical appearance  In order to work with the mentally ill, there is no need for professional knowledge  The mentally ill, with a number of exceptions, cannot tell the difference between good and bad  Mental illness is genetic  The mentally ill should be prevented from having children  Mental illness cannot be cured  One should avoid all contact with the mentally ill  Psychiatric hospitals should not be located in residential areas  Those who work in hospitals for the mentally ill do so because they have no other choice  The mentally ill should not get married  Life has no value for the mentally ill  It is not necessary to consider the opinion of a person who has been released from a mental hospital  The mentally ill should live only among themselves  There are people who were never in a mental hospital and are more disturbed than those who are in a mental hospital  Once crazy, always crazy  It is not necessarily true that a person who was once in a hospital for the mentally ill will continually have to return there  Usually, there is no way of telling when seeing a person walking in the street if he was ever in a hospital for the mentally ill  Very few, if any, mentally ill people are capable of true friendships  Mentally ill people should be prevented from walking freely in public places  One should hide his/her mental illness from his/her family  Mentally ill people who do not get well have no one to blame but themselves  Mentally ill people who are not hospitalised should be prevented from walking freely in public places at night  The mentally ill should not be allowed to make decisions, even those concerning routine events  Every mentally ill person should be in an institution where he/she will be under supervision and control  Seniority  Nursing officer  Senior staff nurse/staff nurse  Type of ward  Short-stay  Long-stay  Years of professional experience  1 to 10 years  11 to 20 years  21 to 30 years  31 years and above | Unspecified nurses working in a psychiatric hospital expressed more positive attitudes overall.  For the unspecified nurses working in a psychiatric hospital, nursing officers expressed more overall positive attitudes than senior staff nurses/staff nurses. This difference was not examined with inferential statistics separately for the unspecified nurses working in a psychiatric hospital.  For the unspecified nurses working in a psychiatric hospital type of ward was not found to have a significant impact on overall attitudes.  For the unspecified nurses working in a psychiatric hospital, years of professional experience was not found to have a significant impact on overall attitudes for nursing officers. However, years of professional experience was found to have a significant impact on overall attitudes for senior staff nurses/staff nurses. As years of experience increased overall attitudes became more positive, before they became more negative again for nurses with 31 and more years of experience. These differences were not examined with multiple comparisons.  Other relevant findings were excluded from this table as they were not reported for the mental health professionals separately. |
| Thomas-MacLean & Stoppard (2004) | Primary care physicians  (Canada) | Semi-structured interviews | Foucauldian discourse analysis | Depression (label) | Causal attributions  It is important to establish rapport with patients with depression | Most of the participants attributed depression to a biochemical imbalance and biological factors in general. Participants also seemed to downplay social factors in the aetiology of depression. Despite this, participants also expressed that people with depression need to accept at least some responsibility for their illness. In fact, one participant explained depression by referencing human laziness. Although social factors were downplayed, some participants also attributed depression to life stressors, lifestyle factors, and social inequalities.  Participants believed it was important to establish rapport with patients with depression. |
| Thornicroft et al. (1987) | Psychiatric nurses  (England and China) | Cross-sectional survey | Independent samples t-test | Schizophrenia (label) | Causal attributions  Perceived incomprehensible of speech, criminal behaviour, and unusual mood changes  Prognosis  People with schizophrenia should neither marry nor have children  Country | Descriptive statistics were not reported for causal attributions and stigmatisation.  Participants from China agreed significantly more than participants from England that the cause of schizophrenia is related to biochemical, infective, and genetic factors, and agreed significantly less that the cause of schizophrenia is related to past life events.  Participants from China agreed significantly more than participants from England that people with schizophrenia can be characterised by incomprehensible speech, criminal behaviour, and unusual mood changes. Further, participants from China agreed significantly more than participants from England that people with schizophrenia will not go on to acquire full employment, can expect their condition to become steadily worse, and should neither marry nor have children.  No other significant differences were identified for any other variables (it was not clear what these variables were).  Inferential statistics were not reported in this study. |
| Tracey (1988) | Psychotherapists  Psychology students  Counselling students |  |  |  |  | Nothing more was reported for this study as findings were not reported for psychotherapists separately. |
| Treloar (2009) | Mental health nurses  Practitioners from the following fields  Psychology  Social work  Occupational therapy  Psychiatrists  Other unspecified nurses  Other unspecified medical practitioners |  |  |  |  | Nothing more was reported for this study as findings were not reported for mental health professionals separately. |
| Tulachan et al. (2018) | Psychiatrists  (Nepal) | Cross-sectional survey | Chi-square test of independence | Cluster B personality disorders (label)  Personality disorder (label) | What would be your honest feeling toward people with cluster B personality disorders?  Feel angry  Feel manipulated  Feel affectionate  Feel excited  Feel helpless  Perceived difficulty  Avoidance  Sex  Years of professional experience  Less than 10 years  10 to 20 years  More than 20 years  Primary employment setting  Academic  Private practice | Participants felt helpless and manipulated more than other feelings. Fewer Participants felt affectionate, and even fewer felt angry and excited.  Most participants perceived personality disorder as very difficult.  Most participants would not avoid caring for personality disorder if they had the choice.  Sex was found to be significantly related to overall feelings towards cluster B personality disorders. Most males expressed overall positive feelings, whereas most females expressed overall negative feelings. Sex was not found to be significantly related to the other measures of stigmatisation.  Years of professional experience and primary employment setting were not found to be significantly related to any of the measures of stigmatisation. |
| Tungchama et al. (2019) | Social workers  Clinical psychologists  Unspecified medical doctors  Unspecified nurses  Pharmacists  Administrators  Record officers  Accountants  Auditors |  |  |  |  | Nothing more was reported for this study as findings were not reported for mental health professionals separately. |
