# Supplementary material for: The stigmatization of mental illness by mental health professionals: Scoping review and bibliometric analysis
Source: PLoS One. 2023 Jan 20;18(1):e0280739. doi: 10.1371/journal.pone.0280739 (PMC9858369; doi:10.1371/journal.pone.0280739)
Supplement: S12 Appendix — (DOCX) [file pone.0280739.s012.docx]

| **Authors (year)** | **Populations**  **(countries)** | **Research methods** | **Analytical approaches** | **Disorders** | **Variables and measures** | **Findings** |
| --- | --- | --- | --- | --- | --- | --- |
| Ucok et al. (2006) | GPs  (Turkey) | Longitudinal survey  An intervention was used | Chi-square test of independence  McNemar test | Schizophrenia (label) | What is the course of schizophrenia?  Patients with schizophrenia can work  Would oppose if one of his/her relatives would like to marry someone who has schizophrenia  Schizophrenia patients could be recognized by his/her appearance  Schizophrenia patients are dangerous  Would not like to have a neighbour with schizophrenia  Schizophrenia patients are untrustworthy  Schizophrenia patients could harm children  Schizophrenia patients should be kept in hospitals  I don’t worry about examining a person who is diagnosed with schizophrenia  Schizophrenia could be treated  Patients with schizophrenia could not comprehend nor apply suggested treatment  Schizophrenia has the chance of recovery  Sex  Personal familiarity with schizophrenia | Percentages for the question about the course of schizophrenia were not reported clearly.  Prior to interactive training sessions on schizophrenia, and for the majority of the remaining measures, most participants expressed positive attitudes towards schizophrenia. However, most participants would oppose if one of his/her relatives would like to marry someone who has schizophrenia, and would not like to have a neighbour with schizophrenia. Also, roughly half of the participants agreed that schizophrenia patients are untrustworthy.  Prior to training, there was a significant relationship between sex and two measures of stigmatisation. These measures regarded the course of schizophrenia and whether patients with schizophrenia could comprehend and apply suggested treatment. It was found that females were more likely to express positive attitudes than males for these measures. Nothing else was reported for the relationship between sex and stigmatisation.  Prior to training, there was a significant relationship between personal familiarity with schizophrenia and the measure schizophrenia patients should be kept in hospitals. Participants with an acquaintance that had been diagnosed with schizophrenia were more likely to disagree with this measure. Nothing else was reported for the relationship between personal familiarity with schizophrenia and stigmatisation.  Significant relationships were not found between time point and stigmatisation for eight of the measures, excluding what is the course of schizophrenia? For the remaining measures, significant relationships were identified between time point and stigmatisation. In all of these cases, participants were more likely to express positive attitudes three months after the training sessions. |
| Ucok et al. (2004) | Psychiatrists  (Turkey) | Cross-sectional survey with open-ended questions | Chi-square test of independence | Schizophrenia (label) | Prognosis  Family history of psychiatric disorder | On average participants believed that the likelihood of rehabilitation for schizophrenia was roughly fifty-fifty.  Family history of psychiatric disorder was not found to be significantly related to prognosis. |
| Upshur & Weinreb (2008) | Family physicians  Family nurse practitioners  Family practice and internal medicine residents |  |  |  |  | Nothing more was reported for this study as findings were not reported for family physicians separately. |
| Van Boekel et al. (2015) | GPs  Nurses from mental health and addiction services  Social workers  Psychiatrists  Psychotherapists  Prevention and aftercare specialists from mental health and addiction services  Other unspecified specialists from mental health and addiction services  General population  Clients in treatment for SUD  (Netherlands) | Cross-sectional survey | Multiple regression analysis | Substance addiction (label) | Attribution beliefs  Someone with a substance addiction is responsible for this  Someone with a substance addiction is in control of this addiction  Someone with a substance addiction can be treated successfully  Substance addiction is a disease  Substance addiction is the consequence of weakness  Perceived rehabilitation chances  Expectation of chance to find a place to live  Expectation of chance to maintain a normal job  Expectation of chance to have a relationship  Other stereotypical beliefs  Intelligent  Trustworthy  Aggressive  Able to maintain a regular job  Tend to cause disturbances  Self-neglecting  Tend to be criminals  Social distance  Age  Sex  Social desirability | GPs agreed more that someone with a substance addiction is responsible for their addiction. However, GPs agreed less that someone with a substance addiction is in control of this addiction, and substance addiction is the consequence of weakness. GPs also agreed more that someone with a substance addiction can be treated successfully, and substance addiction is a disease. Despite these positive attitudes, GPs expressed negative attitudes for most of the remaining stigmatisation measures and items. The only exceptions to this were GPs responded neutrally to the intelligent and able to maintain a regular job stereotypes, and responded positively to the tend to be criminals stereotype.  For GPs, all of the other stereotypical beliefs and most of the attribution beliefs were not found to be significant predictors of social distance. However, perceiving people with substance addiction as responsible for their addiction was a significant predictor of increased social distance. This was the only attribution belief found to be a significant predictor of social distance.  For GPs, age, sex, and social desirability were not found to be significant predictors of social distance.  Other relevant findings were excluded from this table as they were not reported for mental health professionals separately. |
| Van Boekel et al. (2014) | GPs  Nurses from mental health and addiction services  Social workers  Psychiatrists  Psychotherapists  Staff, prevention, aftercare, and management specialists from mental health and addiction services  Other unspecified specialists from mental health and addiction services  (Netherlands) | Cross-sectional survey | - | Substance addiction (label) | MCRS (only items relevant to stigmatisation were included in this table)  Insurance plans should cover patients like this to the same degree that they cover patients with other conditions  There is little I can do to help patients like this  I feel especially compassionate towards patients like this  Patients like this irritate me  I wouldn’t mind getting up on call nights to care for patients like this  Treating patients like this is a waste of medical dollars  Patients like this are particularly difficult for me to work with  I prefer not to work with patients like this  Attribution beliefs  Someone with a substance addiction is responsible for this  Someone with a substance addiction is in control of this addiction  Someone with a substance addiction can be treated successfully  Substance addiction is a disease  Substance addiction is the consequence of weakness  Emotions  Anger  Fear  Pity | Most GPs expressed positive attitudes for the majority of the MCRS items. The only exceptions to this were roughly half of the GPs disagreed that they feel especially compassionate towards patients like this, and most GPs disagreed that they wouldn’t mind getting up on call nights to care for patients like this.  GPs agreed more that someone with a substance addiction is responsible for their addiction. However, GPs agreed less that someone with a substance addiction is in control of this addiction, and substance addiction is the consequence of weakness. GPs also agreed more that someone with a substance addiction can be treated successfully, and substance addiction is a disease.  GPs reported feeling fear and anger less, and pity more.  Other relevant findings were excluded from this table as they were not reported for mental health professionals separately. |
| Van Dorn et al. (2005) | Psychiatry clinicians  Psychology clinicians  Social work clinicians  Case managers |  |  |  |  | Nothing more was reported for this study as findings were not reported for mental health professionals separately. |
| Vendsborg et al. (2013) | Psychologists  Occupational therapists  Social workers  Unspecified doctors from psychiatric units  Unspecified nurses from psychiatric units  Nurse aides  Administrative staff  (Denmark) | Cross-sectional survey | - | Mental illness in general (label)  Schizophrenia (label) | Causal attributions  Prognosis  People with schizophrenia are dangerous more often than not  The public does not need to be protected from people with schizophrenia  If a consultant psychiatrist instructed me to treat people with a mental illness in a disrespectful manner, I would not follow their instructions  I feel as comfortable talking to a person with schizophrenia as I do talking to a person with a physical illness  I would use the terms crazy, nutter, mad, etc to describe people with schizophrenia who I have seen in my work | Most of the doctors attributed schizophrenia to serious traumas, and just under half attributed schizophrenia to relations in the family. All of the doctors believed schizophrenia is a disease of the brain, and caused by a genetic disposition and a combination of the mentioned factors.  Most of the doctors disagreed that people with schizophrenia can never reach a good quality of life. However, most of the doctors also believed that schizophrenia is a chronic illness.  Most doctors did not agree that people with schizophrenia are dangerous more often than not, and most doctors agreed that the public does not need to be protected from people with schizophrenia.  Only a small subset of doctors agreed that if a consultant psychiatrist instructed me to treat people with a mental illness in a disrespectful manner, I would not follow their instructions.  For the remaining two measures of stigmatisation, most doctors did not express stigmatisation.  Other relevant findings were excluded from this table as they were not reported for mental health professionals separately. |
