# Supplementary material for: The stigmatization of mental illness by mental health professionals: Scoping review and bibliometric analysis
Source: PLoS One. 2023 Jan 20;18(1):e0280739. doi: 10.1371/journal.pone.0280739 (PMC9858369; doi:10.1371/journal.pone.0280739)
Supplement: S13 Appendix — (DOCX) [file pone.0280739.s013.docx]

| **Authors (year)** | **Populations**  **(countries)** | **Research methods** | **Analytical approaches** | **Disorders** | **Variables and measures** | **Findings** |
| --- | --- | --- | --- | --- | --- | --- |
| Walsh et al. (2005) | Social workers  (USA) | Experiment | Between-groups ANOVA | Schizophrenia (label)  Major depression (label)  Eating disorder (label)  Dissociative disorder (label) | Causal attributions  Years of professional experience  10 or less  11 to 20  21 to 29  30 or more  Age (levels were not specified)  Sex  Racial-ethnic background (levels were not specified)  Professional setting  Not-for-profit  Independent private practice  Public organisations  Other  Professional function (levels were not specified) | Participants attributed schizophrenia to biological causes more, followed by major depression (one biological aetiology item also included environmental factors beyond parental control). In comparison, participants attributed dissociative disorder to parenting and family dynamics more, followed by eating disorder.  Schizophrenia was attributed to biological factors significantly more than eating disorder and dissociative disorder, and major depression was attributed to biological factors significantly more than dissociative disorder. The difference between schizophrenia and major depression was not found to be statistically significant, and the difference between major depression and eating disorder was not found to be statistically significant. Whether there was a significant difference between eating disorder and dissociative disorder was not reported.  All other variables were not found to have a significant impact on causal attributions. |
| Wang et al. (2017) | Primary care physicians  Primary care nurses  Village doctors  Other unspecified primary care workers |  |  |  |  | Nothing more was reported for this study as findings were not reported for primary care physicians separately. |
| Waugh et al. (2017) | Unspecified nurses from mental health and non-mental health services  An occupational therapist  A therapy assistant practitioner  A physiotherapist  A health care assistant  A speech and language therapist |  |  |  |  | Nothing more was reported for this study as findings were not reported for mental health professionals separately. |
| Welch et al. (2015) | Family physicians  Internists |  |  |  |  | Nothing more was reported for this study as findings were not reported for family physicians separately. |
| Weller & Grunes (1988) | Unspecified nurses from a psychiatric hospital  Other unspecified nurses  (Israel) | Cross-sectional survey | - | Mental illness in general (label) | AMI questionnaire  One can always tell a mentally ill person by his physical appearance  In order to work with the mentally ill, there is no need for professional knowledge  The mentally ill, with a number of exceptions, cannot tell the difference between good and bad  Mental illness is genetic  The mentally ill should be prevented from having children  Mental illness cannot be cured  One should avoid all contact with the mentally ill  Psychiatric hospitals should not be located in residential areas  Those who work in hospitals for the mentally ill do so because they have no other choice  The mentally ill should not get married  Life has no value for the mentally ill  It is not necessary to consider the opinion of a person who has been released from a mental hospital  The mentally ill should live only among themselves  There are people who were never in a mental hospital and are more disturbed than those who are in a mental hospital  Once crazy, always crazy  It is not necessarily true that a person who was once in a hospital for the mentally ill will continually have to return there  Usually, there is no way of telling when seeing a person walking in the street if he was ever in a hospital for the mentally ill  Very few, if any, mentally ill people are capable of true friendships  Mentally ill people should be prevented from walking freely in public places  One should hide his/her mental illness from his/her family  Mentally ill people who do not get well have no one to blame but themselves  Mentally ill people who are not hospitalised should be prevented from walking freely in public places at night  The mentally ill should not be allowed to make decisions, even those concerning routine events  Every mentally ill person should be in an institution where he/she will be under supervision and control | Overall, psychiatric nurses expressed more negative attitudes towards mental illness.  Other relevant findings were excluded from this table as they were not reported for psychiatric nurses separately. |
| Werner & Araten-Bergman (2017) | Social workers  (Israel) | Experiment  Vignettes were used | Within-subjects ANOVA  Correlation analysis | Mental illness in general and schizophrenia (description and label)  Intellectual disability (description)  Mental illness in general and comorbid intellectual disability and schizophrenia (description and label) | AQ-27 (items were not specified)  Perceived personal responsibility  Perceived dangerousness  Anger  Fear  Pity  Segregation  Avoidance  Coercion  Helping | Participants mostly expressed less stigmatisation across the AQ-27 factors. However, intellectual disability and comorbid intellectual disability and schizophrenia (this vignette also included the label mental illness) elicited more avoidance, and schizophrenia (this vignette also included the label mental illness) elicited roughly neutral responses. Also, all of the mental disorders elicited more coercion.  For each AQ-27 factor, a hierarchical pattern emerged, in which participants stigmatised the different mental disorders to varying degrees. No mental disorder was consistently stigmatised more than any other disorder across the AQ-27 factors. Disorder was not found to have a significant impact on anger, pity, avoidance, and helping. Disorder was found to have a significant impact on the remaining AQ-27 factors. For all of these factors, schizophrenia was stigmatised significantly more than intellectual disability, and in one case was stigmatised significantly more than comorbid intellectual disability and schizophrenia. In all other cases, schizophrenia, and comorbid intellectual disability and schizophrenia were not found to be significantly different. For the AQ-27 factors in which disorder had an impact, comorbid intellectual disability and schizophrenia was stigmatised significantly more than intellectual disability for most of the factors. Comorbid intellectual disability and schizophrenia, and intellectual disability were not found to be significantly different in only one case.  Perceived personal responsibility was not found to be significantly correlated with any of the behavioural intentions.  Perceived dangerousness was significantly positively correlated with segregation, avoidance, and coercion, and significantly negatively correlated with helping. The only exception to this was perceived dangerousness was not found to be significantly correlated with helping for schizophrenia.  Anger and fear were significantly positively correlated with segregation, avoidance, and coercion, and significantly negatively correlated with helping.  Pity was significantly positively correlated with coercion and helping, and significantly negatively correlated with avoidance. The only exception to this was pity was not found to be significantly correlated with avoidance for comorbid intellectual disability and schizophrenia. Pity was not found to be significantly correlated with segregation. |
| Williams (2009) | Unspecified doctors from mental health services  Unspecified nurses from mental health services  Psychologists  Female occupational therapists  Social workers  Physiotherapists  Other unspecified professionals from mental health services  (England) | Cross-sectional survey | - | Mental illness in general (label)  Substance misuse problems (label) | Causal attributions  Prognosis | The measures were unable to be interpreted with the information provided. However, some of the nurses commented that substance misuse rarely leads to mental illness.  Other relevant findings were excluded from this table as they were not reported for mental health professionals separately. |
| Winkler et al. (2016) | GPs  Psychiatrists  Neurologists  Paediatricians  Gynaecologists  Obstetricians  Other unspecified medical doctors |  |  |  |  | Nothing more was reported for this study as findings were not reported for mental health professionals separately. |
| Wlodarczyk et al. (2018) | GPs  (Australia) | Focus groups | Template analysis | BPD (label) | Perceived difficulty  Feeling overwhelmed  Exhaustion  Avoidance  People with BPD frequently cancel appointments  Frustration  Discomfort  Intention to provide quality care  The personhood of the patient with BPD must be maintained | Participants described people with BPD as difficult in general, and it was suggested that this elicited feelings of being overwhelmed and exhausted. Further, one participant stated that the perception of people with BPD being difficult made them not want to work with BPD.  Avoidance of BPD was also explained by the participants as arising from the perception that people with BPD frequently cancel appointments. It was also suggested that this perception elicited frustration.  GPs described their experience with BPD as uncomfortable in general.  Despite the previously noted findings, GPs were still motivated to provide quality care to people with BPD, and one participant stated that the personhood of the patient with BPD must be maintained. |
| Woollaston & Hixenbaugh (2008) | Psychiatric nurses  (England) | Semi-structured interviews | Thematic analysis | BPD (label) | People with BPD are powerful  People with BPD are dangerous  People with BPD are unrelenting  People with BPD are disruptive  People with BPD are an unstoppable force of destruction  People with BPD are time and energy consuming  People with BPD are demanding and draining  People with BPD are like a whirlwind that sucks you in  People with BPD are aggressive to themselves and others  People with BPD cause me personal distress  Prognosis  Discouragement  Frustration  General dislike in working with BPD  Professional exposure to BPD  Perceived avoidance  Perceived sympathy  Perceived general dislike  People with BPD are manipulative and dishonest  Feeling of being used and devalued  Vigilance | People with BPD were described as powerful, dangerous, unrelenting, disruptive, and an unstoppable force of destruction. People with BPD were also described as being time and energy consuming, demanding, draining, and like a whirlwind that sucks you in.  People with BPD were described as aggressive to themselves and others, and as causing personal distress.  BPD was described as an unhelpable condition, and participants stated that this made them feel discouraged and frustrated. One participant stated that the chronic nature of BPD made them not like working with BPD. However, some participants expressed optimism in the ability of people with BPD to change.  A participant suggested that professional exposure to BPD made them less optimistic in their ability to change.  One participant perceived avoidance of BPD in other psychiatric nurses. Participants also perceived sympathy in other psychiatric nurses, and a general dislike of people with BPD in another group of psychiatric nurses.  Participants expressed the belief that people with BPD are manipulative and dishonest, and this perception made them feel used and devalued. This perception also motivated the participants to be vigilant around people with BPD. |
| Worthington & Atkinson (1993) | Psychologists  (USA) | Cross-sectional survey  Vignettes were used | Chi-square test of independence | Adjustment disorder (description)  Identity disorder (description) | Causal attributions  Perceived responsibility of cause  Perceived responsibility of problem solution | Participants attributed the disorders to a range of causes with varying proportions. For adjustment disorder, the most common causes were specific trauma and unresolved feelings. Adjustment disorder was not attributed to genetics, social isolation, sick society, or physical illness by any of the participants. For identity disorder, the most likely causes were lack of self-understanding and unresolved feelings. Identity disorder was not attributed to genetics, specific trauma, social isolation, or sick society by any of the participants. Differences between the disorders for causal attributions were not examined with inferential statistics.  Most participants attributed a low level of cause to the person with adjustment disorder, and roughly half of the participants attributed a high level of cause to the person with identity disorder. All participants attributed a high level responsibility to both disorders for the solution to their problems.  There was a significant relationship between disorder and perceived responsibility of cause. The person with identity disorder was more likely to be considered responsible for their disorder, compared to adjustment disorder. |
| Wright & Klein (1966) | Social workers  Unspecified nurses  Physical medicine and rehabilitation service therapists  Unspecified physicians  Physical medicine and rehabilitation service assistants  Nursing assistants  Unspecified registrars  Housekeeping personnel  Dieticians  Engineers  Laundry personnel  Other unspecified hospital personnel  (USA) | Cross-sectional survey | - | Mental illness in general (label) | What percentage of the people going to a mental hospital will stay for life?  What percentage of hospitalised mental patients need to be locked up because they are dangerous?  What percentage of hospitalised mental patients need to be encouraged to leave the hospital because they lack confidence?  What percentage of hospitalised mental patients can be allowed to walk around the grounds without being watched?  What percentage of hospitalised mental patients have too much energy and are too active most of the time?  What percentage of hospitalised mental patients can be allowed to go to town by themselves on weekends?  What percentage of those locked up need to wear straight jackets or be in a padded room?  What percentage of hospitalised mental patients would sit around all day?  What percentage of hospitalised mental patients would hurt themselves if they got out?  What percentage of hospitalised mental patients would hurt someone else if they got out?  What percentage of the patients discharged from the nearest mental hospital should settle in your town if they want to?  What percentage of the people discharged from a mental hospital are able to hold a fulltime job?  What percentage of the people discharged from a mental hospital should be allowed to drive a car?  What percentage of the people discharged from mental hospitals should return to their home towns if they want to?  What percentage of the people discharged from a mental hospital are able to do the type of work they did before they entered the hospital?  What percentage of the general population of the country will need to go to a mental hospital?  What percentage of hospitalised mental patients could be given the freedom to come and go like patients in a regular hospital?  What percentage of the patients in mental hospitals are there voluntarily? | Overall, social workers stigmatised mental illness.  Other relevant findings were excluded from this table as they were not reported for social workers separately. |
| Yuan et al. (2017) | Unspecified doctors from a psychiatric facility  Unspecified nurses from a psychiatric facility  Psychologists  Social workers  Occupational therapists  Case workers  Pharmacists  Physiotherapists |  |  |  |  | Nothing more was reported for this study as findings were not reported for mental health professionals separately. |
