# Supplementary material for: The stigmatization of mental illness by mental health professionals: Scoping review and bibliometric analysis
Source: PLoS One. 2023 Jan 20;18(1):e0280739. doi: 10.1371/journal.pone.0280739 (PMC9858369; doi:10.1371/journal.pone.0280739)
Supplement: Attachment — Submitted filename: Response to Reviewers.docx [file pone.0280739.s017.docx]

September 7, 2022

Subject: Submission Revised Manuscript ID PONE-D-22-02618

Dear Editor of PLOS One

Accompanying this letter please find a revised version of ID PONE-D-22-02618, ' The Stigmatization of Mental Illness by Mental Health Professionals: Scoping Review and Bibliometric Analysis’. We have carefully attempted to revise our manuscript responsive to the comments of the editor and reviewer. We are grateful to have had the opportunity to do so and we believe that our efforts have improved the quality and clarity of the manuscript. Below we respond to the concerns and specific points. We hope that you now find the revised manuscript acceptable for publication in PLOS One.

**Additional requirements**

1. *Please ensure that your manuscript meets PLOS ONE's style requirements, including those for file naming.*

We have ensured that the manuscript meets PLOS One’s style requirements.

**Editors’ comments**

1. *Thank you for providing a completed PRISMA checklist. Please update the checklist to use section and paragraph numbers, rather than page numbers. Please add the following statement, or similar, to the Methods: "This study is reported as per the Preferred Reporting Items for Systematic Reviews and Meta-Analyses extension for scoping reviews (PRISMA-ScR) guideline (S16 Appendix)."*

The statement "This study is reported as per the Preferred Reporting Items for Systematic Reviews and Meta-Analyses extension for scoping reviews (PRISMA-ScR) guideline (S16 Appendix)." has been added to the Method section, and the PRISMA checklist has been updated to use section and paragraph numbers (the updated checklist has been attached as an additional file). As PLOS One style requirements do not include section numbers, the section numbers in our PRISMA checklist refer to each of the major manuscript sections in chronological order (i.e., the title page is section 1, the abstract is section 2, the introduction is section 3 etc.) and paragraph numbers refer to each paragraph in chronological order within each section. We apologise for these oversights.

**Reviewer’s comments**

1. *I think the objective section is too long, and much of what is said in it can be said in previous sections. In order to facilitate reading, I recommend that authors in this section limit themselves to describing the objective or objectives as clearly as possible, in a concise and direct manner. To justify the objective, the previous sections must be used.*

The Objectives section has been shortened and justifications for the objectives have been moved to the Rationale section.

2. *Were the searches carried out only in the Title/Abstract fields of the databases, or also in controlled languages (Thesaurus, Mesh...), what do the authors mean when they say that they searched for keywords?*

The searches were only carried out on the title/abstract/keyword fields of the databases. The search was not conducted in controlled languages. The number of results generated from the searches that we carried out on the title/abstract/keyword fields was quite large. Thus, a search for each of the search string terms in controlled languages would have produced an infeasible number of results to screen.

3. *The process of selecting articles for the review is confusing. It is understood that the search was done in three moments (it is a good idea to update the search if a long time has passed since the first time it was done), but it is difficult to understand, as it is currently written, the total number of records found, the eliminated, etc, since in figure 1 (and in the text) only data from the first phase are provided. In fact, according to the figure, 184 works are included, then it is said that 300, later than 197... In short, I recommend the authors to simplify the explanation given for this process, including the total data of the three phases.*

We agree with this suggestion and thank the reviewer for this feedback. The figure has been changed to cover all articles included in the scoping review. The figure now includes articles identified through the reference lists of relevant articles from the first database searches and articles identified through the second and third databases searches. Within text, the figure citation was also moved further down to reflect these changes. The distinction between the 300 articles included in the scoping review and the 197 that were isolated in the Results section is discussed in text. We believe that addressing this in text is appropriate.

4. *In the final table of Fig 1 the authors refer to "qualitative synthesis". I have doubts about whether what is being done is a qualitative synthesis. I think it is better to say "**articles included in the scoping review"*

The figure has been changed to reflect this suggestion.

5. *On page 23, line 504-507 the authors refer to the variability of the studies and the difficulties in comparing them and obtaining global results. Do the authors believe that this may be related to the breadth of inclusion criteria for their review (multiple professionals, various types of disorders, different types of studies, both qualitative and quantitative)? Perhaps in discussion or in limitations they could include some comment about it.*

We agree that it is useful to clarify these distinctions and we have included the following sentence in the Results “Even within categories that ought to be homogeneous this level of variability was still evident”.

6. *The beginning of the discussion (pg 25 lines 546-565) is very long and is repetitive with the introduction. It is true that the introduction should begin with a brief reminder of the objective, but repetition of ideas should be avoided. I recommend making it shorter, remembering the aim and objectives of the study.*

The beginning of the Discussion has been shortened and now focuses on the aim and objectives of the study.

7. *A bibliometric analysis (not scoping review) on stigma in nursing professionals and students (exclusively) has recently been published (https://doi.org/10.3390/ijerph19031839). Although it is not exactly the same objective as this manuscript, I think it could be interesting for the authors to take it into account and perhaps compare results.*

We thank the reviewer for pointing out this additional reference. We have included this reference in the Objectives section. Although the results were less directly comparable to the present work, we note the similarity of purpose and increasing use of bibliometric analyses in the field of mental illness stigma.

Once again, we would like to thank the editor and reviewer for the opportunity to revise this paper and for considering it for publication. We look forward to hearing of the outcome.

Kind Regards,

The Authors
