# Supplementary material for: The stigmatization of mental illness by mental health professionals: Scoping review and bibliometric analysis
Source: PLoS One. 2023 Jan 20;18(1):e0280739. doi: 10.1371/journal.pone.0280739 (PMC9858369; doi:10.1371/journal.pone.0280739)
Supplement: Attachment — Submitted filename: renamed_e7cdf.docx [file pone.0280739.s018.docx]

Thank you for the opportunity to review this article. The authors have done an exhaustive research work, analysing a large number of articles with very diverse methodologies and samples. Here are a few suggestions that could

1.	The objectives section is still too long. It is not necessary to provide so much information as it is already provided in the previous sections.

2.	In the section Populations, time periods, and the extent of research results a lot of information previously given is repeated.

Lines 558 to 570 of the section summary and interpretation of the reults are repeated in the section Populations, time periods, and the extent of research. You have already provided the data in the previous section in an exhaustive way. I believe that indicating only the general results simplifies the reading.

3.	The bibliometric analysis. Some of the results are repeated in the discussion without justification.

Direct citation analysis is used to determine the impact of particular articles or authors. Therefore, I think that the work would be improved if you would describe in more detail the results obtained: how many clusters you have obtained, the main authors cited, indicating the number of citations and the clusters that are closest to each other. In this way, in the discussion you can try to find patterns in the use of scientific information by groups of authors or if collaboration is scarce on certain topics.

In relation to the above, the paragraph on lines 536 -538 "clusters were often characterised by a theme. For instance, the aforementioned red cluster contained studies that were all about either borderline personality disorder or personality disorders in general" is not a direct result. You should move it to the discussion and justify with the authors' citations why you come to that conclusion or why you think the authors investigate similar themes.

4.	The discussion section in general is too long and often repeats information without providing interpretation or comparison with other reviews. It would improve the readability if it were shorter.
