# Supplementary material for: The stigmatization of mental illness by mental health professionals: Scoping review and bibliometric analysis
Source: PLoS One. 2023 Jan 20;18(1):e0280739. doi: 10.1371/journal.pone.0280739 (PMC9858369; doi:10.1371/journal.pone.0280739)
Supplement: Attachment — Submitted filename: Response to Reviewers.docx [file pone.0280739.s019.docx]

November 15, 2022

Subject: Submission Revised Manuscript ID PONE-D-22-02618

Dear Editor of PLOS One

Please find attached our revised manuscript for submission. We are pleased that our previous revisions have addressed most of the reviewers’ concerns. We list in point form below our response to the remaining issues identified.

**Reviewer 2**

1. *The objectives section is still too long. It is not necessary to provide so much information as it is already provided in the previous sections.*

The Objectives section has been further shortened to exclude unnecessary information.

2. *In the section Populations, time periods, and the extent of research results a lot of information previously given is repeated. Lines 558 to 570 of the section summary and interpretation of the reults are repeated in the section Populations, time periods, and the extent of research. You have already provided the data in the previous section in an exhaustive way. I believe that indicating only the general results simplifies the reading.*

Most of this section has been removed. This section now only includes sentences about inappropriate samples and about excluding a proportion of the literature. Consequently, the subheading for this section has been changed to Inappropriate Samples.

3. *The bibliometric analysis. Some of the results are repeated in the discussion without justification. Direct citation analysis is used to determine the impact of particular articles or authors. Therefore, I think that the work would be improved if you would describe in more detail the results obtained: how many clusters you have obtained, the main authors cited, indicating the number of citations and the clusters that are closest to each other. In this way, in the discussion you can try to find patterns in the use of scientific information by groups of authors or if collaboration is scarce on certain topics. In relation to the above, the paragraph on lines 536 -538 "clusters were often characterised by a theme. For instance, the aforementioned red cluster contained studies that were all about either borderline personality disorder or personality disorders in general" is not a direct result. You should move it to the discussion and justify with the authors' citations why you come to that conclusion or why you think the authors investigate similar themes.*

The results of the direct citation analysis have been described in more detail as suggested by the reviewer (i.e., how many clusters you have obtained, the main authors cited, indicating the number of citations, and the clusters that are closest to each other). Interpretations about the themes of different clusters have been removed from the Results. Given these interpretations were already in the Discussion, they have remained in this section. As well, the points in this paragraph have been reordered for clarity.

4. *The discussion section in general is too long and often repeats information without providing interpretation or comparison with other reviews. It would improve the readability if it were shorter.*

In general, the Discussion section has been shortened to exclude unnecessary repetition of the results.

**Reviewer 3**

1. *The methodological procedures related to bibliometric analysis are very brief. This aspect can be improved, to enrich your manuscript. Please consult the following references that can help you in writing the method:*

*Bhandari, A. (2022). Design Thinking: from Bibliometric Analysis to Content Analysis, Current Research Trends, and Future Research Directions. Journal of the Knowledge Economy, 1-56.*

*Donthu N, Kumar S, Mukherjee D, Pandey N, & Lim W M (2021a). How to conduct a bibliometric analysis: An overview and guidelines. Journal of Business Research, 133(1), 285-296.*

*Donthu, N., Kumar, S., Pandey, N., & Lim, W.M. (2021b). Research constituents, intellectual structure, and collaboration patterns in Journal of International Marketing: An analytical retrospective. Journal of International Marketing, 29(2), 1-25.*

We thank the reviewer for these suggestions, and we have consulted these sources. We see that our analysis is largely consistent with the suggested approach. However, we have brought together information about the analyses into the section of the Method about our bibliometric analysis. This, in conjunction with extra material suggested by other reviewers, makes clearer the steps in the analysis for the reader.

Once again, we would like to thank the editor and reviewers for the opportunity to revise this paper and for considering it for publication. We look forward to hearing of the outcome.

Kind Regards,

The Authors
