# Supplementary material for: The stigmatization of mental illness by mental health professionals: Scoping review and bibliometric analysis
Source: PLoS One. 2023 Jan 20;18(1):e0280739. doi: 10.1371/journal.pone.0280739 (PMC9858369; doi:10.1371/journal.pone.0280739)
Supplement: Attachment — Submitted filename: Response to Reviewers.docx [file pone.0280739.s020.docx]

December 22, 2022

Subject: Submission Revised Manuscript ID PONE-D-22-02618

Dear Editor of PLOS One

Please find attached our revised manuscript for submission. We are pleased that our previous revisions have addressed most of the reviewers’ concerns. We list in point form below our response to the remaining issues identified.

**Reviewer 3**

1. *In the objectives section, line 154 and 155, it says: "The aim of this review is to examine the state of all available research on the stigmatisation of mental illness by mental health professionals". I would change the word "all avaiable" for another expression that is not so absolute.*

We thank the reviewer for this feedback. We have removed the word “all” from the phrase “all available” in both the Objectives section and Discussion section. The aim now reads “to examine the state of available research on the endorsed stigmatization of mental illness by mental health professionals”.

2. *In the results you indicate in lines 522 to 525: Furthermore, in examining these studies, numerous inconsistencies were found between the articles with regard to both the presence and direction of effects. In fact, the only overall finding that was clearly discernible was that mental health professionals endorse both positive and stigmatising reactions to mental illness. However, the conclusions seem to indicate that the results indicate that professionals have stigmatising reactions to mental illness. Lines 774 and 775 Mental health professionals, including clinical psychologists, were found to have stigmatising reactions to mental illness (5, 21-24). If the main result is that it is not possible to have a clear conclusion whether professionals have positive or negative reactions it should be reflected in the findings.*

We understand the reviewer’s confusion with the sentence “In fact, the only overall finding that was clearly discernible was that mental health professionals endorse both positive and stigmatising reactions to mental illness”. Rather than suggesting there was no effect of stigmatization, we intended to illustrate the ambivalence of some of these findings. However, as this was not a key finding of the present scoping review, and other authors have already noted similar results (i.e., mental health professionals expressing ambivalence towards people with mental illness by endorsing both positive and stigmatising reactions), we have removed this sentence. We feel that this removes a potential source of contradiction for the reader.

Once again, we would like to thank the editor and reviewers for the opportunity to revise this paper and for considering it for publication. We look forward to hearing of the outcome.

Kind Regards,

The Authors
